# Structure of Layers Formed by [2-(3,6-Disubstituted‑9*H*‑carbazol-9-yl)ethyl]phosphonic Acids on Metal Oxides

**DOI:** 10.1021/acsami.6c03387

**Published:** 2026-05-17

**Authors:** Collin A. Sindt, Oliver Wright, Yadong Zhang, Junxiang Zhang, Patteera Funchien, Lars Thomsen, Eliot Gann, Cherno Jaye, Stephen Barlow, Seth R. Marder, Michael F. Toney

**Affiliations:** † Department of Chemical and Biological Engineering, 1877 University of Colorado Boulder, Boulder, Colorado 80303, United States; ‡ Department of Physics, 1877 University of Colorado Boulder, Boulder, Colorado 80303, United States; § Department of Chemistry, 1877 University of Colorado Boulder, Boulder, Colorado 80303, United States; ∥ Renewable and Sustainable Energy Institute, 1877 University of Colorado Boulder, Boulder, Colorado 80303, United States; ⊥ 326623 Australian Synchrotron, ANSTO, 800 Blackburn Road, Clayton, Victoria 3168, Australia; # National Synchrotron Light Source II, Brookhaven National Laboratory, Upton, New York 11973, United States; + Materials Measurement Science Division, 10833 National Institute of Standards and Technology, Gaithersburg, Maryland 20899, United States; ◇ Materials Science Program, 1877 University of Colorado Boulder, Boulder, Colorado 80303, United States

**Keywords:** self-assembled monolayers, hole transport layer, photovoltaics, X-ray reflectivity, NEXAFS, X-ray photoelectron spectroscopy

## Abstract

[2-(9*H*-Carbazol-9-yl)­ethyl]­phosphonic acid (2PACz) and its derivatives are being used extensively as hole-transport layers in organic and perovskite solar cells due to their ability to modify electrode work function, surface wettability, and in some cases, to improve active-layer adhesion, while minimizing interfacial energy losses. The orientation and coverage of surface modifiers significantly impact these functional properties; however, the detailed structure and packing in these overlayers are challenging to investigate, leading to a lack of understanding of how this structure and packing influence performance and limiting the ability to rationally design molecular modifiers. Here, we investigate monolayers of 2-(9*H*-carbazol-9-yl)­ethyl phosphonic acid derivatives (from here on referred to as X-2PACz) on indium tin oxide (ITO) and alpha phase aluminum oxide (α-Al_2_O_3_) using a combination of X-ray photoelectron spectroscopy (XPS), near-edge X-ray absorption fine structure (NEXAFS) spectroscopy, and X-ray reflectivity (XRR). By correlating elemental ratios, molecular orientation, and electron density profiles, we directly quantify surface coverage, layer thickness, and molecular tilt across a series of chemically related monolayers. We find that 2PACz and the X-2PACz derivatives form dense monolayers on α-Al_2_O_3_ and ITO, with similar surface coverages on either substrate that are inversely proportional to molecular steric bulk. These surface coverage results indicate that X-2PACz is sterically limited in its monolayer surface packing density, as opposed to site limited. Despite surface packing density differences between molecules, the NEXAFS data show a constant average molecular orientation of 61° to 65° between the plane of the carbazole and the substrate for all the molecules on both substrates. These results increase our general understanding of 2PACz derivatives as they become increasingly useful in high performance solar cells.

## Graphical abstract

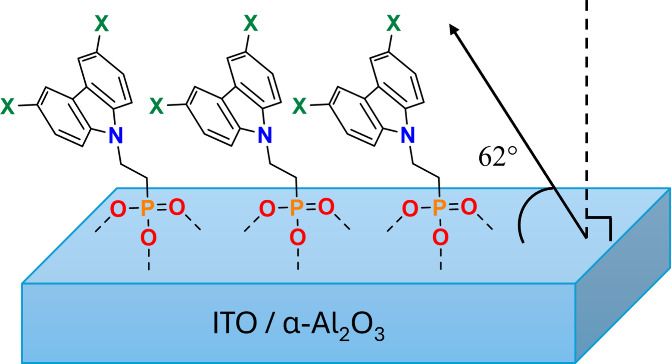

## Introduction

In recent years, phosphonic acid (PA) surface modifiers have become increasingly prevalent in the interfacial design of solar cells and other optoelectronic devices.^1–9^ PAs bind strongly to a variety of metal oxides, including the transparent conductive oxides (TCOs) such as tin-doped indium oxide (ITO) used in organic solar-cell (OSC) and perovskite solar-cell (PSC) fabrication.^10–14^ Coatings of PAs on oxides are often described as self-assembled monolayers (SAMs), although multilayers may be formed under some processing conditions,^15^ and the layers are not necessarily as highly ordered as the term SAM might imply.

The motivation to use PA surface modifiers is largely driven by their ability to tune interfacial properties relevant to device fabrication, performance, and stability. Using these materials, electrode energy levels can be modified to promote enhanced and more selective carrier extraction from PSC and OSC active layers,^1–3,6,13,16–19^ and improved carrier injection in light-emitting diodes.^20–23^ In solar cells, control over electrode surface energy, wettability, and packing density have been achieved by careful selection of surface modifiers and deposition strategy, leading to improved active layer adhesion, more desirable film morphologies, and improved thermal and photostability.^1,2,17,18,24–30^ These factors contribute to the high efficiency and reproducibility of solar cells fabricated with PA-surface modifiers, which, along with their compatibility to green solvents and high-throughput solution processing techniques, makes these materials attractive candidates for the realization of commercially viable OSCs and PSCs.

Solar-cell performance can be influenced by surface modifier chemical structure, deposition method, and the resulting surface structure.^10,11,13,14,16^ Increasing tail-group steric bulk, for example, has been linked to PA-binding-mode changes and coverage differences.^16,31^ The effect of molecular orientation on induced surface dipoles from surface modifiers has also been explored.^10,11,14^ Surface coverage and whether a multilayer or monolayer is formed are other factors of importance that have implications for device performance; for example, Guan et al. recently demonstrated certified OSCs with power conversion efficiencies (PCEs) near 20% using a multilayer of [2-(9*H*-carbazol-9-yl)­ethyl]­phosphonic acid (2PACz, Figure 1, X = H) 2 to 6 nm in thickness, exceeding the PCEs achieved using a monolayer of the same modifier.^15^

**Figure 1:**
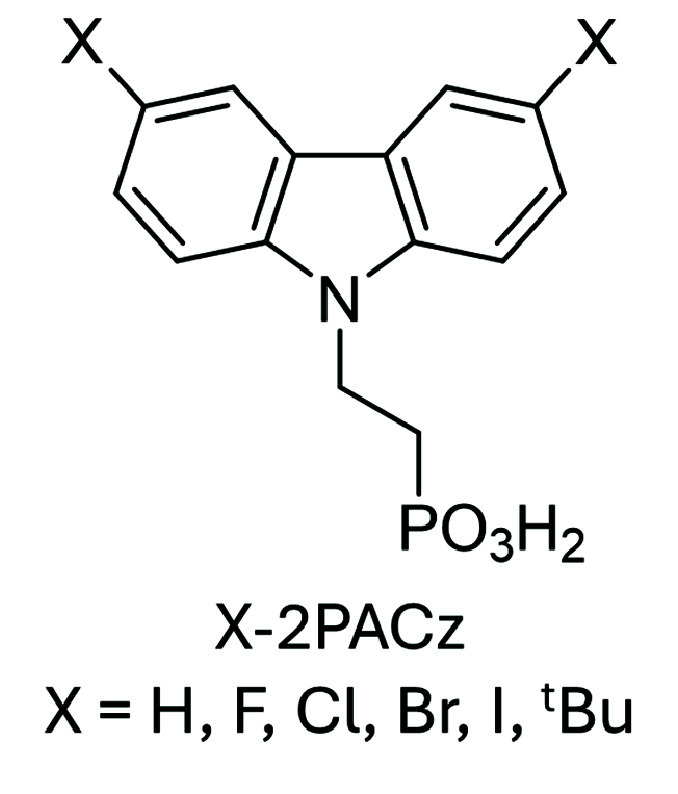
Chemical structures of 2PACz and X-2PACz derivatives studied in this work.

Recently, 2PACz, substituted 2PACz derivatives including those shown in Figure 1 (X-2PACz), and related compounds have repeatedly demonstrated excellent performance when used as hole transport layers (HTLs) between TCOs and the active layers of both PSCs and OSCs.^1–3,19,24,26,32–34^ However, little is known about the detailed structure and packing of the layers formed by these modifiers. Here, we focus on the morphology of films formed by the 2PACz derivatives in Figure 1 on ITO and, as a more planar model substrate, single crystal C-plane alumina, (α-Al_2_O_3_), using the common, facile spin-coating method. We utilize a combined approach of X-ray photoelectron spectroscopy (XPS), X-ray reflectivity (XRR), and near-edge X-ray absorption fine structure (NEXAFS) to investigate PA binding mode, measure surface coverage, and deduce average molecular orientation. We find that, on both α-Al_2_O_3_ and ITO, 2PACz and the derivatives studied form bound monolayers, possess a consistent average molecular orientation, and have surface coverages that decrease as molecular steric bulk increases. These findings demonstrate the similarities in X-2PACz layer structure between the two substrates, highlighting the utility of XRR in measurement of surface packing density and layer thickness. We also show that the sterically limited nature of packing density results in a partially passivated interface with exposed metal centers.

## Experimental Information

### Materials

[2-(9*H*-Carbazol-9-yl)­ethyl]­phosphonic acid (2PACz), [2-(3,6-difluoro-9*H*-carbazol-9-yl)­ethyl]­phosphonic acid (F-2PACz), [2-(3,6-dichloro-9*H*-carbazol-9-yl)­ethyl]­phosphonic acid (Cl-2PACz), 2-[3,6-dibromo-9*H*-carbazol-9-yl)­ethyl]­phosphonic acid (Br-2PACz), [2-(3,6-diiodo-9*H*-carbazol-9-yl)­ethyl]­phosphonic acid (I-2PACz), and [2-(3,6-di­(*tert*-butyl)-9*H*-carbazol-9-yl)­ethyl]­phosphonic acid (^t^Bu-2PACz) were synthesized according to previously published methods.^3,23^ Solvents and reagents were purchased from Sigma-Aldrich and used as received.

### Film Preparation

20 mm × 20 mm ITO films were procured from Colorado Concept Coatings LLC with a square resistance of 20 ohms. These were cut down to 10 mm × 10 mm for experimentation. 10 mm × 10 mm single side polished α-Al_2_O_3_ substrates were purchased from AdValueTech (stock number SS-1SC-1010-50). Both substrate types were cleaned using a 4-part sonication regime using sequential sonications in 1% Solujet phosphate-free detergent in DI water, DI water, acetone, and isopropyl alcohol for 10 min each. In the case of ITO, any reduction in size of substrates was done prior to cleaning. Then, samples were UV-Ozone (UVO) treated in batches for 15 min each, followed by immediate deposition of X-2PACz solution (1 mmol/L in absolute ethanol) in open air. This batch method minimizes time between the end of UVO treatment and deposition of monolayers, minimizing the degradation of the preferential surface condition generated by UVO which allows for better adhering of the SAM molecules.^11^

X-2PACz solutions were sonicated at 30 °C immediately before deposition to reduce the influence of aggregating micelles, which have been shown to be present in 2PACz solutions.^18^ Once deposited, X-2PACz solution was allowed to reside on the surface of the substrate for 10 s, followed by a spin cycle of 314 rad/s for 30 s. Samples were then annealed in air for 10 min at 130 °C, followed by an additional washing step in which 3 instances of 150 μL of ethanol were dynamically applied to the substrate while spinning at 628 rad/s, followed by a final annealing/drying step at 130 °C for 5 min. Samples were characterized as soon as possible after fabrication to minimize the influence of ambient contaminants which would otherwise impact the surface sensitive measurements used.

### X-ray Photoelectron Spectroscopy (XPS)

XPS measurements were carried out using a Kratos Analytical AXIS Supra with a monochromatic Al K_α_ source and a 180° double-focusing hemispherical analyzer. Elemental region scans were collected using a pass energy of 20 eV and an X-ray current of 20 mA. P 2p scans were collected with a step size of 0.05 eV and a dwell time of 425 ms. In 3d scans were collected with a step size of 0.1 eV and a dwell time of 300 ms. Survey scans were conducted using a pass energy of 160 eV, a step size of 1 eV, and a dwell time of 100 ms. Scans were collected with an X-ray incidence angle of 45° to the surface normal, with takeoff angles of 0°, 40°, 55°, and 70° used depending on the acquisition. Samples on both ITO and α-Al_2_O_3_ were attached using copper clips, with charge neutralization used for samples on α-Al_2_O_3_. Peak fitting for all samples was performed in CasaXPS, with information on peak-fitting parameters and select individual spectra listed in the Supporting Information Section S6.

### X-ray Reflectivity (XRR)

XRR was conducted using a Rigaku SmartLab X-ray Diffractometer in ambient conditions over a range from 2θ = 0° to 2θ = 13° using 10 min scan durations. All data were fitted in GenX-3 ^35^ using the differential evolution algorithm and a multilayer fit for each X-2PACz molecule, as depicted in the Supporting Information Section S2. To determine goodness of fit, the built-in logarithmic figure of merit (FoM) function in GenX was used.^35^ This compares the logarithmic difference between measured and simulated intensities, and minimizes the expression given in eq 1:

$${FOM}_{\log}=\frac{1}{N}\underset{i}{\sum }{\left[\log_{10}\left(I_{\exp,i}\right)-\log_{10}\left(I_{sim,i}\right)\right]}^{2}$$ 1

where *I*_*exp,i*_ and *I*_*sim,i*_ are the experimental and simulated intensities at each point i, respectively, and *N* is the number of data points. This method allows the comparison of data over many orders of magnitude, and is common for fitting reflectivity data.^35^

### Near-Edge X-ray Absorption Fine Structure (NEXAFS)

NEXAFS data were acquired at the National Synchrotron Radiation Lightsource II (NSLS-II) beamline 7-ID1/SST1 and the Australian Synchrotron soft X-ray spectroscopy beamline.^36^ Both beamlines allow for optimal tuning of acquisition parameters to balance resolution at the carbon K-edge and high throughput. A combination of total and partial electron yield (TEY and PEY, respectively) were used as data acquisition modes for these experiments. Data acquisition and reduction were approached using documented methods in literature.^37–39^ To assess whether beam damage occurred during acquisition, samples were exposed to a forward and reverse scan over the energy range used in this experiment. No change was observed between the two scans, so beam damage on the time scale of these acquisitions is negligible.

In each setup, the current response from a gold-coated mesh upstream of the sample chamber, which intercepted a fraction of the beam, was used as a normalization reference for beam intensity variations. The influence of carbon contamination on the gold mesh was accounted for by further acquiring independent scans of a photodiode once per sample bar or every (10 to 12) samples scanned. The double normalization of each spectrum is treated according to Equation S6. Further information on treatment and normalization of NEXAFS data can be found in the Supporting Information Section S3.

Three different endstations were used for this experiment: the RSoXS and NEXAFS endstations at the NSLS-II beamline 7-ID1/SST1, and the high-throughput NEXAFS endstation at the Australian Synchrotron. TEY collection mode was used for all samples on ITO, and PEY collection mode was used for all samples on α-Al_2_O_3_. TEY collection mode, while typically better data quality than PEY, necessitates a conductive substrate to measure the drain current which replaces electrons lost to photoemission. TEY is therefore amenable to samples on ITO. For α-Al_2_O_3_ however, TEY data collection is not useful due to both the incompatibility of the nonconductive substrate and the influence of charge compensation used for these substrates which would conflict with the TEY signal. PEY collection mode measures emitted photoelectrons and can filter out the effect of charge compensation on the signal and therefore is the preferred collection mode for these nonconductive substrates. Descriptions of these collection modes can be found in the Supporting Information Section S3. Both collection modes are similarly surface sensitive for the purpose of measuring the average molecular orientation of X-2PACz layers, with their information depth both being on the order of 1 to 3 nm.

Control of the X-ray polarization direction is necessary for determination of average orientations from NEXAFS. In the Australian Synchrotron and NEXAFS endstation at the NSLS-II, this is achieved by a sample orientation change relative to the incident X-ray beam, which subsequently changes the beam spot size. To remove the influence this has on signal intensities, spectra are normalized to the poststep-edge region. This scales each signal to be proportional to the atoms sampled, allowing quantitative comparisons in tilt angles to be made.^39^

All NEXAFS data were analyzed using the Quick Australian Synchrotron NEXAFS Tool (QANT) in Igor Pro 8.^37^

### Ultraviolet Photoelectron Spectroscopy (UPS)

UPS measurements were carried out using a Kratos Analytical AXIS Supra with a Helium 1 source operated at 0.5 mA emission current using a 55 μm aperture collimation mode. Samples measured here are the same as those measured under XPS, with UPS preceding XPS measurements during the same experiment. A step size of 0.1 eV was used with a sweep time of 60 s taking 4 scans of each sample.

### UV–vis Spectroscopy (UV/vis)

UV–vis measurements of 1 mM solutions in absolute ethanol of each X-2PACz molecule were taken using a PerkinElmer Lambda 35 UV/vis spectrometer.

### Atomic Force Microscopy (AFM)

Characterization of ITO substrate surface morphology was done using a Nanosurf Drive AFM using tapping mode under ambient conditions. Images were processed using Nanosurf CX software, and roughness was quantified as the root-mean-square (RMS) roughness (Rq) extracted from the flattened height images.

## Results and Discussion

### X-ray Photoelectron Spectroscopy (XPS) Studies of PA Binding

Analysis of the O 1s XPS spectra of X-2PACz modified ITO and α-Al_2_O_3_ is used to verify successful surface modification.^16,40,41^ This method has been used for the investigation of PA coordination and binding mode atop a variety of metal oxides, including ITO, ZnO, and alumina.^1,7,11,12,18^ For this study, angle-resolved XPS of X-2PACz derivatives were measured on both ITO and α-Al_2_O_3_. Larger takeoff angles in XPS provide greater surface sensitivity and are, therefore, useful in understanding the chemical state of surface species. O 1s XPS spectra can be challenging to fit, as the core-level binding-energy shifts (CLBES) between lattice and surface oxygen species (including surface hydroxyls, bound phosphonic acids, and unbound P–OH groups) are often small, thus requiring theoretical or rigorous experimental support for peak assignments. This study draws from the wide body of literature discussing the O 1s spectra of PA-modified metal oxides, including ITO and alumina.^14,16,40,41^ For PA modification, this literature is more prevalent for ITO than alumina, especially since different forms of alumina (e.g., crystalline vs amorphous) present different lattice O 1s binding energies.^42^ To supplement these documented peak shifts and accurately assign peak shifts for the various oxygen species, standards were measured with XPS including pristine ITO, pristine α-Al_2_O_3_, and 2PACz powder. Using these data, comparisons can be made between the signals from the lattice oxide and surface oxygen species to establish evidence of successful PA surface modification.

Figure 2a shows the comparison of pristine ITO and ITO modified with 2PACz, acquired at a takeoff angle of 70° to accentuate the signal from surface species. Bare ITO was fit with three peaks corresponding to bulk oxygen in the ITO lattice at 530.6 eV, oxygen at the ITO surface with a CLBES relative to the lattice ITO oxygen of 0.5 eV, and surface hydroxide with a CLBES of 1.7 eV.^16,41^ After deposition of 2PACz, surface In–O–P species, of which there are many documented possibilities,^16,31^ are observed at a CLBES of around 1.5 eV along with P=O species (close to In–OH). A peak with a higher CLBES is expected at 3.1 to 3.4 eV for any unbound P–OH.^16,41^ To corroborate these assumptions, 2PACz powder was anaylzed by XPS, shown in Figure S1, with the P=O oxygen located at 532.1 eV (CLBES of 1.5 eV), and P–OH at 533.7 eV (CLBES of 3.1 eV), consistent with the expected CLBES.^16,40^

**Figure 2:**
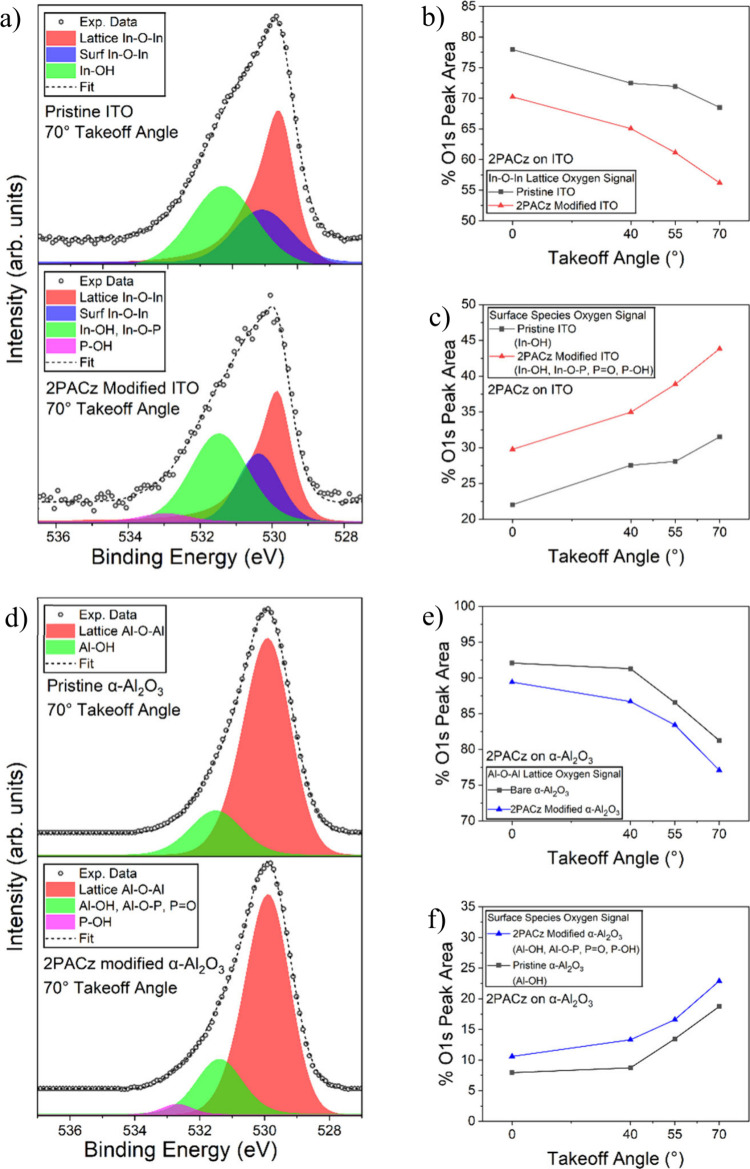
XPS comparison between 2PACz-modified and pristine ITO showing a) the O 1s spectral comparison, b) relative contribution of the lattice oxygen signal, and c) the relative contribution of surface oxygen signals. A comparison between 2PACz modified and pristine α-Al_2_O_3_ showing d) the O 1s spectral comparison, e) the relative contribution of the lattice oxygen signal, and f) the relative contribution of the surface oxygen signals.

Successful functionalization of ITO and alumina produces a decrease in the overall contribution of the lattice oxygen peak due to attenuation through the molecular overlayer and an increase in intensity of higher binding energy peaks associated with coordinated surface species. Successful PA modification, without multilayer accumulation, can be evidenced by the increase in signals associated with bound PAs, without a proportional increase in the peak associated with unbound P–OH. Figure 2b and 2c illustrate this comparison, showing the proportional contribution to the overall O 1s XPS peak by the lattice and surface oxygen species for 2PACz/ITO, respectively, as a function of takeoff angle. The pristine sample shows a higher relative contribution from lattice oxygens, while the 2PACz modified sample shows a higher relative contribution of the surface oxygen species (and by extension concentration) at all takeoff angles. Identical analyses for each X-2PACz molecule on ITO are found in the Supporting Information Figures S2, S3, and S4. These relative intensity variations are not easily visible in the normalized spectra, but this comparison makes surface functionalization more evident.

As noted in the introduction, X-2PACz derivatives on α-Al_2_O_3_ as a model surface were also examined, since α-Al_2_O_3_ is more suitable for techniques requiring a planar surface, particularly XRR (vide infra). Successful PA modification of alumina can also be verified using O 1s XPS spectra, though with some slight differences to the analysis on ITO. Crystalline α-Al_2_O_3_ has a bulk oxygen peak which has a higher binding energy than ITO at around 530.9 eV,^40^ as confirmed by our measurements. Hydroxyls on the surface of alumina are observed at a CLBES from the alumina lattice oxygen peak of around 1.6 eV, while PA species bonded to the surface of alumina, as well the signal from P=O, are seen at a CLBES of 1.5 eV.^40,43^ One peak encompassing all of these surface species was used as the model for 2PACz on alumina, with the addition of a peak to represent P–OH at a binding energy of 533.7 eV, identical to that on ITO, with a CLBES of 2.8 eV relative to the alumina lattice oxygen. These O 1s scans can be compared in an identical fashion to ITO, with the comparison of 2PACz modified and pristine alumina shown in Figure 2d and expanded upon with lattice and surface contributions depicted in Figure 2e and 2f, respectively. We observe an identical trend in that the lattice signal is most prominent for the pristine sample, while the surface species are most prominent for the 2PACz-modified sample, again with the presence of an unbound P–OH peak negligible until the highest takeoff angles. As in the case of ITO, this trend was repeated for each molecule on α-Al_2_O_3_, with similar analyses for each X-2PACz located in the Supporting Information Figures S5, S6, and S7.

To quantify whether each sample is made up of predominantly bound or unbound PA molecules, the peak associated with uncoordinated P–OH is further analyzed. To avoid overinterpretation of weak components, the unique identifiability of the uncoordinated P–OH peak was evaluated using CasaXPS Monte Carlo analysis. For many samples, the Monte Carlo-derived standard deviation of the P–OH peak area exceeded the fitted area, indicating that the uncoordinated P–OH component was not required to fit the data and is below the limit of quantification; this is shown in the Supporting Information Tables S2 and S3. For those samples that have a quantifiable uncoordinated P–OH peak, an estimate of surface concentration is derived by converting the fractional intensity of this peak relative to the total O 1s peak intensity to an areal density based on an attenuation model and propagating the Monte Carlo uncertainty. These estimates are semiquantitative given the strong overlap of oxygen components in the O 1s peak area, but they offer useful insight for the net average binding mode on the surface, especially when compared to the molecular surface coverage, which will be discussed in more detail later.

Quantification of uncoordinated P–OH was done using eq 2. A derivation and explanation of this equation can be found in the Supporting Information Section S1.

$$\varphi_{P-OH}=f\cdot N_{O}\cdot L_{O1s}\cdot \cos\left(\theta \right)$$ 2

where φ_*P*–*OH*_ is the areal density of uncoordinated P–OH on the surface, *f* is the percentage contribution of the P–OH component peak to the overall O 1s peak, *N*_*O*_ is the stoichiometric density of oxygen per nm^3^ in the substrate, *L*_*O 1s*_ is the effective attenuation length of the O 1s photoelectrons within each substrate, and θ is the photoelectron takeoff angle. Only peaks with quantifiable intensity (based on our Monte Carlo analysis) are analyzed, and a summary of the results is shown in Table 1. Error bars are determined as the inverse variance weighted average of the Monte Carlo standard deviations within the fit for each peak area.

**Table 1:** P–OH Areal Densities for Each Molecule on ITO and α-Al_2_O_3_

Molecule	Φ_P–OH_ on ITO (P–OH groups/nm^2^)	Φ_P–OH_ on α-Al_2_O_3_ (P–OH groups/nm^2^)
2PACz	0.91 ± 0.90	1.35 ± 0.15
F-2PACz	0.66 ± 0.75	1.46 ± 0.17
Cl-2PACz	0.78 ± 0.89	0.81 ± 0.29
Br-2PACz	0.18 ± 0.15	0.78 ± 0.26
I-2PACz	0.79 ± 0.57	1.02 ± 0.28
^t^Bu-2PACz	0.98 ± 0.86	1.08 ± 0.34

These data are best understood in context of molecular surface coverage, found in Table 2, Figure 6, and in the Supporting Information Table S9. Each X-2PACz molecule in a fully unbound state possesses two uncoordinated P–OH groups. In context of molecular coverage, our XPS shows that each X-2PACz sample has between zero and one molecules of uncoordinated P–OH per molecule, implying on average one or more deprotonated P–OH groups which are bound to the metal oxide surface. Thus, the X-2PACz forms a bound monolayer on each sample, though exact information on distribution of binding modes cannot be determined by this analysis.

**Table 2:** X-ray Reflectivity Derived Morphology Metrics and Molecular Volumes for X-2PACz Variants on α-Al_2_O_3_

Molecule	X-2PACz layer Thickness (Å)	Surface Coverage (Molecules/nm^2^)	Molecular Volume (Å^3^)
2PACz	9.7	2.31 ± 0.38	259
F-2PACz	9.6	2.25 ± 0.28	269
Cl-2PACz	9.4	2.19 ± 0.28	293
Br-2PACz	9.1	1.80 ± 0.17	298
I-2PACz	9.1	1.61 ± 0.08	316
^t^Bu-2PACz	9.1	1.33 ± 0.40	411

Collectively, these results show that the oxygen species present demonstrate the formation of a bound monolayer on both ITO and alumina, evidenced by the increase in bound surface species after functionalization with a concentration of uncoordinated P–OH groups consistent with that of a deprotonated, bound monolayer. It should be noted that exact binding modes are difficult to assign using XPS. This determination leaves ambiguity on whether each molecule follows a homogeneous binding mode or whether a distribution of more or less deprotonated molecules exists, with fully deprotonated phosphonic acids mixed with protonated molecules that are less strongly hydrogen bonded to the surface, which some studies have documented.^44^ However, as we will discuss, the body of evidence supports the formation of a bound monolayer, with layer dimensions in the XRR consistent with a single molecular layer of each X-2PACz molecule.

### X-ray Reflectivity (XRR) Study of 2PACz on α-Al_2_O_3_

X-2PACz layers on α-Al_2_O_3_ were examined using XRR to gain further insight into the binding of the PA to the surface. X-2PACz and similar molecules are often assumed to form conformal, upright monolayers on metal oxides.^1–3,18,19,24^ However, a lack of experimental evidence of this morphology for 2PACz leaves conclusions about layer structure and packing speculative. XRR is a technique well suited for the study of surfaces, interfaces, and thin films. It provides information on interface roughness, layer thickness, and surface coverage as interpreted from an electron density profile.^45–48^ These are all properties of keen interest in the study of PA-surface modifiers and can help fill current knowledge gaps in X-2PACz morphology. Unfortunately, XRR is not amenable to substrates with high surface roughness, namely, commercially available ITO, which often has an RMS roughness of >1 nm. The ITO substrates employed in this study have an AFM measured RMS roughness of 3.6 nm (Supporting Information Figure S8). In a bonded state, a 2PACz molecule has a height of around 1 nm, meaning any bound monolayer of 2PACz on ITO measured with XRR produces a signal dominated by the ITO roughness. A comparison between the XRR from 2PACz functionalized ITO and bare ITO is shown in Figure S9, displaying nearly identical XRR profiles. While planarized ITO substrates would be advantageous for X-ray reflectivity measurements, they are not widely available and require postprocessing to achieve the low roughness necessary for reliable XRR measurements of thin films such as 2PACz.^49^ Therefore, we utilize a model system of single crystal α-Al_2_O_3_ (also referred to as C-plane sapphire) for XRR measurements of X-2PACz films. α-Al_2_O_3_ has a roughness less than 1 nm and is readily modified by phosphonic acids in a chemically similar manner to ITO.^12,50–52^ This allows for the study of X-2PACz layers with XRR by providing a well-defined and standard platform which minimizes the influence of substrate roughness while maintaining consistency in our deposition strategy.

XRR data of each X-2PACz layer on alumina were first modeled as single layers of uniform electron density. This treatment confirmed that layer thicknesses and electron densities for each molecule were consistent with the formation of a monolayer, with results found in the Supporting Information Table S4 and Figures S10, S11, and S12. With this insight, we then fit the data using a model that contained multiple sublayers with thicknesses of approximately 1 Å, representing the X-2PACz molecular structure. As a starting point, atomic positions of a vertical X-2PACz molecule (with respect to a substrate) were determined using energy minimized molecular models in the software IQmol (Supporting Information Section S5) and then binned into 1 Å thick sublayers. For each molecule, the ratios between the number of electrons in each sublayer and the whole molecule were determined. These electron ratios were then multiplied by the electron density of the single-layer XRR fit to provide initial estimates for the electron density in each sublayer. This generated a detailed electron density profile for model X-2PACz vertical standing molecules. Fitting these models to the experimental data consisted of simulating molecular tilt by applying a uniform reduction in each sublayer thickness. The total electron density of the layer, the proportionality of the sublayer electron densities, and the layer thickness were varied to achieve the best fit. This procedure is illustrated in Figure S13 and described more fully in the Supporting Information Section S2. The result is an excellent fit to the data with minimized FOM near 0.1 (fractional error of order ≈ 1%). The resulting electron density profile for each X-2PACz layer reveals a region of heightened electron density near the 2PACz tail, corresponding to the more electron dense carbazole unit. The constant position of this feature for each molecule confirms the consistent binding behavior and structure of each X-2PACz layer. Fitted and Fresnel normalized XRR traces for this granular model are depicted below in Figure 3a, with the accompanying electron density profile of 2PACz showing the interpreted layer structure. Additional electron density profiles for the remaining molecules are in the Supporting Information Figure S14.

**Figure 3:**
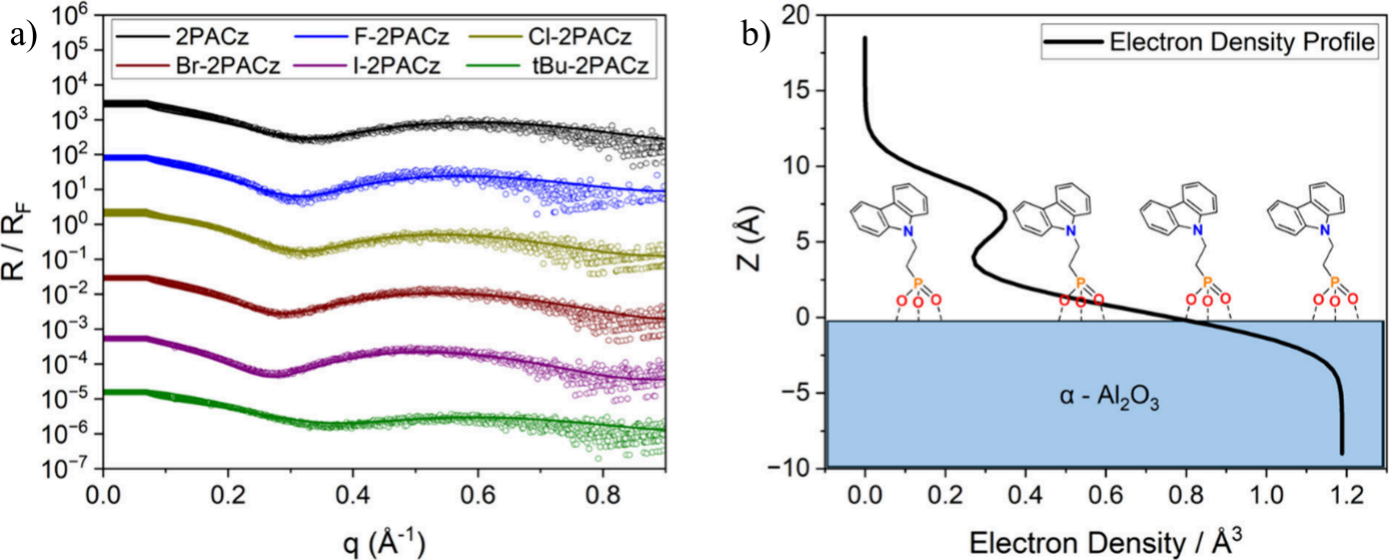
a) Fresnel-normalized XRR traces for each X-2PACz derivative on α-Al_2_O_3_ and b) the electron-density profile of 2PACz on α-Al_2_O_3_. Here R_F_ is the XRR from a flat α-Al_2_O_3_ substrate.

The length of an upright parent 2PACz molecule atop a metal oxide is approximately 10–11 Å, with each variant having a similar estimated length. However, the layer thickness, as measured by XRR, is influenced by the exact phosphonic acid binding mode, the conformation of the carbazole tail, and any molecular tilt. Surface coverage of each X-2PACz molecule on α-Al_2_O_3_ is derived using the electron density and overall layer thickness, with the procedure outlined in more detail in the Supporting Information Section S2. Estimates of maximum coverage of 2PACz and similar PAs on alumina, and other metal oxides, have been documented before, both on a basis of steric footprint and binding site availability.^12,31,52,53^ Here we estimate the sterically limited maximum molecular coverage. Recent studies have documented the π–π interaction distance for 2PACz of ∼4 Å.^54^ We estimate the width of a 2PACz tail group plus the expected van der Waals radii of the terminal hydrogens on either side, as ∼11Å (8.9 Å plus 1 Å for each hydrogen; see Supporting Information Table S5 and Figure S15). This generates a steric footprint estimation for a fully dense, rectangularly packed layer with a maximum surface coverage of 2.3 2PACz molecules per nm^2^. A depiction of these molecular dimensions and tiling of the substrate surface is shown in the Supporting Information Section S2. When limited by binding site availability on α-Al_2_O_3_, the maximum PA coverage is heavily impacted by the dominant PA binding mode, with maximum site-limited coverage ranging between 2.2 molecules per nm^2^ and 4.4 molecules per nm^2^ depending on whether the PA is in a predominantly tridentate or bidentate binding mode, respectivey.^12^

Results for the total layer thicknesses and surface coverages for each X-2PACz from XRR are depicted in Table 2, along with the steric bulk, represented by the molecular volume of each X-2PACz molecule and derived using the software MoloVol from the same IQmol molecular models.^55^ Having established that the primary species present on α-Al_2_O_3_ is the bound PA, for all the 2PACz variants studied, we conclude that the measured thicknesses of ca. (9 to 10) Å determined by fitting the XRR data are consistent with a monolayer of slightly tilted molecules. As we will discuss in the next section, this result is in good agreement with the NEXAFS derived tilts. Surface coverages of each X-2PACz layer are lower than the steric limitations of a dense layer for 2PACz (2.3 nm^–2^). Coverages range from 1.33 to 2.31 molecules per nm^2^, with a trend that coverage is inversely correlated to steric bulk of the tail group. Whether this effect emerges from steric effects on the substrate surface or kinetic effects during deposition is not clear. Furthermore, without specific binding mode information, it is unclear to what extent binding site saturation impacts the surface coverages. However, it is evident that, under the specified deposition conditions, near-monolayers of 2PACz and its derivatives are formed on α-Al_2_O_3_, and there is a concomitant reduction in surface coverage as the steric bulk of the tail group increases.

### Near-Edge X-ray Absorption Fine-Structure Spectroscopy (NEXAFS) for study of X-2PACz Molecular Orientation

NEXAFS at the carbon K-edge was used to better understand and compare the orientations of 2PACz bound to α-Al_2_O_3_ and ITO. Here, we employ a similar method for molecular orientation determination to that used by Gliboff et al. for phenylphosphonic acid.^10^ Figure 4 shows the NEXAFS TEY spectrum of 2PACz deposited on ITO. Figure 4a shows the C 1s absorption edge, with the C=C 1s to π* excitation highlighted in blue and expanded for clarity in Figure 4c. Other peaks in the figure are identified and consistent with the expected excitations from aromatic molecules.^38^ These other peaks may be used for molecular orientation determination, but the C=C C 1s to π* excitation is preferable because it is far more isolated from other excitations, making fitting much more accurate. TEY and PEY collection modes are particularly useful for molecularly thin layers, since they probe only the top few nanometers of the material due to the short escape depth of carbon photoelectrons of 1 to 3 nm.

**Figure 4:**
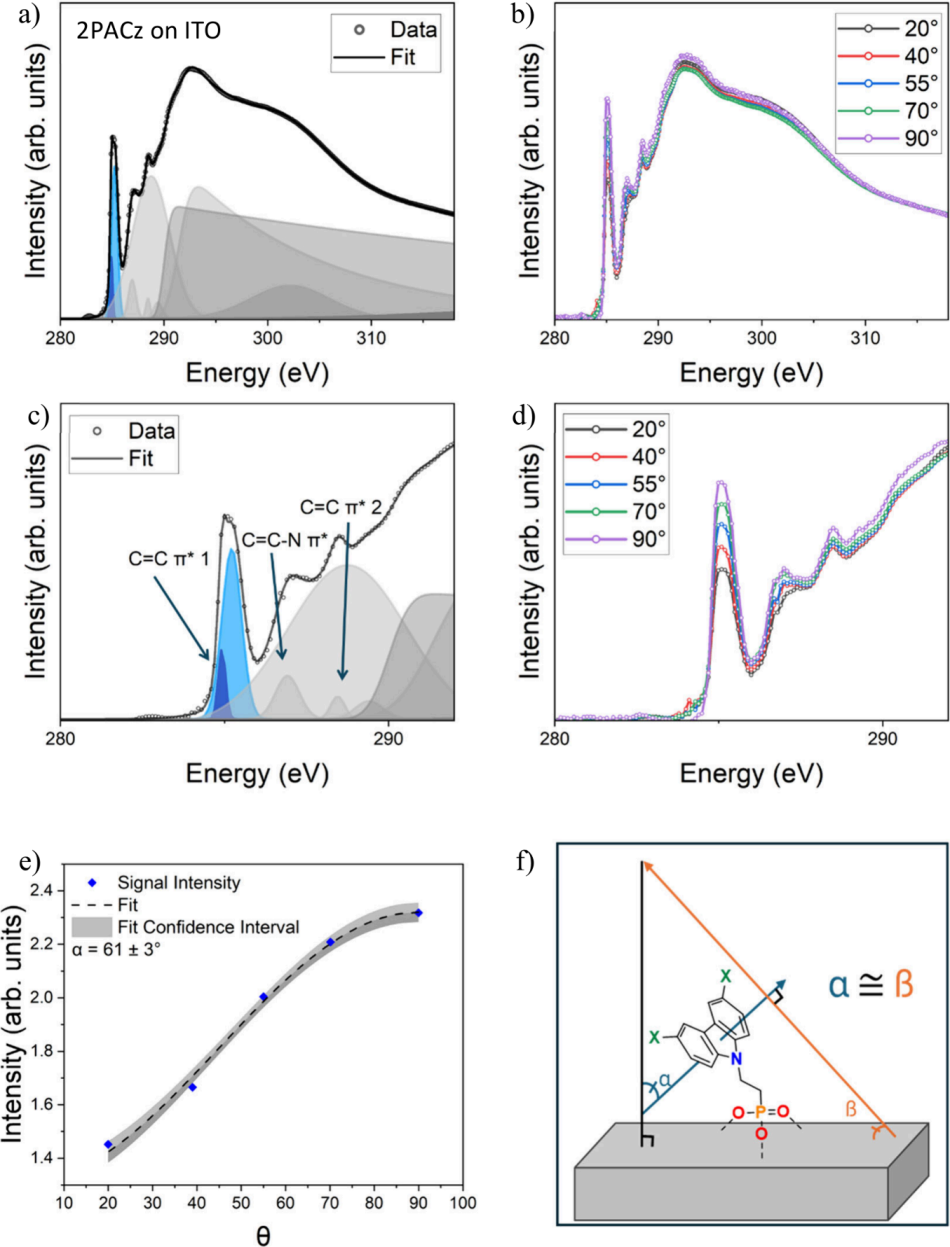
NEXAFS spectra of 2PACz on ITO showing a) a full scan with its associated peaks, b) scans of all 5 polarization angles, c) an expanded view of the pre-edge peak fit, d) an expanded view of the pre-edge data at all 5 angles, e) the fitted angular dependence of the C=C π* peak intensity used for tilt angle determination, and f) visualization of the tilt angle of 2PACz. The angle of the carbazole plane with respect to the substrate is shown to be congruent to the angle of the transition dipole moment with respect to the surface normal for a bound molecule. Peaks associated with the C=C π* excitation are highlighted in blue in (a) and (c).

Molecular orientation determination via carbon K-edge NEXAFS relies on the variation of the absorption probability of polarized X-rays with the angle between the polarization vector and the transition dipole moment of the C 1s to π* excitation, which in aromatic materials lies orthogonal to the plane of the ring.^38^

Higher X-ray absorption results when the incident X-ray polarization is parallel to the transition dipole, leading to intensity variations as a function of X-ray polarization angle.^10,38,39^ Figure 4b depicts NEXAFS of 2PACz on ITO taken at X-ray polarization angles of 90°, 70°, 55°, 40° and 20°, which are expanded for clarity in Figure 4d. This dependence of the C 1s to π* excitation intensity on the X-ray polarization angle shows a net preferred molecular orientation on ITO. An X-ray polarization of 0° is parallel to the substrate plane, while a polarization of 90° is orthogonal.

Spin coating of small molecule films like 2PACz produces radially symmetric deposition dynamics and has been shown to produce no preference in azimuthal orientation due to the absence of any force for directional alignment.^56^ Therefore, as in Gliboff et al., we assume no preferred in-plane orientation of the monolayer,^10^ and eq 3 is used which averages over the azimuthal orientation of the sample for average molecular angle determination.^38^

$$I_{v}\left(\varphi \right)=\frac{1}{3}\left[1+\frac{1}{2}\left(3\cos^{2}\theta -1\right)\left(3\cos^{2}\alpha -1\right)\right]$$ 3

Here, *I*_*v*_ is the intensity of the π* excitation, θ is the X-ray polarization angle with respect to the substrate, and α is the angle of the transition dipole of the π* excitation with respect to the surface normal (Figure 4f). Figure 4e shows the result of applying this model to the intensity variation to determine an average molecular tilt, with the result of α = 61° ± 3° for 2PACz on α-Al_2_O_3_.

It is important to mention that the angle α in eq 3 refers to the angle of the transition dipole moment of the orbital in question with respect to the surface normal (Figure 4f), and not, at least inherently, the angle of the carbazole plane of the molecule with respect to the substrate (β in Figure 4f). However, with the added context that the dominant species on either substrate is bound PA, α is congruent to β (the angle between the carbazole plane of the and the substrate), as depicted in Figure 4f. Therefore, the angles reported here are the angles between the aromatic plane of the carbazole and the plane of the substrate, given that the nature of the bound monolayer has been established. This methodology to determine molecular orientation has been applied to each 2PACz variant on both ITO and α-Al_2_O_3_. NEXAFS spectra and angular dependence fits for each additional molecule can be found in the Supporting Information Section S7. The average molecular tilts on both ITO and alumina are documented in Table 3.

**Table 3:** NEXAFS-Derived Average Molecular Tilts of the Carbazole Tail with respect to the Substrate Plane

Molecule	Average Tilt: ITO	Average Tilt Sapphire
2PACz	62° ± 3°	61° ± 3°
F-2PACz	62° ± 3°	64° ± 3°
Cl-2PACz	65° ± 3°	65° ± 3°
Br-2PACz	63° ± 3°	63° ± 3°
I-2PACz	64° ± 3°	65° ± 3°
tBu-2PACz	61° ± 3°	63° ± 3°

Our results show that the average tilt of the surface modifiers is identical, within experimental error, between each molecule, and the two different substrates. The experimental error of ±3° encompasses the expected variations in systematic factors such as angle calibration, beam polarization, and variations in normalization.^38,39^ Confidence intervals and residuals for these tilt angle fits are found in the Supporting Information Sections S3 and S7, respectively. The narrow confidence intervals, combined with the small residuals on tilt angle fits, indicate that a single average molecular orientation provides an adequate description of each sample. However, these confidence intervals do not provide information on the width of any underlying orientational distribution, and the presence of broader spatial heterogeneity cannot therefore be excluded. Having already established that films formed on α-Al_2_O_3_ are made up of bound molecules, with layer thicknesses and surface coverages representative of a single, sterically dense molecular layer, we conclude that this average molecular orientation is representative of that of a bound monolayer on the α-Al_2_O_3_ surface. The close agreement between average tilts taken between the two substrates for each molecule, coupled with the information from XPS (see above) that bound PA is also the predominant species on ITO, shows that a similar morphology of X-2PACz monolayers is formed on ITO. Confirmation of surface coverage on ITO using XPS was used to validate this hypothesis, incorporating morphological elements from XRR and NEXAFS to construct an informed surface coverage model (see below).

### Surface Coverage from X-ray Photoelectron Spectroscopy

Angle-resolved XPS-derived 2PACz coverage on ITO was determined by using a modification of an overlayer attenuation analysis following Lin et al.^1^ Our approach compares the ratios of XPS signals from indium and phosphorus, taken at several takeoff angles, and models the expected photoelectron attenuation through the overlayer to quantitatively determine the 2PACz coverage. This model relies upon estimates of several factors for accurate coverage determination, specifically the layer thickness of the X-2PACz layer and the effective attenuation length (EAL) for both phosphorus and indium photoelectrons through this layer. Note that the EAL is slightly different than electron inelastic mean free path (IMFP) and is appropriate to use in coverage determination.^57^ Since changes to the experimental takeoff angle are accounted for within our surface coverage model, there should be good agreement between the coverage estimates at all XPS takeoff angles.

Herein, the results from the XPS O 1s, XRR, and NEXAFS analyses are combined to provide an accurate quantification of surface coverage. As discussed, conclusions of the O 1s XPS analysis, in conjunction with the layer thickness from XRR, support the formation of a monolayer for each X-2PACz molecule with the phosphorus preferentially bound via oxygen to the substrate. This binding motif implies that the XPS signal is predominantly attenuated by the X-2PACz tail group. XRR results of X-2PACz on α-Al_2_O_3_ are used to estimate the layer thickness and density for X-2PACz on ITO and are needed for the EAL determination in our coverage model. While layer density for each molecule may differ between the two substrates, the density results from XRR are used for the estimation of EALs. As discussed further in the Supporting Information Section S4, differences in material density for EAL estimation do not have a substantive impact on the final coverage calculation. The XRR derived density, therefore, provides a reasonable estimation for EAL determination.

NEXAFS-derived average molecular orientation and the uncertainty in tail group rotation are incorporated to calculate accurate photoelectron attenuation for each X-2PACz. While NEXAFS provides the average orientation of each molecule, it does not provide insight into the rotation of the 2PACz tail group. This is illustrated in Figure 5, highlighting how 2PACz may possess different tail group rotations, while still producing identical NEXAFS signals since the orientation of the transition dipole of the carbazole π* orbitals with respect to the surface normal are the same. As described in Lin et al., when in a monolayer configuration, the attenuation of the phosphorus photoelectrons results from the carbazole tail group.^1^ Differences in tail group orientations therefore create differences in the layer thickness attenuating the phosphorus photoelectrons. The thickness differences between the shortest and longest lengths for the different extremes of tail group rotation are depicted in Figure 5 using tilt angles from NEXAFS. These yield a range of tail group thicknesses from 5.15 Å to 7.84 Å for 2PACz, with the thicknesses of the other 2PACz-X noted in the Supporting Information Section S4. Because NEXAFS provides no insight into rotation along this axis, the tail thickness values used in determination of the coverages are the average between the two extremes from either configuration. The subsequent coverages reported are the inverse variance weighted average from each acquisition angle using this average tail group thickness. Details on these calculations and EALs used for this analysis are in the Supporting Information Section S4. Table S9 and Figures 6 and S16 document the calculated surface coverages for each molecule. XPS spectra for the indium and phosphorus peaks used in this analysis may be found in the Supporting Information, Section S8.

**Figure 5:**
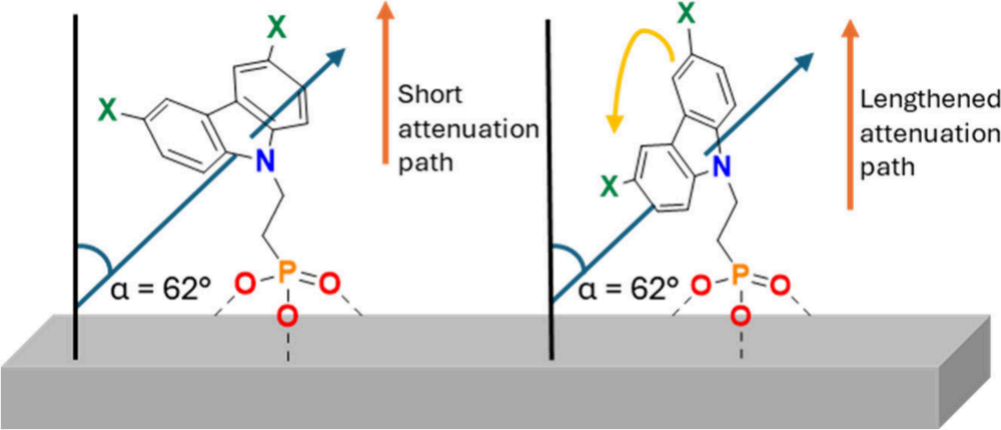
Variation in tail group rotation and impact on attenuation path.

**Figure 6:**
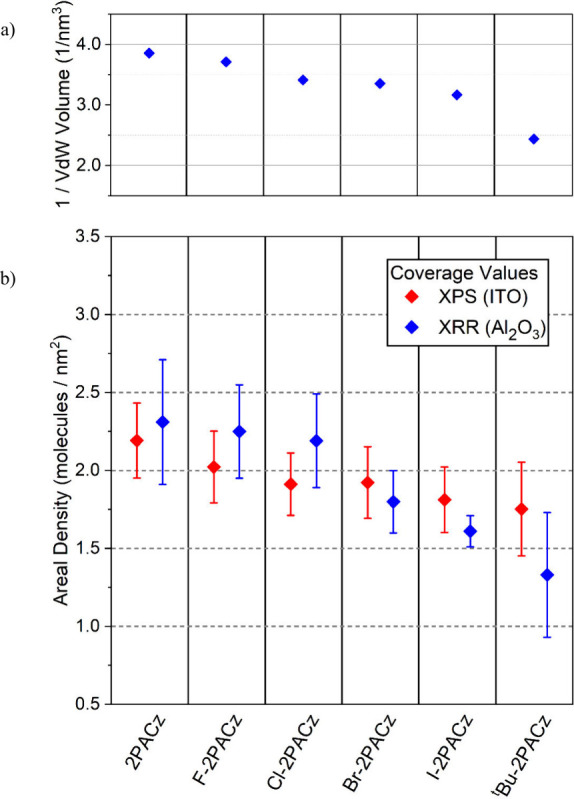
a) Steric bulk of X-2PACz molecules and b) angle-resolved XPS derived coverage on ITO (red) and compared with XRR derived coverages on α-Al_2_O_3_ (blue).

In Figure 6, we see a trend in coverage of X-2PACz on ITO with the inverse of the molecular steric bulk of each molecule. This decreasing coverage is consistent with the XRR results on α-Al_2_O_3_. While surface coverage is governed by the molecular footprint, and not the steric bulk, these quantities are closely correlated. The van der Waals volume used here is an orientation-independent parameter that is expected to grow monotonically with the 2D projection given the similar substitution mode on each X-2PACz molecule. The coverage values in Figure 6 represent the average of each XPS scan taken at takeoff angles of 0°, 40° and 55°. Scans at 70° were also attained, however coverage estimates for these scans are inaccurate due to shadowing by the ITO surface, which is elaborated on in the Supporting Information Section S4. Coverage estimates for scans at takeoff angles of 0°, 40° and 55° are all in close agreement, supporting the accurate determination of layer thicknesses and attenuation lengths. Error bar estimates are derived from a combination of uncertainty in the XPS fitted intensity and layer thicknesses (from XRR and NEXAFS) used in the XPS coverage model. Full descriptions of the errors associated with these variables can be found the Supporting Information Section S4.

This coverage estimate for 2PACz on ITO is close to the theoretical maximum coverage of 2.3 molecules per nm^2^ previously discussed. This is consistent with the conclusion that 2PACz surface coverage on ITO is sterically limited as opposed to site limited, and therefore the maximum coverage is dictated by the steric bulk of the surface modifier. Moreover, this trend is mirrored on ITO and α-Al_2_O_3_, and given the similarities in coverage and molecular orientation, it is reasonable to conclude this steric limitation is similar between the two substrates. Many of the samples measured here, namely those which are sterically bulkier than 2PACz (Cl-, Br-, I-, and ^t^Bu-2PACz), fall below the site-limited surface coverage limit calculated for α-Al_2_O_3_ of 2.2 molecules per nm^2^.^12^

For bulky surface modifiers like 2PACz, this finding suggests a plausible impact in degradation mechanisms within photovoltaic devices. That is, even in a fully saturated monolayer, this steric limitation may leave some potential reactive surface sites—such as acidic OH groups or undercoordinated metal ions—uninvolved in bonding to the PAs. On the other hand, the steric protection offered by the bulky tails might still mitigate the impact of these sites on device performance and/or stability. Complete isolation of substrate effects therefore may require multilayer architectures, mixed surface modifiers, or complementary passivation strategies, several of which have been demonstrated already.^15,24,33,34,58^ Exposed residual defects in the TCO are known to act as recombination centers and contribute to nonradiative losses. In contrast, dense SAM packing has been shown to improve interfacial passivation, enhance charge extraction, and increase Voc and Fill Factor in thin film photovoltaics.^15,59,60^ Further verification of the efficacy of these approaches in either more fully passivating or better isolating the TCO interface via multilayer architectures would be insightful future directions for work to engineer better, more stable SAM-HTLs.

To assess the similarity between the SAM layers studied here and those documented in the literature, work-function measurements were carried out via ultraviolet photoelectron spectroscopy (UPS) and are in close agreement with established literature values (see Figure S18 and Table S17). This reinforces the monolayer nature of 2PACz deposited in the other studies.

## Conclusions

We have demonstrated that each of the X-2PACz derivatives forms a monolayer using common spin coating deposition. This is evidenced by the concentration of uncoordinated P–OH groups between 0 and 1 per molecule, showing a majority deprotonated, bound state of the PA, supported by the conclusions of XRR-determined layer thicknesses consistent with single molecular layers. When comparing monolayers of X-2PACz on ITO and α-Al_2_O_3_, we also show that both the average molecular orientation and surface packing density of each molecule are the same for the two substrates. Deposited under identical conditions, this close agreement between surface coverages, average molecular orientations, and the correlation between surface coverage and steric bulk supports the hypothesis that bulky PA surface modifiers, like 2PACz, are sterically limited in their monolayer surface packing density. This steric limitation implies that a saturated monolayer of bulky molecules like 2PACz leaves potentially reactive surface sites on the substrate which are not active in PA bonding. As the number of materials studied for transport layers grows, our results support the viability of using model systems like α-Al_2_O_3_, and complementary techniques such as XRR, to better understand trends in packing density of molecules like 2PACz on ITO under comparable processing conditions. There remain inherent differences in the surface environments of both substrates, but we have demonstrated here that X-2PACz monolayers are produced on both substrates with quantitatively similar binding behavior, molecular tilt, and surface coverage. While we have shown that these techniques can be highly effective in 2PACz characterization, future work should investigate the growing field of increasingly effective PA modifiers and codeposited surface modification strategies to connect gains in performance and stability to surface modifier layer structure and packing density.

## Supplementary Material



## Data Availability

All data are available in the main text or the Supporting Information.

## Notes

Supplementary experimental methods, XPS peak fitting procedure and spectra, X-ray reflectivity models, fits, and coverage analysis, NEXAFS spectra, angular fits, and residuals, UPS work function measurements, molecular models, ITO AFM scan, and additional tables of structural and analytical parameters (PDF)
