# Supplementary material for: Structure of Layers Formed by [2-(3,6-Disubstituted‑9H‑carbazol-9-yl)ethyl]phosphonic Acids on Metal Oxides
Source: ACS Appl Mater Interfaces. 2026 May 17;18(20):29278–90. doi: 10.1021/acsami.6c03387 (PMC13220220; doi:10.1021/acsami.6c03387)
Supplement: Supplementary file 1 [file am6c03387_si_001.pdf]

Supporting Information

Structure of Layers Formed by [2-(3,6-Disubstituted-9*H*-carbazol-9-yl)ethyl]phosphonic Acids on Metal Oxides

Collin Sindt[1], Oliver Wright[2,3], Yadong Zhang[4], Junxiang Zhang[4], Patteera Funchien[4], Lars Thomsen[5], Eliot Gann[6], Cherno Jaye[7], Stephen Barlow[4], Seth R. Marder*[1,3,4,8], Michael F. Toney*[1,2,4,8]

* Corresponding authors: Michael.Toney@colorado.edu; seth.marder@colorado.edu

[1]Department of Chemical and Biological Engineering, University of Colorado Boulder, Boulder CO 80303, USA
[2]Department of Physics, University of Colorado Boulder, Boulder CO 80303, USA
[3]Department of Chemistry, University of Colorado Boulder, Boulder CO 80303, USA
[4]Renewable and Sustainable Energy Institute, University of Colorado Boulder, Boulder CO 80303, USA
[5]Australian Synchrotron, ANSTO, 800 Blackburn Road, Clayton, Victoria, 3168, Australia
[6]National Synchrotron Light Source II, Brookhaven National Laboratory, Upton, New York 11973, USA
[7]Materials Measurement Science Division, National Institute of Standards and Technology, Gaithersburg, MD, 20899 USA
[8]Materials Science Program, University of Colorado Boulder, Boulder CO 80303, USA

Table of Contents

Table of Contents 2

Table of Figures 4

Table of Tables 8

S1. O1s XPS Analysis 9

Uncoordinated P-OH Surface Concentration Determination 16

S2. X-Ray Reflectivity 21

XRR Fitting Technique 23

Surface Coverage Determination from X-ray Reflectivity 35

X-ray Reflectivity Error Bar Determination 35

Estimation of Sterically Limited Coverage 36

S3. NEXAFS Spectroscopy Analysis 37

S4. XPS analysis of SAM Coverage 40

XPS Fitting Parameters 51

S5. Molecular Models of X-2PACz Molecules 53

S6. UPS Work Function Measurements 61

S7. NEXAFS Spectra and Angular Dependent Fits 62

Spectra and Tilt Angle Fits 62

Tilt Angle Fit Residuals 68

S8. In 3d and P 2p XPS Spectra 74

References 76

Table of Figures

Figure S1: XPS scan of the O1s region of 2PACz powder 9

Figure S2: O1s XPS spectra acquired at takeoff angles of 0° and 70° for a) 2PACz, b) F-2PACz, c) Cl-2PACz, d) Br-2PACz, e) I-2PACz, and f) t-Bu-2PACz on ITO 10

Figure S3: Lattice oxygen contribution to the overall O1s signal as a function of takeoff angle for a) 2PACz, b) F-2PACz, c) Cl-2PACz, d) Br-2PACz, e) I-2PACz, and f) ^{t}Bu-2PACz on ITO. 11

Figure S4: Surface oxygen species (In-OH, In-O-P, P=O, P-OH) contribution to the overall O1s signal as a function of takeoff angle for a) 2PACz, b) F-2PACz, c) Cl-2PACz, d) Br-2PACz, e) I-2PACz, and f) ^{t}Bu-2PACz on ITO. 12

Figure S5:O1s XPS spectra acquired at takeoff angles of 0° and 70° for a) 2PACz, b) F-2PACz, c) Cl-2PACz, d) Br-2PACz, e) I-2PACz, and f) ^{t}Bu-2PACz on α-Al2O3. 13

Figure S6: Lattice oxygen contribution to the overall O1s signal as a function of takeoff angle for a) 2PACz, b) F-2PACz, c) Cl-2PACz, d) Br-2PACz, e) I-2PACz, and f) ^{t}Bu-2PACz on α-Al2O3. 14

Figure S7:Surface oxygen species (Al-OH, Al-O-P, P=O, P-OH) contribution to the overall O1s signal as a function of takeoff angle for a) 2PACz, b) F-2PACz, c) Cl-2PACz, d) Br-2PACz, e) I-2PACz, and f) ^{t}Bu-2PACz on α-Al_2O_3 15

Figure S8: AMF and RMS Roughness of Pristine ITO... 21

Figure S9: XRR plot of pristine ITO vs 2PACz modified ITO. 22

Figure S10:1-layer electron Density Profile of 2PACz on α-Al2O3.. 23

Figure S11: Single Layer XRR Electron Density Profiles of each X-2PACz. 24

Figure S12:Single-Layer Fit Reduced Reflectivity Data and Fits for X-2PACz on α-Al_2O_3 25

Figure S13: Granular 2PACz model, showing how the tilt of the molecule manifests as smaller individual sub-layers. 27

Figure S14: Multi-layer, granular electron density profiles of a) 2PACz, b) F-2PACz, c) Cl-2PACz, d) Br-2PACz, ... 34

Figure S15: Depiction of 2PACz VdW volume, and estimation of sterically limited packing density, assuming rectangular molecular packing. 36

Figure S16: Angle resolved XPS coverage estimations 50

Figure S17: IQmol Models of a) 2PACz, b) F-2PACz, c) Cl-2PACz, d) Br-2PACZ, e)I-2PACz, and f) t-Bu-2PACz. 53

Figure S18: UPS Secondary Electron Cutoff Positions of 2PACz materials on ITO 61

Figure S19: a) NEXAFS spectrum and fit and b) tilt angle fit for 2PACz on ITO, acquired at ANSTO-SXR 62

Figure S20: a) NEXAFS spectrum and fit and b) tilt angle fit for 2PACz on ITO, acquired at NSLS-II 7-ID-1 62

Figure S21: a) NEXAFS spectrum and fit and b) tilt angle fit for 2PACz on α-Al_2O_3, acquired at ANSTO-SXR 62

Figure S22: a) NEXAFS spectrum and fit and b) tilt angle fit for F-2PACz on ITO, acquired at ANSTO-SXR 63

Figure S23: a) NEXAFS spectrum and fit and b) tilt angle fit for F-2PACz on ITO, acquired at NSLS-II, 7-ID-1 63

Figure S24: a) NEXAFS spectrum and fit and b) tilt angle fit for F-2PACz on α-Al_2O_3, acquired at NSLS-II, 7-ID-1 63

Figure S25: a) NEXAFS spectrum and fit and b) tilt angle fit for Cl-2PACz on ITO, acquired at ANSTO-SXR 64

Figure S26: a) NEXAFS spectrum and fit and b) tilt angle fit for Cl-2PCz on ITO, acquired at NSLS-II, 7-ID-1 64

Figure S27: a) NEXAFS spectrum and fit and b) tilt angle fit for Cl-2PACz on α-Al_2O_3, acquired at ANSTO-SXR 64

Figure S28: a) NEXAFS spectrum and fit and b) tilt angle fit for Br-2PACz on ITO, acquired at ANSTO-SXR 65

Figure S29: a) NEXAFS spectrum and fit and b) tilt angle fit for Br-2PACz on ITO, acquired at NSLS-II, 7-ID-1 65

Figure S30: a) NEXAFS spectrum and fit and b) tilt angle fit for Br-2PACz on α-Al_2O_3, acquired at ANSTO-SXR 65

Figure S31: a) NEXAFS spectrum and fit and b) tilt angle fit for I-2PACz on ITO, acquired at ANSTO-SXR 66

Figure S32: a) NEXAFS spectrum and fit and b) tilt angle fit for I-2PACz on ITO, acquired at NSLS-II, 7-ID-1 66

Figure S33: a) NEXAFS spectrum and fit and b) tilt angle fit for I-2PACz on α-Al_2O_3, acquired at ANSTO-SXR 66

Figure S34: a) NEXAFS spectrum and fit and b) tilt angle fit for ^{t}Bu-2PACz on ITO, acquired at ANSTO-SXR 67

Figure S35: a) NEXAFS spectrum and fit and b) tilt angle fit for ^{t}Bu-2PACz on ITO, acquired at NSLS-II, 7-ID-1 67

Figure S36: a) NEXAFS spectrum and fit and b) tilt angle fit for ^{t}Bu-2PACz on α-Al_2O_3, acquired at ANSTO-SXR 67

Figure S37: Tilt Angle Fit Residual of 2PACz on ITO Acquired at ANSTO-SXR 68

Figure S38: Tilt Angle Fit Residual of 2PACz on ITO Acquired at NSLS-II, 7-ID-1 68

Figure S39: Tilt Angle Fit Residual of 2PACz on α-Al_2O_3 Acquired at ANSTO-SXR 68

Figure S40: Tilt Angle Fit Residual of F-2PACz on ITO, Acquired at ANSTO-SXR 69

Figure S41: Tilt Angle Fit Residual of F-2PACz on ITO, Acquired at NSLS-II, 7-ID-1 69

Figure S42: Tilt Angle Fit Residual of F-2PACz on α-Al_2O_3, Acquired at NSLS-II, 7-ID-1 69

Figure S43: Tilt Angle Fit Residual of Cl-2PACz on ITO, Acquired at ANSTO-SXR 70

Figure S44: Tilt Angle Fit Residual of Cl-2PACz on ITO, Acquired at NSLS-II, 7-ID-1 70

Figure S45: Tilt Angle Fit Residual of Cl-2PACz on α-Al_2O_3, Acquired at ANSTO-SXR 70

Figure S46: Tilt Angle Fit Residual of Br-2PACz on ITO, Acquired at ANSTO-SXR 71

Figure S47: Tilt Angle Fit Residual of Br-2PACz on ITO, Acquired at NSLS-II, 7-ID-1 71

Figure S48: Tilt Angle Fit Residual of Br-2PACz on α-Al_2O_3, Acquired at ANSTO-SXR 71

Figure S49: Tilt Angle Fit Residual of I-2PACz on ITO, Acquired at ANSTO-SXR 72

Figure S50: Tilt Angle Fit Residual of I-2PACz on ITO, Acquired at NSLS-II, 7-ID-1 72

Figure S51: Tilt Angle Fit Residual of I-2PACz on α-Al_2O_3, Acquired at ANSTO-SXR 72

Figure S52: Tilt Angle Fit Residual of ^{t}Bu-2PACz on ITO, Acquired at ANSTO-SXR 73

Figure S53: Tilt Angle Fit Residual of ^{t}Bu-2PACz on ITO, Acquired at NSLS-II, 7-ID-1 73

Figure S54: Tilt Angle Fit Residual of ^{t}Bu-2PACz on α-Al_2O_3, Acquired at ANSTO-SXR 73

Table of Tables

Table S1: Ratios between surface and bulk oxygen intensities for O1s XPS Spectra 17

Table S2: O1s Uncoordinated P-OH Peak Percentages and Monte Carlo Standard Deviations - ITO, with quantifiable levels of uncoordinated P-OH in green and unquantifiable levels in orange 19

Table S3: O1s Uncoordinated P-OH Peak Percentages and Monte Carlo Standard Deviations - α-Al_2O_3 20

Table S4: Single-Layer Fit Results for XRR of X-2PACz on α-Al2O3 25

Table S5: Granular X-2PACz XRR Fits 28

Table S6: NEXAFS Tilts and 95% Confidence Intervalsof Tilt Angle Fit for X-2PACz on α-Al_2O_3 39

Table S7: NEXAFS Tilts and Standard Deviations of Tilt Angle Fit for X-2PACz on ITO, Separated by Synchrotron Used 39

Table S8: X-2PACz Tail Group Dimensions 43

Table S9: EALs for In 3d and P 2p photoelectrons through X-2PACz 44

Table S10: X-2PACz Solution Derived Band Gaps 45

Table S11: Weighted Average Coverages for X-2PACz on ITO 46

Table S12: Error Analysis Example for Uncertainties in XPS Coverage Analysis...................... 47

Table S13: Uncertainties in Coverage Calculation from XPS for Each X-2PACz Sample 48

Table S14: O1s XPS Fitting Parameters.. 51

Table S15: In and P XPS fitting parameters.. 52

Table S16: XYZ Files of 2PACz Models 54

Table S17: Measured and reported work functions for 2PACz modified ITO 61

S1. O1s XPS Analysis

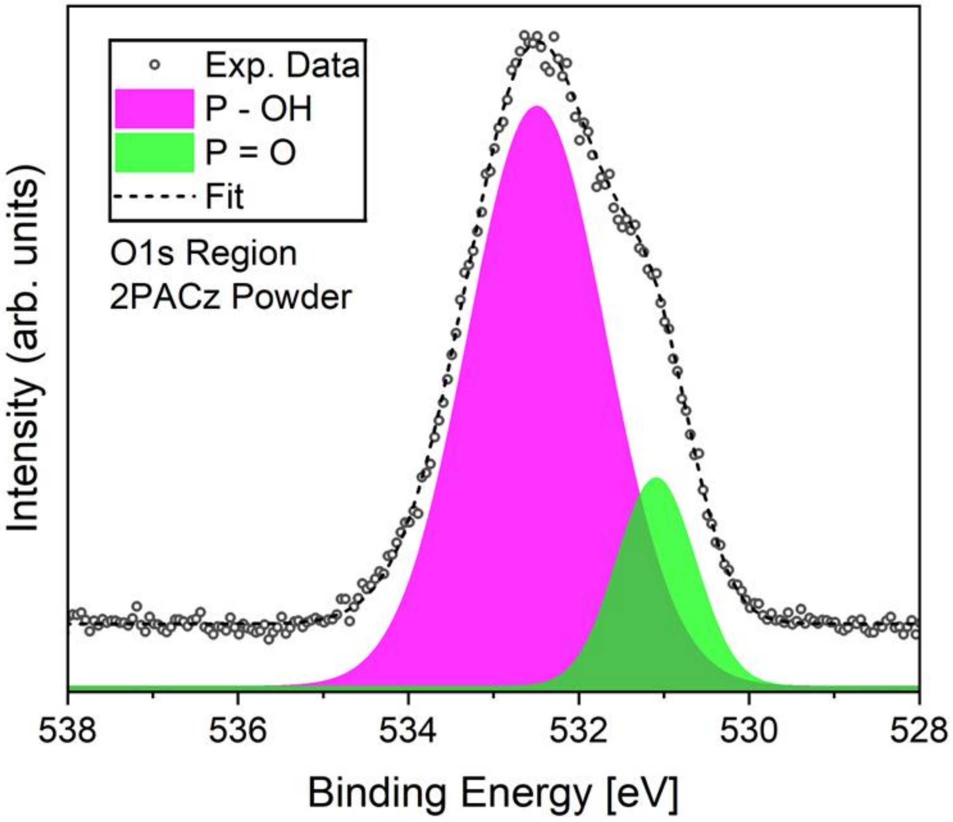

Figure S1: *XPS scan of the O1s region of 2PACz powder*

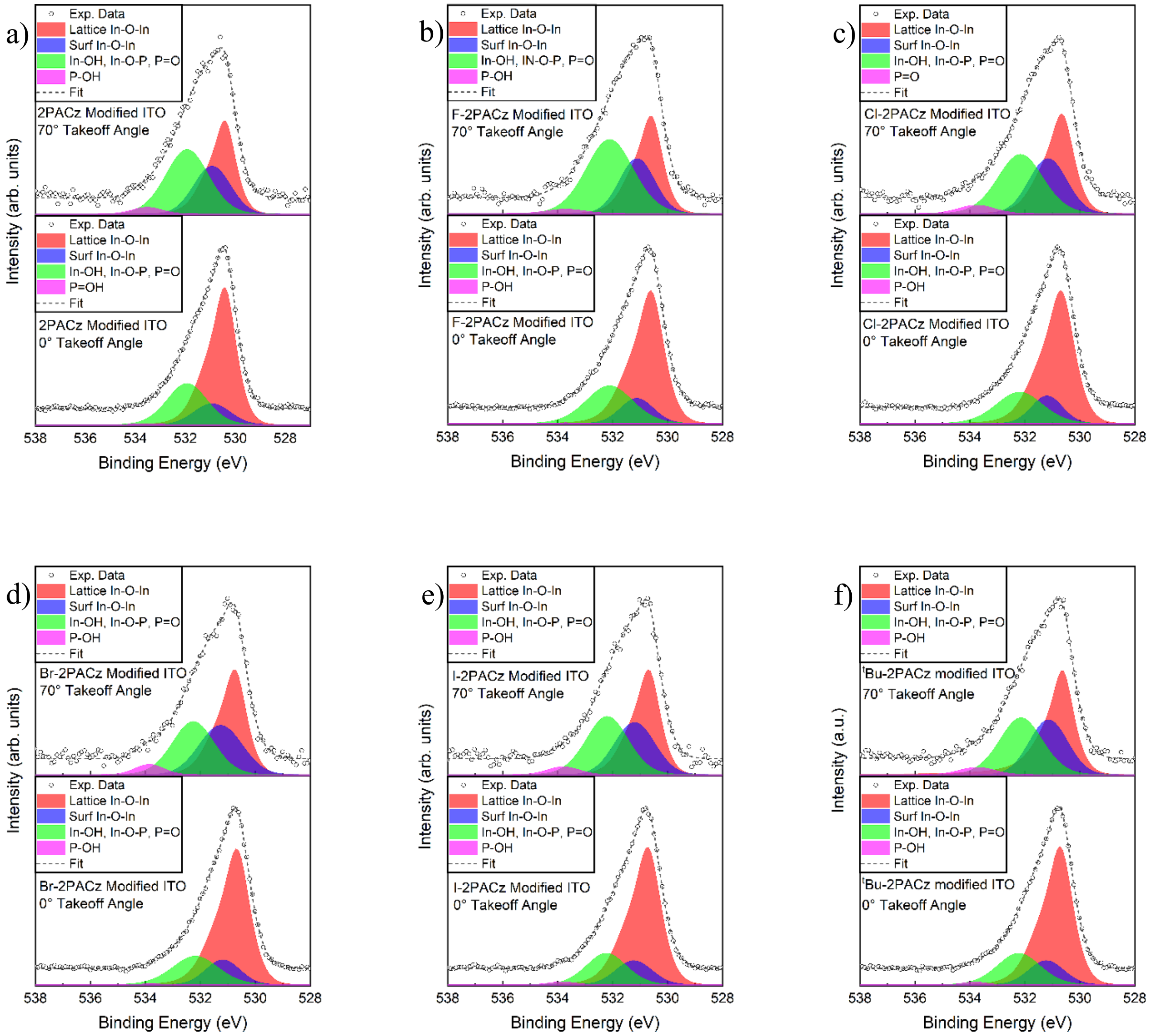

Figure S2: *O1s XPS spectra acquired at takeoff angles of 0° and 70° for a) 2PACz, b) F-2PACz, c) Cl-2PACz, d) Br-2PACz, e) I-2PACz, and f) t-Bu-2PACz on ITO*

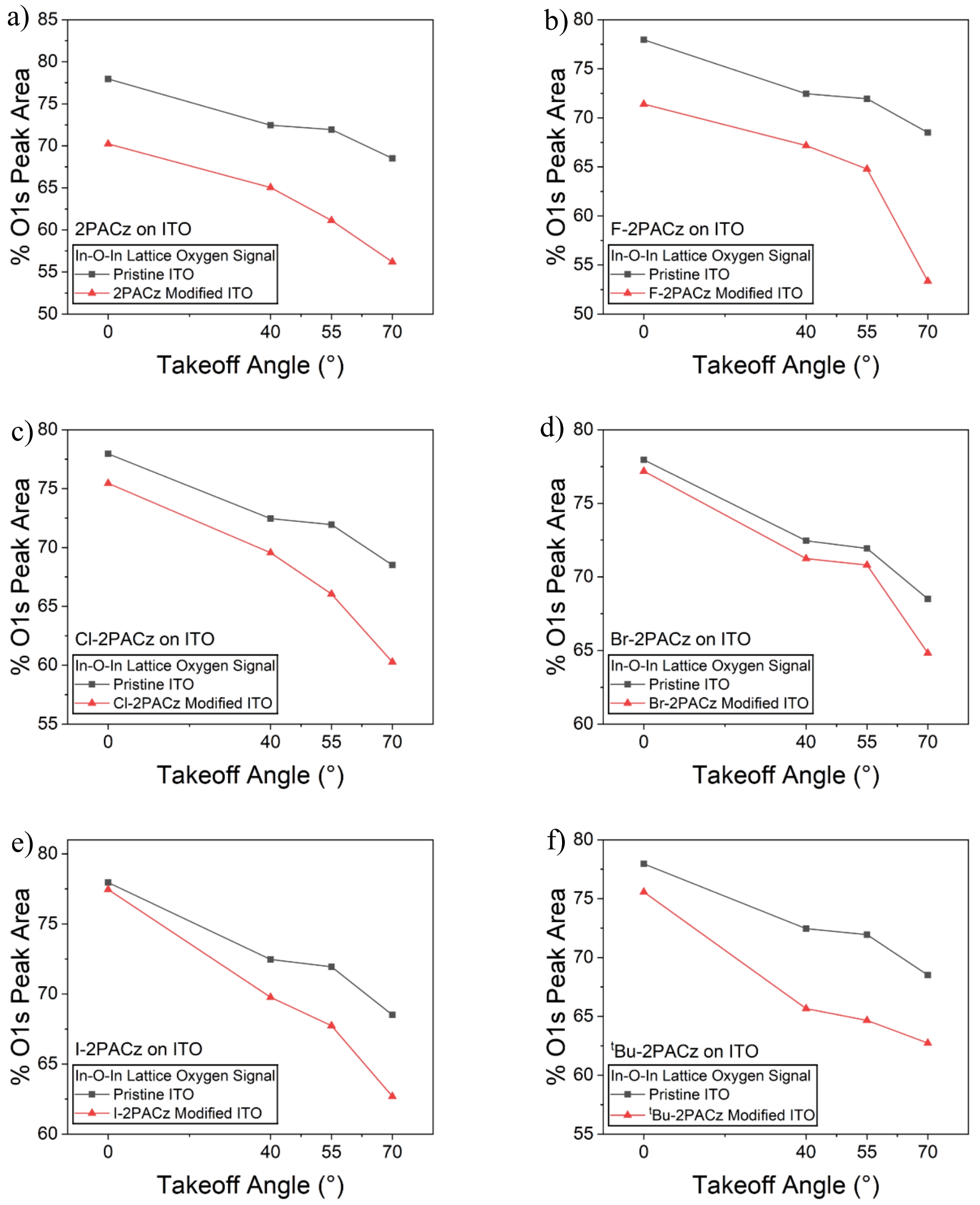

***Figure S3**: Lattice oxygen contribution to the overall O1s signal as a function of takeoff angle for a) 2PACz, b) F-2PACz, c) Cl-2PACz, d) Br-2PACz, e) I-2PACz, and f) ^{t}Bu-2PACz on ITO.*

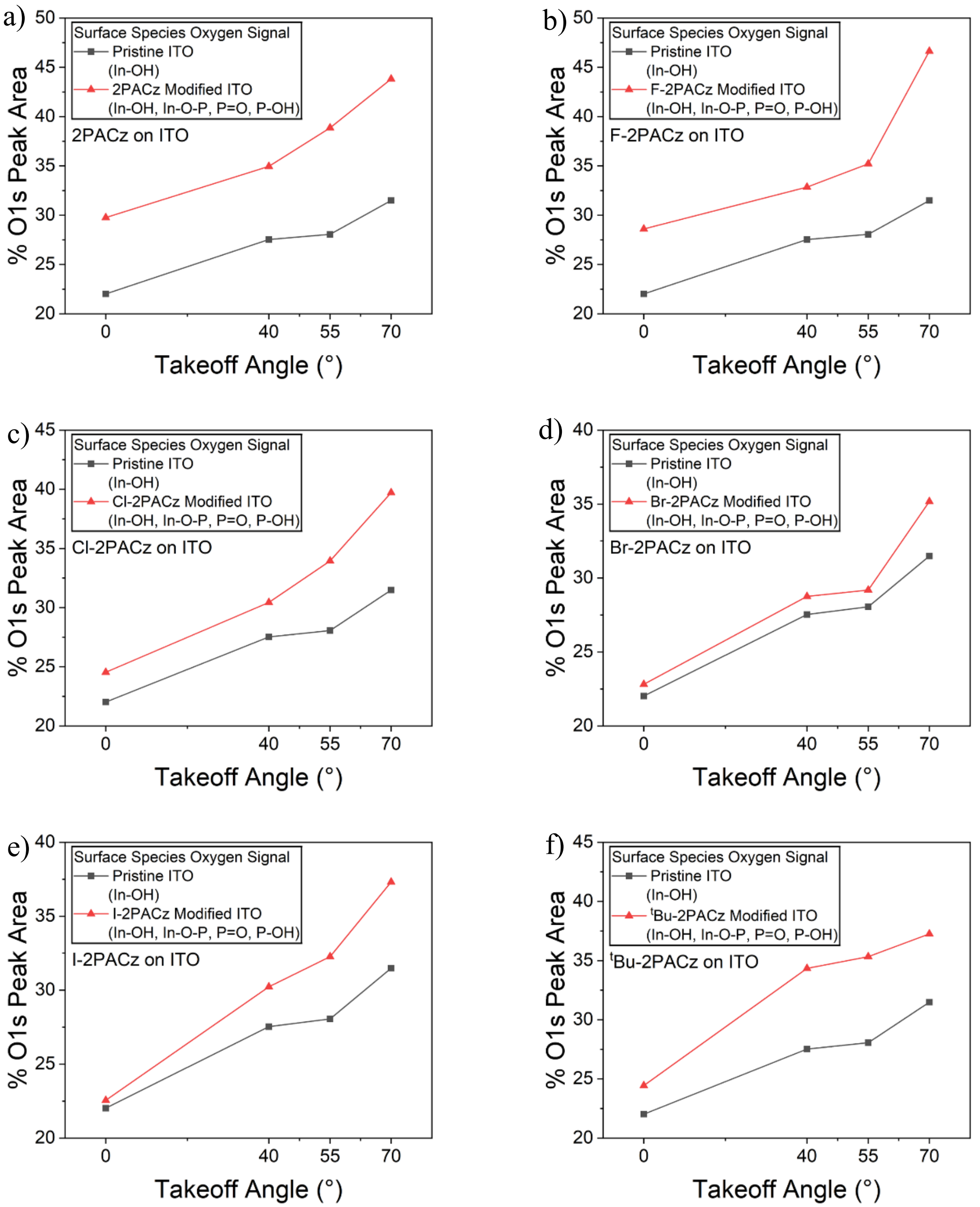

Figure S4: *Surface oxygen species (In-OH, In-O-P, P=O, P-OH) contribution to the overall O1s signal as a function of takeoff angle for a) 2PACz, b) F-2PACz, c) Cl-2PACz, d) Br-2PACz, e) I-2PACz, and f) ^{t}Bu-2PACz on ITO.*

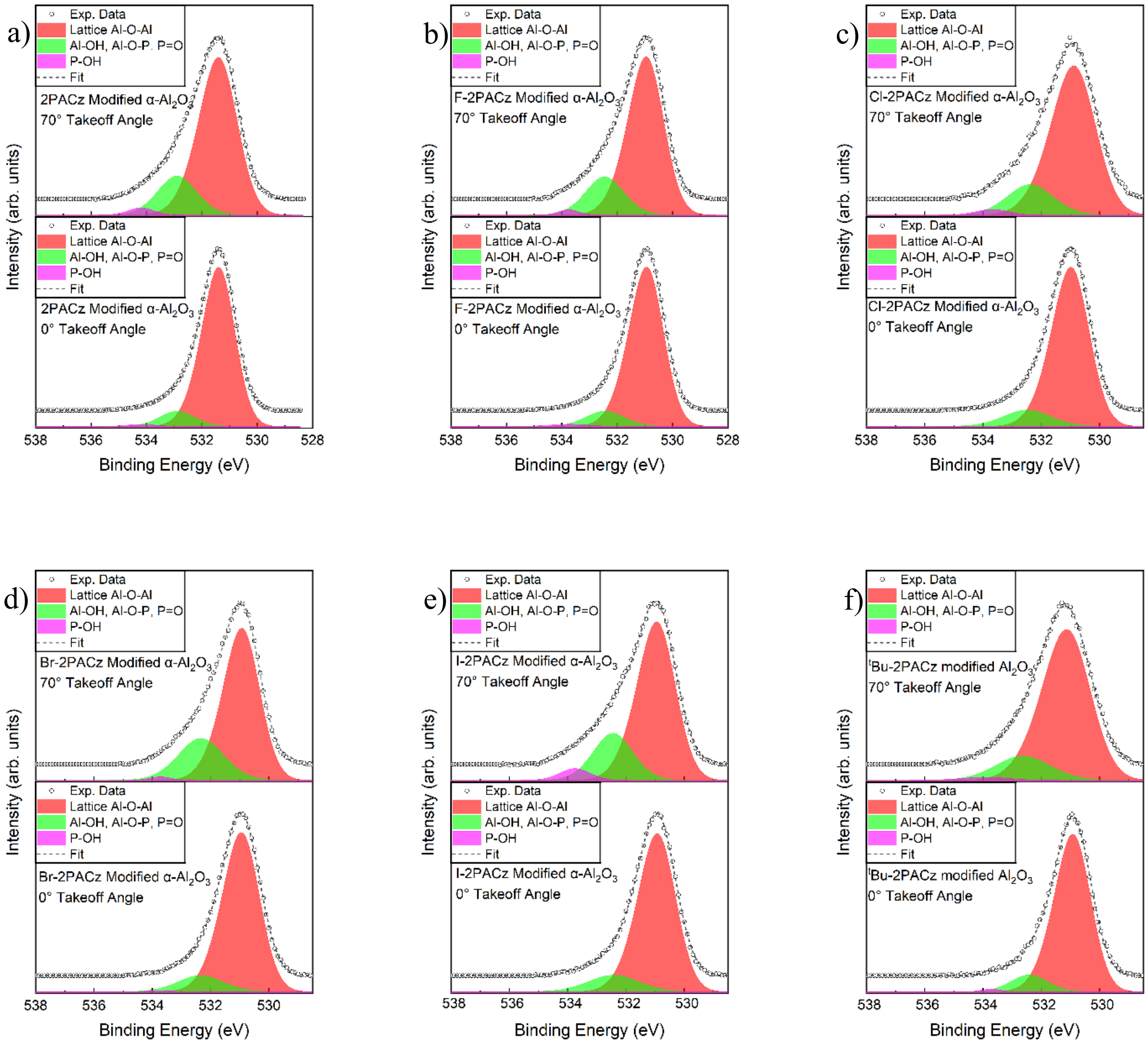

Figure S5:*O1s XPS spectra acquired at takeoff angles of 0° and 70° for a) 2PACz, b) F-2PACz, c) Cl-2PACz, d) Br-2PACz, e) I-2PACz, and f)* ^{t}*Bu-2PACz on α-Al2O3.*

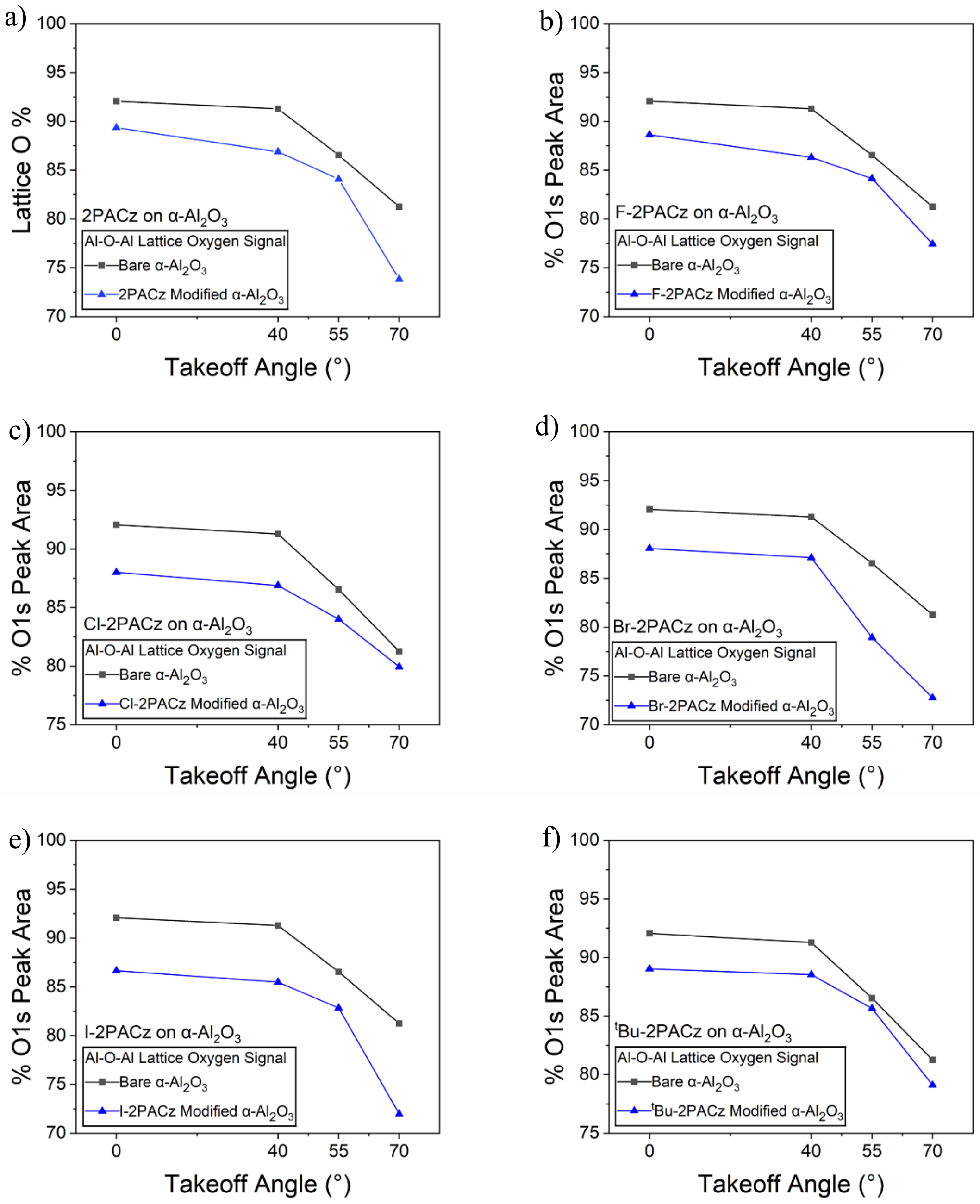

Figure S6*: Lattice oxygen contribution to the overall O1s signal as a function of takeoff angle for a) 2PACz, b) F-2PACz, c) Cl-2PACz, d) Br-2PACz, e) I-2PACz, and f) ^{t}Bu-2PACz on α-Al2O3.*

.

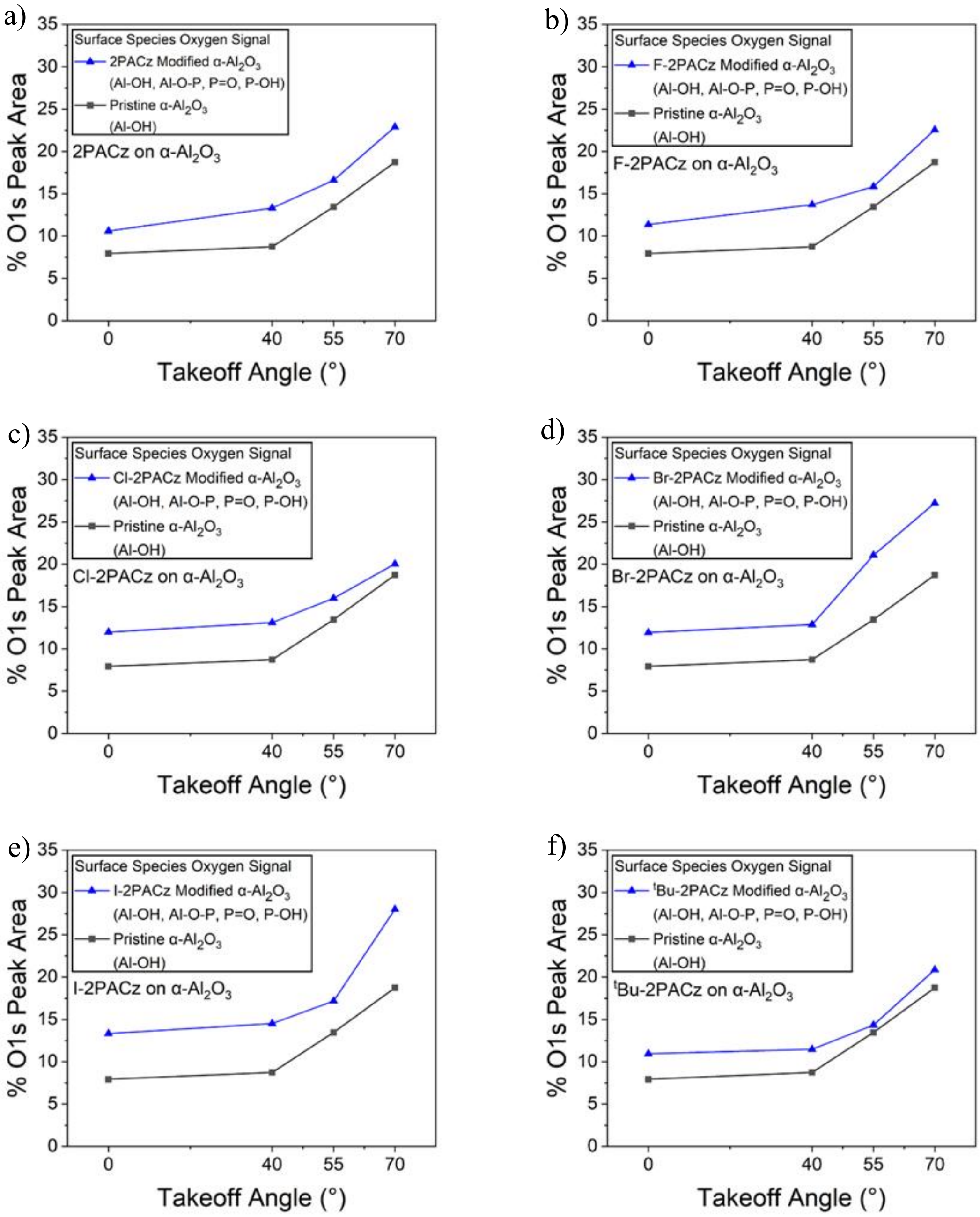

Figure S7*:Surface oxygen species (Al-OH, Al-O-P, P=O, P-OH) contribution to the overall O1s signal as a function of takeoff angle for a) 2PACz, b) F-2PACz, c) Cl-2PACz, d) Br-2PACz, e) I-2PACz, and f) ^{t}Bu-2PACz on α-Al_2O_3*

Uncoordinated P-OH Surface Concentration Determination

O1s XPS spectra were fit according to the peak models laid out in the main text, with peak shapes documented in section S4. For ITO, due to considerable peak overlap between the bulk lattice oxygens (530.6 eV) and surface oxygens (531.1 eV), a constraint was applied to the ratio of peak areas between these two signals based on an attenuation model. For the surface oxygen peaks, we model these as oxygens residing within the topmost layer of ITO. With an interplanar distance of 0.292 nm, the intensity from the top layer of oxygens in ITO is approximated as:

$$I_{Surf\ O} \propto N_O * \int_0^{0.292} e^{\left(\frac{-x}{L_{O1s}*\cos(\theta)}\right)} dx$$

Equation S1

where $I_{Surf,\ O}$ is the measured intensity within the O1s signal from surface oxygens, N_O is the concentration of oxygen in ITO, L_{O1s} is the effective attenuation length of O1s photoelectrons through ITO, estimated at 1.53 nm using the NIST EAL Database,[1] and θ is the takeoff angle. An analogous expression represents the signal from the bulk lattice, modeled as an infinite slab beneath the first layer:

$$I_{Bulk\ O} \propto N_O * \int_{0.292}^{\infty} e^{\left(\frac{-x}{L_{O1s}*\cos(\theta)}\right)} dx$$

Equation S2

The ratio of these terms is used as a constraint for the area of the surface oxygen peak relative to the area of the bulk oxygen peak in the overall O1s spectra for each sample on ITO. The computed ratios for each angle are shown in Table S1.

Table S1: *Ratios between surface and bulk oxygen intensities for O1s XPS Spectra*

Takeoff Angle	Surface/Bulk Intensity Ratio
0°	0.23
40°	0.31
55°	0.43
70°	0.82

When unconstrained, the peaks in the ITO O1s XPS spectrum are highly variable due to their strong overlap, which is especially true of the bulk and surface oxygen peaks due to the comparatively small binding energy shift between them. Constraining the peak area of the surface lattice oxygens compared to the bulk allows for the accurate determination of Monte Carlo error statistics for the surface oxygen species. This in turn is crucial for the calculation of uncoordinated P-OH concentration, whose peaks are comparatively weaker and more sensitive to variations in peak intensities. Data for the percentage of each O1s spectra made up of each uncoordinated P-OH peak, and its accompanying Monte Carlo standard deviation, are shown in Tables S2 and S3, along with whether the peak is quantifiable based on the Monte Carlo standard deviation, and the resulting inverse variance weighted average P-OH surface concentration and standard deviation.

The concentration of uncoordinated P-OH on the surface was determined using an overlayer attenuation model, similar to the one used to measure the surface concentration of X-2PACz molecules. The concentration of uncoordinated P-OH groups on the substrate surface in

comparison to the overall oxygen content in the information depth is small, as judged by the small contribution to the overall O1s signal. Therefore, the concentration of uncoordinated P-OH is estimated by treating the total O1s concentration in the information depth as that of the metal oxide lattice and ignoring the uncoordinated P-OH signal in this total. The signal from the oxygens in the substrate can be approximated in an identical manner to the attenuation analysis above used to constrain the O1s peak set for ITO. The total oxygen signal from the substrate is then modeled as:

$$I_{O1s} \propto N_O * \int_0^{\infty} e^{\left(\frac{-x}{L_{O1s}*\cos(\theta)}\right)} dx = N_O * L_{O1s} * \cos(\theta)$$

Equation S3

with variables identical to those described previously, but in this case relevant to both α-Al_2O_3 and to ITO. N_O for ITO is calculated to be 46.71 atoms of oxygen per nm^3, and for α-Al_2O_3 is calculated to be 70.64 atoms of oxygen per nm^3. L_{O1s} for ITO is estimated to be 1.53 nm as previously discussed, and 1.9 nm for α-Al_2O_3, also estimated using the NIST EAL Database.[1] The surface concentration of uncoordinated P-OH can then be approximated as a fraction of this signal.

$$\varphi_{P-OH} = f * N_O * L_{O1s} * \cos(\theta)$$

Equation S4

where f is the fraction of the overall O1s signal from uncoordinated P-OH. Compared to the preceding XPS model used to calculate X-2PACz surface coverage, this model omits the term for the instrument specific sensitivity factor and the attenuation through the X-2PACz film, since these are identical for both oxygens within uncoordinated P-OH groups and the substrate.

***Table S2**: O1s Uncoordinated P-OH Peak Percentages and Monte Carlo Standard Deviations - ITO, with quantifiable levels of uncoordinated P-OH in green and unquantifiable levels in orange*

Sample	Angle	% of Overall O1s Peak	Std. Dev.	Quantifiable?	Weighted Avg. P-OH Areal Density (molecules per nm^2)	Weighted Avg. Std. Dev. (molecules per nm^2)
2PACz	0°	1.15	1.01	Y	0.91	0.90
	40°	2.94	2.53	Y		
	55°	3.79	3.24	Y		
	70°	3.03	5.55	N		
F-2PACz	0°	0.02	0.08	N	0.66	0.75
	40°	1.23	0.96	Y		
	55°	1.84	1.21	Y		
	70°	2.14	3.32	N		
Cl-2PACz	0°	0.05	0.05	N	0.78	0.89
	40°	1.67	1.09	Y		
	55°	1.66	1.53	Y		
	70°	3.96	4.23	N		
Br-2PACz	0°	0.26	0.15	Y	0.18	0.15
	40°	1.65	1.45	Y		
	55°	2.56	2.79	N		
	70°	4.14	2.51	Y		
I-2PACz	0°	1.19	0.57	Y	0.79	0.57
	40°	1.05	1.24	N		
	55°	1.12	2.1	N		
	70°	3.48	4.03	N		
^{t}Bu-2PACz	0°	1.31	1.08	Y	0.98	0.86
	40°	2.92	2.67	Y		
	55°	3.05	2.12	Y		
	70°	3.59	2.73	Y		

***Table S3**: O1s Uncoordinated P-OH Peak Percentages and Monte Carlo Standard Deviations - α-Al_2O_3*

Sample	Angle	% of Overall O1s Peak	Std. Dev.	Quantifiable?	Weighted Avg. P-OH Areal Density (molecules per nm^2)	Weighted Avg. Std. Dev. (molecules per nm^2)
2PACz	0°	1.08	0.22	Y	1.35	0.15
	40°	1.27	0.27	Y		
	55°	1.58	0.39	Y		
	70°	2.51	0.65	Y		
F-2PACz	0°	1.46	0.28	Y	1.46	0.17
	40°	1.19	0.28	Y		
	55°	1.55	0.45	Y		
	70°	1.57	0.62	Y		
Cl-2PACz	0°	0.38	0.36	Y	0.81	0.29
	40°	0.95	1.17	Y		
	55°	1.76	0.48	Y		
	70°	2.34	2.45	N		
Br-2PACz	0°	0.44	0.54	N	0.78	0.26
	40°	0.7	0.68	Y		
	55°	1.1	0.31	Y		
	70°	1.06	0.69	Y		
I-2PACz	0°	0.42	0.57	N	1.02	0.28
	40°	0.45	0.38	Y		
	55°	2.07	0.47	Y		
	70°	4.54	0.93	Y		
^{t}Bu-2PACz	0°	0.83	0.47	Y	1.08	0.34
	40°	1.43	1.23	Y		
	55°	1.22	0.58	Y		
	70°	2.27	1.61	Y		

The comparative increase in the uncertainty in the ITO P-OH areal densities is primarily attributable to the greater degree of peak overlap present in the ITO O1s spectra compared to that of α-Al_2O_3.

S2. X-Ray Reflectivity

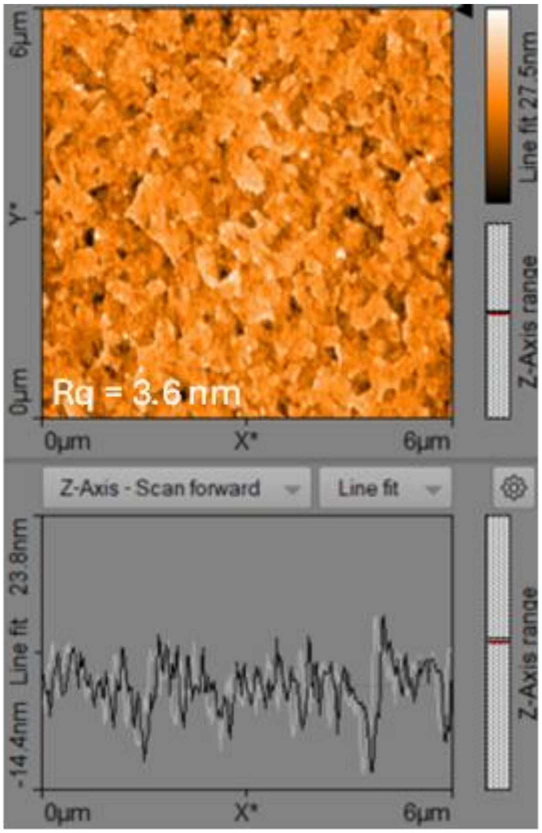

Figure S8: *AMF and RMS Roughness of Pristine ITO*

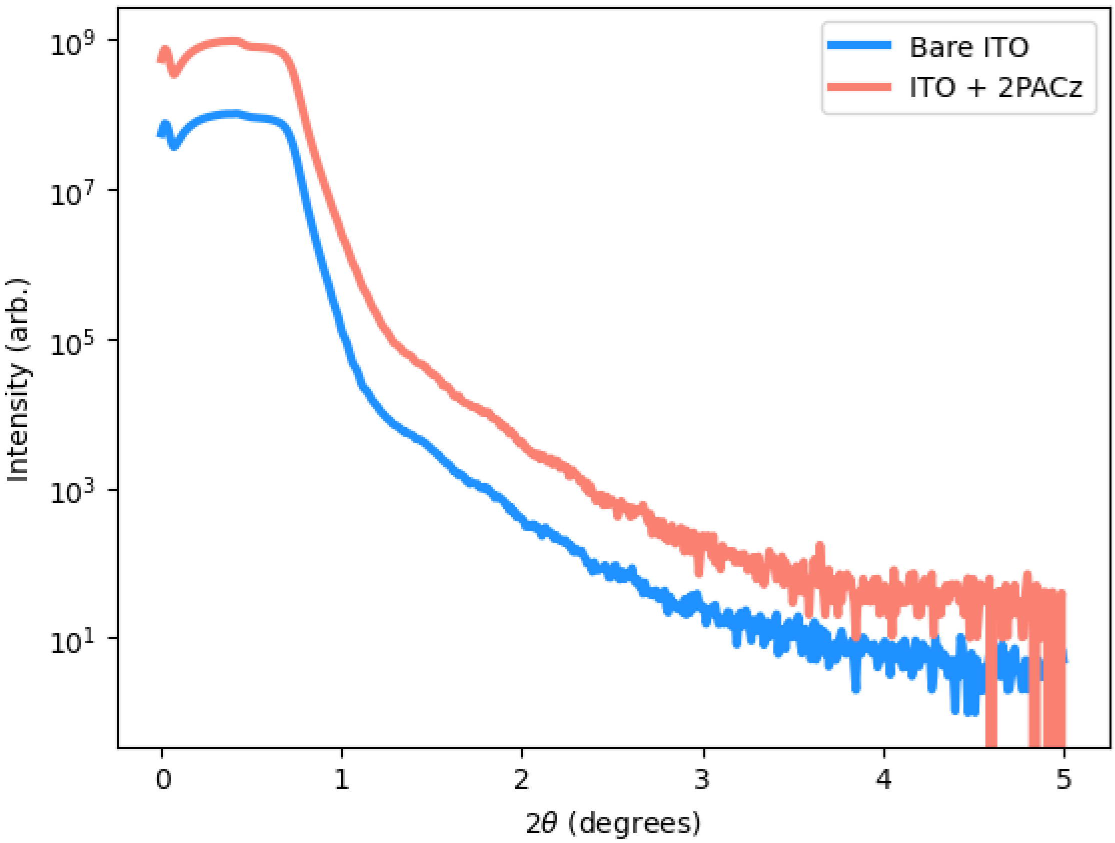

***Figure S9**: XRR plot of pristine ITO vs 2PACz modified ITO.*

XRR Fitting Technique

Several methods for fitting XRR data for self-assembled monolayers have been reported in the literature, ranging from simple 1-layer fits[2] to multilayer models which attempt to capture the variation of electron density between different moieties in a SAM.[3] In our study, we explored several methods for fitting the reflectivity data, ranging from simple one-layer fits to the granular atom-by-atom model reported in the main text.

The one-layer fits performed well for modeling the reflectivity profiles of each SAM studied, see Figure S10 and S11 below, with the reduced reflectivity data and fits shown in Figure S12 and the numerical results displayed in Table S4.

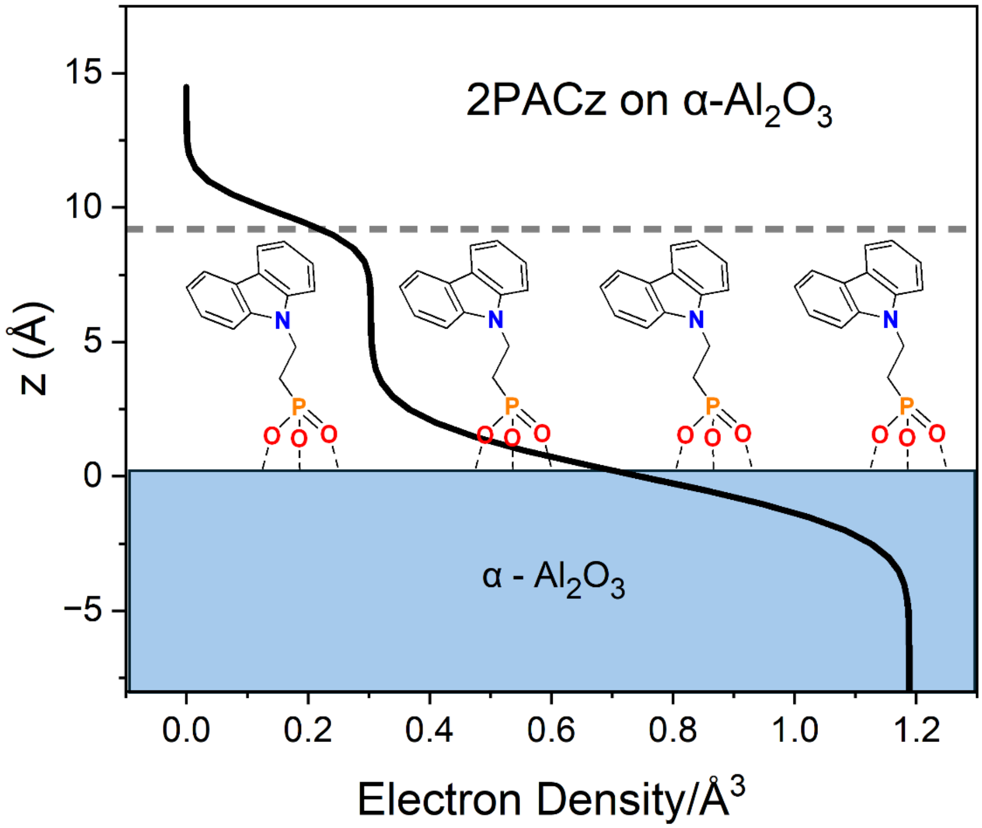

Figure S10:*1-layer electron Density Profile of 2PACz on α-Al2O3.*

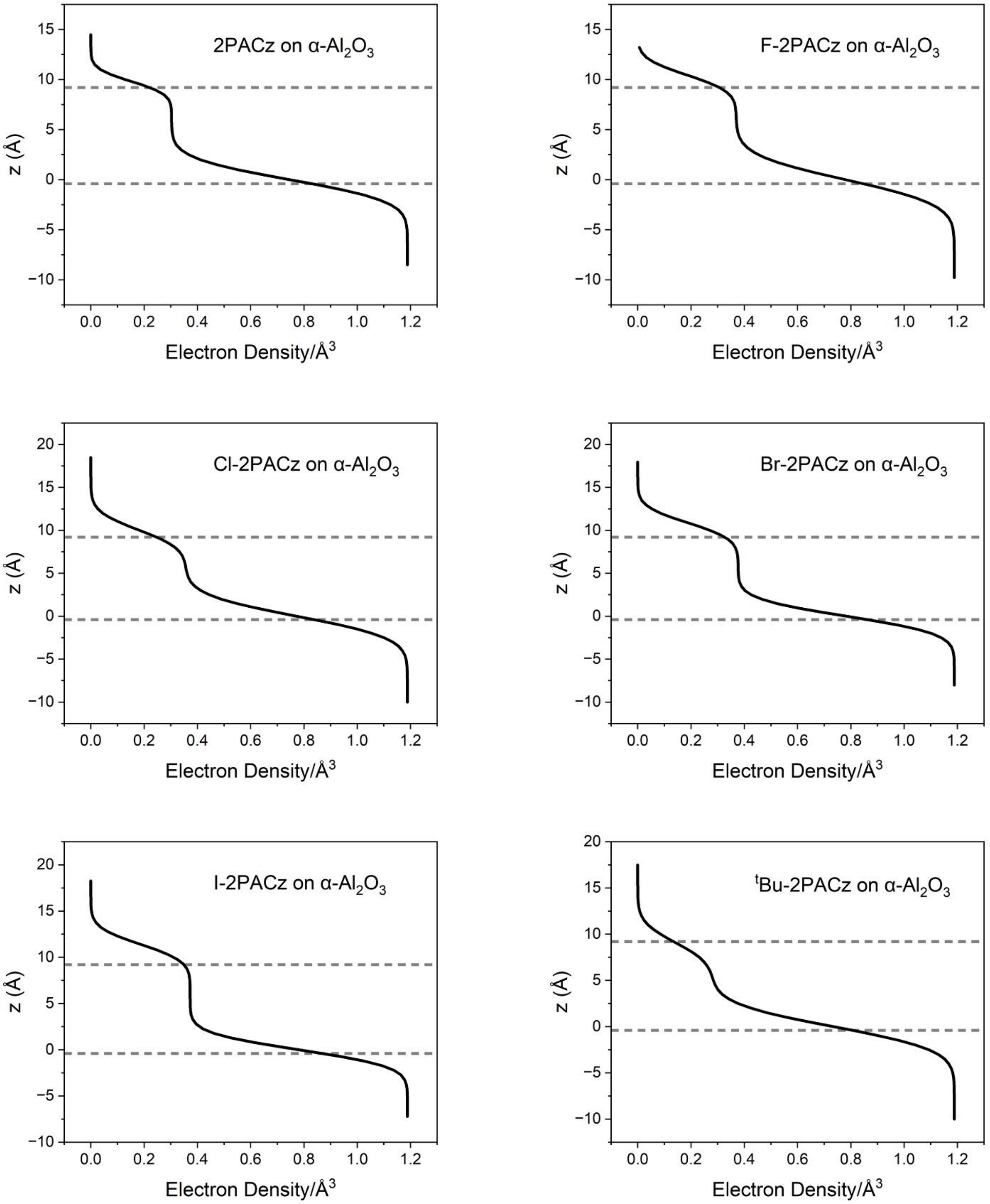

***Figure S11**: Single Layer XRR Electron Density Profiles of each X-2PACz.*

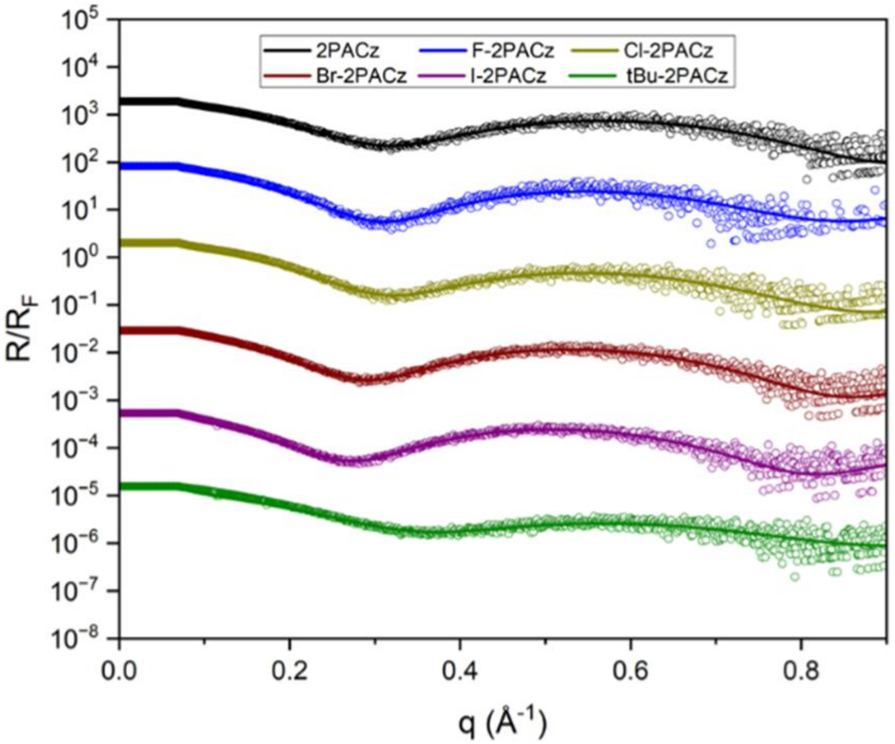

Figure S12:*Single-Layer Fit Reduced Reflectivity Data and Fits for X-2PACz on α-Al_2O_3*

Table S4: *Single-Layer Fit Results for XRR of X-2PACz on α-Al2O3*

Molecule	2PACz layer Thickness (Å)	Surface Coverage (Molecules/nm^2)	Surface Roughness (Å)
2PACz	10.1	2.4 ± 0.4	1.5
F 2PACz	10.4	2.4 ± 0.3	2.1
Cl 2PACz	10.1	2.0 ± 0.3	1.8
Br 2PACz	10.9	1.9 ± 0.2	1.5
I 2PACz	11.4	1.7 ± 0.1	1.5
tBu 2PACz	9.1	1.2 ± 0.4	2.0

While useful in establishing a baseline reflectivity profile for each X-2PACz layer, a one-layer model may obfuscate important details of monolayer structure. To capture the electron density profile of the SAM layers in as much detail as possible, an alternative method for modeling the SAM on a nearly atom-by-atom basis was developed. First, X-2PACz molecules were modeled in IQMol with an z-axis defined as aligned along the molecule's P-N axis. This allows for each X-2PACz molecule to be divided into multiple 1-Å-thick sub-layers along the z-axis based on their atomic coordinates. The number of electrons in each sub-layer is then determined by localizing electron density at the atomic coordinates and summing the electron density contributions of each atom. Electron density ratios are then determined between each sub-layer. It is worth noting that an artefact of this treatment is that a sub-layer of zero electron density can emerge in sub-layers where no atomic centers are located, although with an interfacial width between sub-layers, the sub-layer electron density becomes non-zero.

This model is used as a basis in GenX-3 to simulate reflectivity profiles. The roughness of each layer in this fit is set equal to the substrate roughness (around 2 Å). To simulate a molecular tilt of the X-2PACz, the sub-layer thicknesses are decreased uniformly. A representation of this treatment and visualization of these sub-layers is depicted in Figure S13. The initial electron density of the whole film, informed by the single layer electron density layer fit, is adjusted and iterated until a good fit is attained, being careful to maintain the previously established electron density ratios between the different sub-layers. The goodness of fit is judged by the minimization of the logarithmic figure of merit discussed in the main text. Results of the figure of merit for each molecule and the results of the fitted granular XRR models are shown in the following Table S5, with the electron density profile of each granular fit shown in Figure S14.

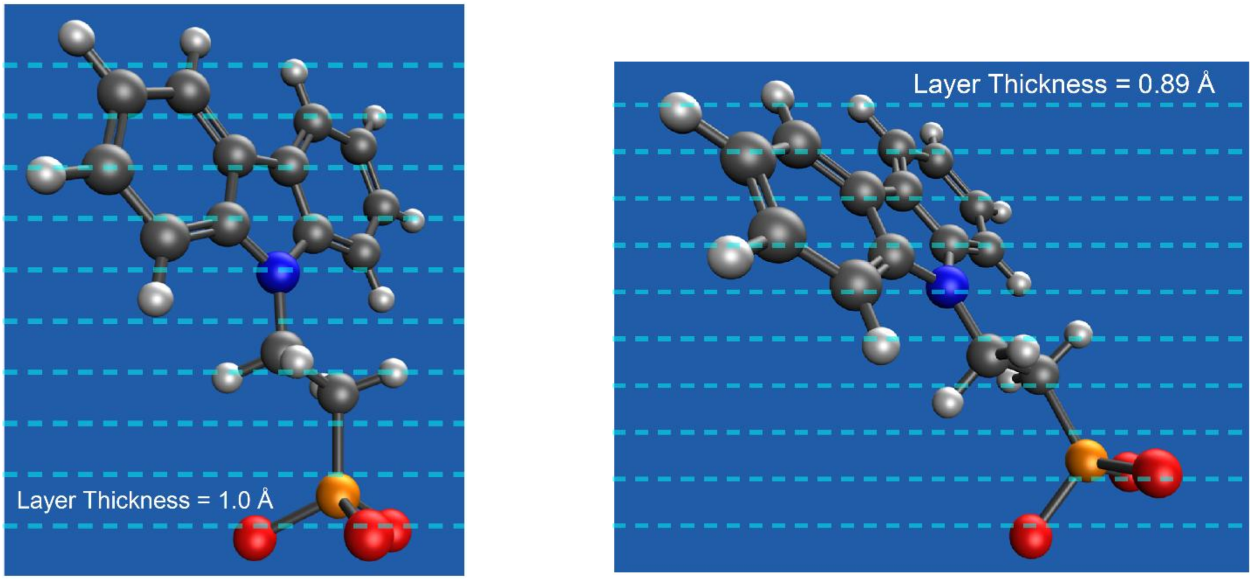

***Figure S13**: Granular 2PACz model, showing how the tilt of the molecule manifests as smaller individual sub-layers.*

Table S5: *Granular X-2PACz XRR Fits*

2PACz Fit Parameters (FOM = 0.105)

Layer (Stoichiometry)	Thickness (Å)	Electron density (e^-/A^{-3})	Roughness (Å)
11 (H_2)	0.89	0.051	2.0
10 (C_3H_1)	0.89	0.49	2.0
9 (C_4H_1)	0.89	0.64	2.0
8 (C_2)	0.89	0.31	2.0
7 (C_3N_1)	0.89	0.64	2.0
6 (H_2)	0.89	0.051	2.0
5 (C_1H_1)	0.89	0.18	2.0
4 (C_1H2)	0.89	0.20	2.0
3 (N/A)	0.89	0	2.0
2 (P_1)	0.89	0.39	2.0
1 (O_3)	0.89	0.66	2.0
Substrate (α-Al_2O_3)	Infinite	1.18	2.0

F-2PACz (FOM = 0.109)

Layer (stoichiometry)	Thickness (Å)	Electron density (e^-/A^{-3})	Roughness (Å)
11 (F_1H_2)	0.87	0.27	2.1
10 (F_1C_2)	0.87	0.51	2.1
9 (C_5)	0.87	0.73	2.1
8 (C_2H_1)	0.87	0.32	2.1
7 ($C_3N_1H_1$)	0.87	0.63	2.1
6 (H_2)	0.87	0.048	2.1
5 (C_1H_2)	0.87	0.20	2.1
4 (C_1H_2)	0.87	0.20	2.1
3 (N/A)	0.87	0	2.1
2 (P_1)	0.87	0.37	2.1
1 (O_3)	0.87	0.63	2.1
Substrate (α-Al_2O_3)	Infinite	1.18	2.1

Cl-2PACz (FOM = 0.090)

Layer (stoichiometry)	Thickness (Å)	Density (e^-/A^{-3})	Roughness (Å)
11 (Cl_1)	0.87	0.40	2.4
10 (Cl_1H_2)	0.87	0.45	2.4
9 (C_3)	0.87	0.43	2.4
8 (C_4H_1)	0.87	0.59	2.4
7 (C_4H_1)	0.87	0.59	2.4
6 ($C_1N_1H_1$)	0.87	0.33	2.4
5 (C_1H_1)	0.87	0.17	2.4
4 (C_1H_4)	0.87	0.24	2.4
3 (N/A)	0.87	0	2.4
2 (P_1)	0.87	0.36	2.4
1 (O_3)	0.87	0.62	2.4
Substrate (α-Al_2O_3)	Infinite	1.18	2.4

Br-2PACz (FOM = 0.0934)

Layer (stoichiometry)	Thickness (Å)	Density (e^-/A^{-3})	Roughness (Å)
11 (Br_1)	0.83	0.69	2.3
10 (Br_1H_2)	0.83	0.73	2.3
9 (C_3)	0.83	0.35	2.0
8 (C_4H_1)	0.83	0.49	2.0
7 (C_4H_1)	0.83	0.49	2.0
6 ($C_1N_1H_1$)	0.83	0.27	2.0
5 (C_1H_1)	0.83	0.14	2.0
4 (C_1H_4)	0.83	0.20	2.0
3 (N/A)	0.83	0	2.0
2 (P_1)	0.83	0.29	2.0
1 (O_3)	0.83	0.51	2.0
Substrate(α-Al_2O_3)	Infinite	1.18	2.0

I-2PACz (FOM = 0.0953)

Layer (stoichiometry)	Thickness (Å)	Electron density (e^-/A^{-3})	Roughness (Å)
11 (I_1)	0.85	0.93	2.3
10 (I_1H_2)	0.85	0.96	2.3
9 (C_3)	0.85	0.32	2.3
8 (C_4H_1)	0.85	0.44	2.3
7 (C_4H_1)	0.85	0.44	2.3
6 ($C_1N_1H_1$)	0.85	0.25	2.3
5 (C_1H_1)	0.85	0.12	2.3
4 (C_1H_4)	0.85	0.18	2.3
3 (N/A)	0.85	0	2.3
2 (P_1)	0.85	0.26	2.3
1 (O_3)	0.85	0.46	2.3
Substrate (α-Al_2O_3)	Infinite	1.18	2.3

tBu-2PACz (FOM = 0.112)

Layer (stoichiometry)	Thickness (Å)	Electron density (e^-/A^{-3})	Roughness (Å)
13 (H_1)	0.70	0.015	2.0
12 (C_1H_3)	0.70	0.13	2.0
11 (C_3H_7)	0.70	0.37	2.0
10 (C_3H_8)	0.70	0.38	2.0
9 (C_4)	0.70	0.35	2.0
8 (C_3H_3)	0.70	0.31	2.0
7 (C_4H_1)	0.70	0.37	2.0
6 ($C_1N_1H_1$)	0.70	0.21	2.0
5 (C_1H_1)	0.70	0.10	2.0
4 (C_1H_4)	0.70	0.15	2.0
3 (N/A)	0.70	0	2.0
2 (P_1)	0.70	0.24	2.0
1 (O_3)	0.70	0.37	2.0
Substrate (α-Al_2O_3)	Infinite	1.18	2.0

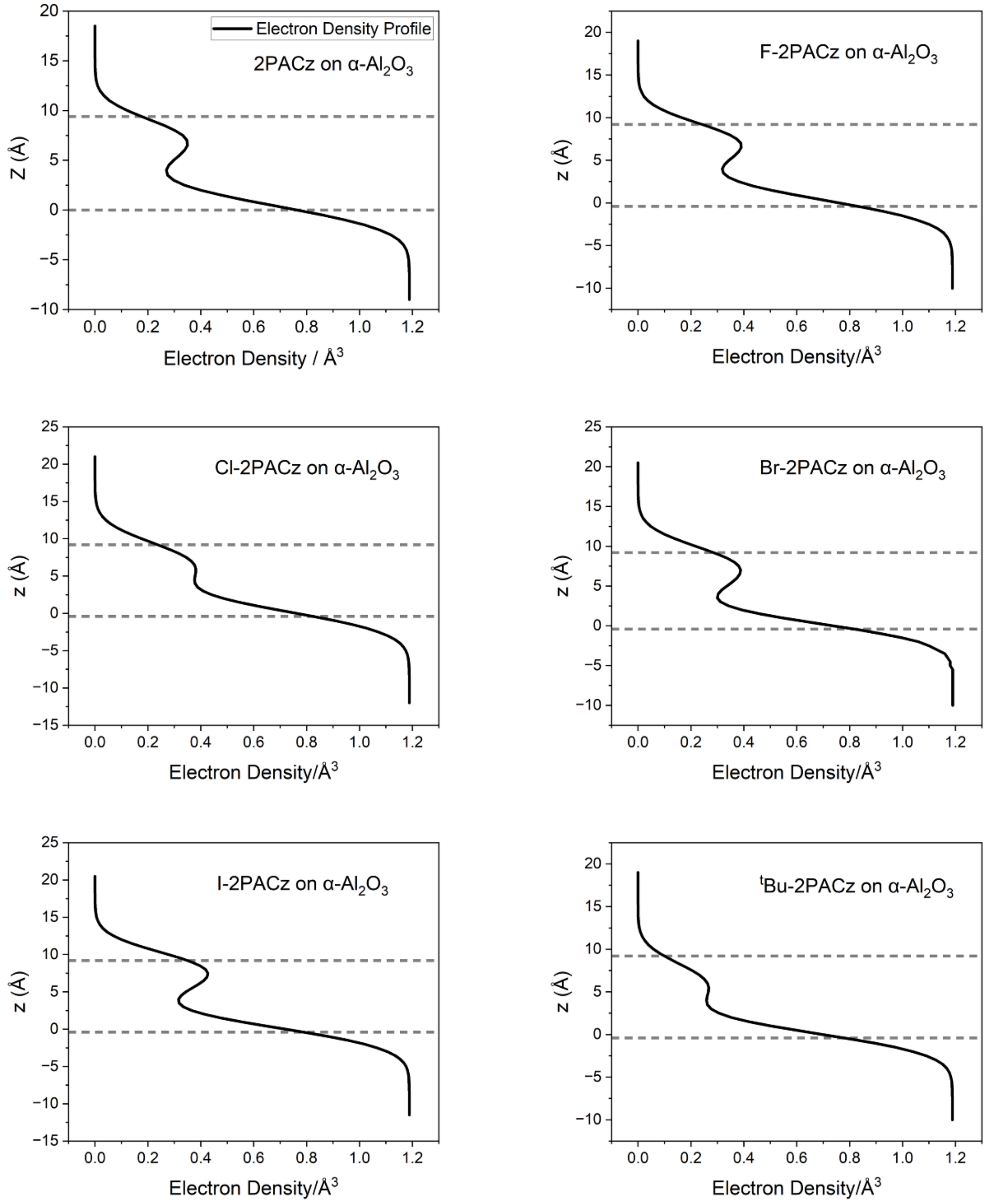

Figure S14*: Multi-layer, granular electron density profiles of a) 2PACz, b) F-2PACz, c) Cl-2PACz, d) Br-2PACz,*

Surface Coverage Determination from X-ray Reflectivity

Surface coverage, or areal density, as expressed in molecules per nm^2, is derived from the electron density and thickness of each layer according to Eq. S5.

$$Areal\ Density = \rho_{e^-}\, h/A(SAM)$$

Equation S5

where ρ_{e^-} is the average electron density per cubic angstrom over the X-2PACz layer, acquired by taking the sum of the electron density in each sub-layer and dividing by the number of layers in the fit, *A(SAM)* is the number of electrons per molecule, and *h* is the layer thickness.

X-ray Reflectivity Error Bar Determination

Error bars on surface coverage derived from XRR, noted in Tables 2 and S4, and Figure 6, were determined by taking the thickness of the monolayer, and perturbing it both higher and lower in thickness until a 5% increase in the FOM was achieved. This variation in the thickness was then applied to the calculation for surface coverage, with the subsequent range in coverages calculated used to denote the error bars in the XRR fit for surface coverage.

Estimation of Sterically Limited Coverage

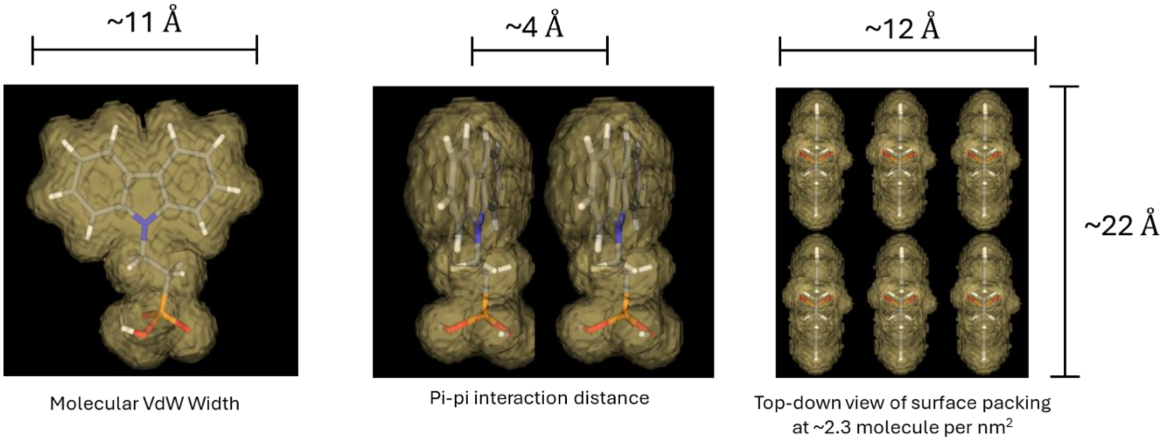

Figure S15*: Depiction of 2PACz VdW volume, and estimation of sterically limited packing density, assuming rectangular molecular packing.*

Figure S15 depicts the estimation for the sterically limited packing density of 2PACz on a planar substrate, assuming the molecules pack into a rectangular lattice. Taking the VdW width of the molecule (11Å), and the documented pi-pi interaction distance (4Å),[4] to arrive at a per molecule footprint of 0.44 nm, or a sterically limited surface coverage of 2.27 molecules per nm.[2] The molecular model used for this determination is documented in section S5.

S3. NEXAFS Spectroscopy Analysis

Both total and partial electron yield (TEY and PEY, respectively) collection modes were used for this experiment. TEY is a NEXAFS collection mode amenable to conductive samples and measures the drain current from a sample which replaces the emitted photoelectrons and secondary electrons under x-ray illumination.[5] This collection mode is inherently sensitive to the top few nanometers of a sample, as the information depth corresponds to the escape depth of photoelectrons. PEY mode, by comparison, uses a detector to directly measure emitted photoelectrons. In PEY mode, a bias is applied to a retarding grid on the analyzer, rejecting electrons below a certain kinetic energy threshold. This allows for greater surface sensitivity, rejecting secondary electrons which originate deeper a sample and have their energy attenuated in their path of escape. This retarding grid also allows for the measurement of non-conductive samples, as it screens electrons from an external flood gun that provides charge compensation. TEY collection mode was used for samples on ITO as this is conductive, and PEY mode for α-Al_2O_3. Although the information depths of these two collection modes are slightly different, (PEY is slightly more surface sensitive than TEY), both are governed by the same photoemission processes. By selectively investigating the C1s to C=C π* signal, which is only present in the top 1 nm of the sample in the SAM and not in the substrate, the fitted tilt from either collection mode is insensitive to this difference in sampling depth.

Carbon K-edge NEXAFS signal processing is done using a double normalization approach.[5–7] In addition to normalizing to the beam flux, sampled by a gold mesh upstream of the sample acquisition chamber, normalization must also be done to account for any carbon contamination on the gold mesh. TEY scans of a photodiode placed on the sample bar are taken every few hours, which produces a signal to normalize the gold mesh scan. This isolates the effect

of any carbon contamination which may accumulate on the gold mesh over the course of the experiment. Using this normalization, the NEAXFS is Equation S6[5].

$$NEXAFS = \frac{Sample\ Current}{[Au\ Mesh\ Current]_{sample}} * \frac{[Au\ Mesh\ Current]_{calibration}}{[Photodiode]_{calibration}}$$

Equation S6

After this double normalization, tilt angle analysis requires the isolation of variations in the spot size of the beam, which includes the NEXAFS endstation at NSLS II, beamline 7 – ID – 1, and the high-throughput NEXAFS endstation at the soft x-ray beamline at the Australian Synchrotron. To accomplish this, the pre and post edge regions of each scan are set to 0 and 1, respectively.[6] Below the carbon K-edge, between 270 eV and 280 eV, there is no absorption and this is set to zero as a baseline. Above about 320 eV, excitations do not correspond to intra-orbital excitations and, therefore, are not influenced by the x-ray polarization. Scaling this region to 1 therefore allows the signal in each sample to be normalized proportional to the amount of carbon in the beam spot. This eliminates the influence of the amount of material probed on variations for features like the C=C π* intensity, allowing for a reliable determination of molecular orientation.

***Table S6**: NEXAFS Tilts and 95% Confidence Intervalsof Tilt Angle Fit for X-2PACz on α-Al_2O_3*

Molecule	Tilt Angle	95% Confidence Interval
2PACz	60.39°	± 0.33°
F-2PACz	63.73°	± 0.42°
Cl-2PACz	64.60°	± 0.47°
Br-2PACz	62.70°	± 0.36°
I-2PACz	63.10°	± 0.28°
^{t}Bu-2PACz	62.73°	± 0.34°

***Table S7**: NEXAFS Tilts and Standard Deviations of Tilt Angle Fit for X-2PACz on ITO, Separated by Synchrotron Used*

Molecule	Tilt Angle (NSLS-II)	95% Confidence Interval	Tilt Angle (ANSTO)	95% Confidence Interval
2PACz	62.30°	± 0.22°	61.21°	± 0.24°
F-2PACz	62.35°	± 0.24°	62.29°	± 0.28°
Cl-2PACz	65.43°	± 0.22°	65.08°	± 0.24°
Br-2PACz	63.51°	± 0.28°	63.49°	± 0.32°
I-2PACz	63.00°	± 0.28°	64.01°	± 0.34°
^{t}Bu-2PACz	61.82°	± 0.24°	60.77°	± 0.54°

S4. XPS analysis of SAM Coverage

This study uses an overlayer attenuation model to determine areal densities of each X-2PACz molecule on ITO, comparing the intensities of the P(2p) peak from each X-2PACz molecule and the In($3d_{5/2}$) peak from the ITO substrate and modeling the attenuation paths. This model expands upon the similar study from Lin et al.,[8] refining assumptions based on additional characterization data produced in our study. The aforementioned model assumes partial monolayer surface coverage, wherein there are regions of the bare substrate. Based on the findings discussed herein regarding X-ray reflectivity and X-2PACz layer structure, each X-2PACz layer forms a continuous film, with molecules evenly distributed across the substrate. In addition, models proposed by Lin et al.[8] and Paniagua et al.[9] utilize an estimation of the information depth within the ITO substrate of three attenuation lengths. While a good approximation, we treat the ITO substrate as an infinite slab, more quantitatively determining the signal from the substrate.

Treating the ITO substrate as an infinite slab, the signal of the In 3d 5/2 within the ITO substrate is:

$$I \propto N_{In} e^{\left(\frac{-x}{L_{In,ITO} cos(\theta)}\right)}$$

Equation S7

where I is the measured XPS intensity, N_{In} is the concentration of indium in ITO, x is the depth within the sample from which the signal originates, $L_{In,\ ITO}$ is the effective attenuation length of indium photoelectrons through ITO, and θ is the photoelectron takeoff angle. ITO here was modeled as Sn-doped In_2O_3 bixbyite, which is well established given that Sn substitution preserves the bixbyite lattice and generates small changes in the lattice parameter.[10] Assuming a concentration of 12.5%Sn in the lattice, typical of commercial ITO,[11] the conventional bixbyite

structure with 80 atoms per unit cell produces ITO with a stoichiometry of $In_{28}Sn_4O_{48}$. Using the lattice parameter of In_2O_3, 10.12 Å, produces a concentration of indium, N_{In}, of 27.04 atoms per nm^3 in the ITO substrate. The integration of equation S7 provides an estimate of the indium signal from the substrate:

$$N_{In}\int_0^{\infty} \exp\left(\frac{-x}{L_{In,ITO} * \cos(\theta)}\right)dx \propto N_{In}L_{In,ITO} * \cos(\theta)$$

Equation S8

The indium signal is attenuated by the X-2PACz layer according to Equation S9:

$$e^{\left(\frac{-x_{In}}{L_{In,X-2PACz}\, cos(\theta)}\right)}$$

Equation S9

Here, x_{In} is the thickness of the X-2PACz layer and $L_{In,\ X\text{-}2PACz}$ is the effective attenuation length of indium 3d 5/2 photoelectrons through the X-2PACz layer, with all else the same as Equation S8. Combined with the instrument specific sensitivity factor for indium 3d 5/2 ($SF_{In,\ 3d\ 5/2}$), the indium signal is:

$$In_{3d\ 5/2} \propto N_{In} * \cos(\theta) * L_{In,ITO} * SF_{In,3d\ 5/2} * \exp\left(\frac{-x_{In}}{L_{In,X-2PACz} * \cos(\theta)}\right)$$

Equation S10

where $In_{3d\ 5/2}$ is the measured signal intensity, N_{In} is the previously mentioned stoichiometric concentration of indium in the ITO substrate (27.04 nm^{-3}), and the remaining variables are as described before. Using the same approach, a similar expression is constructed for the P 2p signal, without the factor from substrate:

$$P_{2p} \propto \varphi_{X-2PACz} * SF_{P,2p} * \exp\left(\frac{-x_P}{L_{P,X-2PACz} * \cos(\theta)}\right)$$

Equation S11

where P_{2p} is the measured signal intensity, $\varphi_{X-2PACz}$ is the number of X-2PACz molecules per nm^2, $SF_{P,2p}$ is the instrument specific sensitivity factor for the phosphorus 2p signal, x_p is the thickness within the X-2PACz tail seen by the phosphorus photoelectrons, and $L_{P,\,X\text{-}2PACz}$ is the effective attenuation length of phosphorus 2p photoelectrons through the X-2PACz tail. The ratio of these is used to construct an expression for X-2PACz coverage.

$$\frac{P_{2p}}{In_{3d\ 5/2}} = \frac{\varphi_{X-2PACz}}{N_{In} * L_{In,ITO} * \cos(\theta)} * \frac{SF_{P,2p}}{SF_{In,3d\frac{5}{2}}} * \frac{\exp\left(\frac{-x_P}{L_{P,X-2PACz} * \cos(\theta)}\right)}{\exp\left(\frac{-x_{In}}{L_{In,X-2PACz} * \cos(\theta)}\right)}$$

Equation S12

which can then be rearranged to solve directly for X-2PACz coverage:

$$\varphi_{X-2PACz} = \frac{P_{2p}}{In_{3d\ 5/2}} * \frac{SF_{In,3d\frac{5}{2}}}{SF_{P\ 2p}} * \frac{\exp\left(\frac{-x_{In}}{L_{In,X-2PACz} * \cos(\theta)}\right)}{\exp\left(\frac{-x_P}{L_{P,X-2PACz} * \cos(\theta)}\right)} * N_{In} * L_{In,ITO} * \cos(\theta)$$

Equation S13

For this study, as mirrored in Lin et al.,[8] x_P is assumed to be dominated by the 2PACz tail group. Tail-group dimensions for non-rotated (short) configurations are measured from the linkage carbon bound to N in 2PACz to the terminal hydrogens in the tail group. Rotated tail group thicknesses are measured as the largest dimension between two points across the width of the tail

group, simulating the largest length in a rotated conformation. x_{In} is the 2PACz layer thickness from XRR. Parameters for dimensions of 2PACz are documented in Table S8.

***Table S8**: X-2PACz Tail Group Dimensions*

Molecule	MW	XRR Layer Thickness (Å)	Tail Group Height (Å)	Tail Group Width (Å)	NEXAFS Tilt (ITO)	Tilted Tail group Thickness, 0° Rotation (Å)	Tilted Tail Group Thickness, 90° Rotation (Å)	Avg. Tail Group Thickness (Å)
2PACz	275.24	10.2	5.84	8.88	62°	5.16	7.84	6.46
F-2PACz	311.22	10.4	5.84	8.88	62°	5.16	7.84	6.46
Cl-2PACz	344.13	10.1	5.84	9.07	65°	5.29	8.22	6.76
Br-2PACz	433.04	10.9	5.84	9.21	63°	5.29	8.21	6.76
I-2PACz	527.04	11.4	5.84	9.39	64°	5.25	8.44	6.85
^{t}Bu-2PACz	387.46	9.1	7.22	10.49	61°	6.31	9.17	7.74

The EAL for In 3d photoelectrons through ITO was additionally estimated at 1.53 nm. In our estimation of attenuation characteristics of P and In photoelectrons, the effective attenuation length (EAL), *L,* for each was determined for X-2PACz layers using the thicknesses noted above, as EAL is a weak function of layer thickness.[1] It should be noted that the EAL is different from the electron inelastic mean free path (IMFP), λ.[12] IMFP is solely a function of electron energy and material identity, but does not encapsulate additional factors relevant for an overlayer thickness estimation.[12] EAL incorporates the elastic scattering of materials as well as experimental factors such as instrument configuration. For these reasons, EAL can differ from IMFP and consideration

should be given to which is more relevant for a given analysis. For overlayer estimation, EAL is more appropriate.[12]

EAL is dependent on IMFP, and there exist several estimation techniques for both. We utilize the NIST database for both IMFP and EAL for each molecule.[1,13] The inputs for this include known variables, such as molecular formula and emission peak asymmetry factor, and experimentally derived or estimated quantities via calculations, such as material density and band gap. Simpler methods of determining EALs exist for organic materials but would fail to capture the differences inherent to the X-2PACz variants. EALs for $In_{3d, 5/2}$ and P_{2p} photoelectrons through X-2PACz are shown below in Table S9. The EAL for $In_{3d, 5/2}$ photoelectrons through ITO was also estimated at 1.53 nm.

***Table S9**: EALs for In 3d and P 2p photoelectrons through X-2PACz*

Surface Modifier	EAL – In($3d_{5/2}$) (nm)	EAL – P(2p) (nm)
2PACz	2.83	3.55
F-2PACz	2.83	3.48
Cl-2PACz	2.73	3.34
Br-2PACz	2.68	3.34
I-2PACz	2.65	3.36
^{t}Bu-2PACz	2.94	3.67

Band gaps for the 2PACz variants, a component of EAL estimation, were derived from solution UV-Vis measurements using Tauc plots, modeling a procedure employed by Lin et al.[14] These band gaps are shown in Table S10. The optical band gap of Br-2PACz has previously been

documented, and the value experimentally determined in this study is in good agreement with this existing value.[14] It should be noted that there are several means of band gap determination, and solution effects in our determination often produce smaller band gaps than those calculated for isolated molecules or molecular solids.[15] However, the EAL is not sensitive to the band gap, and so while there is some uncertainty in band gap for each molecule, these values present a reasonable approximation of EALs.

Table S10: *X-2PACz Solution Derived Band Gaps*

Surface Modifier	Band Gap (eV)
2PACz	3.50
F-2PACz	3.31
Cl-2PACz	3.30
Br-2PACz	3.32
I-2PACz	3.27
t-Bu-2PACz	3.40

Coverages were determined for each of three acquisition takeoff angles: 0º, 40°, and 55°. The rationale for not incorporating 70° is elaborated below. Table S11 reports the inverse variance weighed average of the surface coverage from these three scans. Error bars on these XPS derived coverage figures represent the quadrature sum of errors from three different parameters within the coverage model. These being the XPS peak intensity, the path length for indium photoelectrons, and the path length for phosphorus photoelectrons, whose uncertainties are determined from Monte Carlo uncertainties in XPS peak areas, the uncertainty in film thickness of each X-2PACz layer from the XRR fit, and the ± 3° associated with systematic variance in the NEXAFS tilts

applied to the carbazole tail group, respectively. Table S11 documents the total reported coverages and total error bars, while Table S12 and S13 and the following discussion describe the error contribution of each value described here.

Table S11*: Weighted Average Coverages for X-2PACz on ITO*

Molecule	Coverage Range, XPS (molecules per nm^2)
2PACz	2.20 ± 0.24
F-2PACz	2.02 ± 0.23
Cl-2PACz	1.91 ± 0.20
Br-2PACz	1.92 ± 0.23
I-2PACz	1.81 ± 0.21
^{t}Bu-2PACz	1.75 ± 0.30

***Table S12**: Error Analysis Example for Uncertainties in XPS Coverage Analysis*

Source of Uncertainty	Nominal Parameter	Perturbation Applied	Resulting Change in Coverage	Notes
XPS Intensity	$I_{P, 2p}$	$\pm\ \delta_I$	$\pm\ \delta\Gamma_I$	Derived from statistical uncertainty of peak fit
In Photoelectron Path Length	x_{In}	$\pm\ \delta_{X,In}$	$\pm\ \delta\Gamma_{X,In}$	Derived from statistical uncertainty of film thickness from XRR
P Photoelectron Path Length	x_P	$\pm\ \delta_{X,In}$	$\pm\ \delta\Gamma_{X,In}$	Derived by applying ±3° NEXAFS Tilt Uncertainty to molecular dimensions
Net Uncertainty			$\pm\ \delta\Gamma_{Total}$	Quadrature Sum

***Table S13**: Uncertainties in Coverage Calculation from XPS for Each X-2PACz Sample*

	Uncertainty in Coverage			
Molecule	P 2p XPS Intensity (molecules per nm^2)	In Photoelectron Path Length (molecules per nm^2)	P Photoelectron Path Length (molecules per nm^2)	Quadrature Sum (molecules per nm^2)
2PACz	± 0.21	± 0.09	± 0.07	± 0.24
F-2PACz	± 0.22	± 0.06	± 0.06	± 0.23
Cl-2PACz	± 0.18	± 0.06	± 0.07	± 0.20
Br-2PACz	± 0.21	± 0.05	± 0.07	± 0.23
I-2PACz	± 0.19	± 0.02	± 0.07	± 0.21
^{t}Bu-2PACz	± 0.25	± 0.14	± 0.08	± 0.30

Since the XPS coverage model employed uses information from our XRR and NEXAFS characterization, the uncertainties in physical quantities derived from these techniques (total layer thickness and X-2PACz tail group thickness) and utilized in the XPS coverage analysis must be considered. Uncertainty in the phosphorus signal was estimated using the standard deviation reported by CasaXPS for the quantified P2p region, which reflects sensitivity of the fitted peak area to statistical noise and background. We note that the comparatively low intensity of the phosphorus signal results in a larger uncertainty in the fit signal. Uncertainty in the indium 3d signal is much smaller due to the higher intensity. Therefore, the uncertainty in the XPS coverage analysis is dominated by that of the fitted P 2p peak area.

Layer thicknesses used in the XPS coverage calculation are derived from both XRR and NEXAFS. We use the XRR layer thickness to determine the layer thickness seen by indium photoelectrons. The uncertainty in this thickness is estimated as the SAM thickness variation which

produces a 5% change in the figure of merit for the XRR fit. Further elaboration is in section S2. The X-2PACz tail thickness seen by the phosphorus photoelectrons is calculated by multiplying the carbazole tail thickness by the cosine of the NEXAFS tilt for each molecule. The tilt angle uncertainty is ±3° and encapsulates systematic experimental uncertainty, which is propagated through to the X-2PACz tail thickness. The resulting thickness range for both total layer thickness and X-2PACz tail group thickness are incorporated into the XPS coverage model to determine the corresponding uncertainty in surface coverage. This is reported in Table S13.

Film densities used for this coverage estimation are derived from XRR on α-Al_2O_3 but, we acknowledge these values may be slightly different from the overlayer on ITO. These density estimates are used solely in the determination of NIST-based EAL. The EAL is, however, insensitive to X-2PACz layer density to a degree which would exceed the error bars in coverage due to the XPS intensity uncertainty. When determining the EAL, a hypothetical doubling of the X-2PACz layer density manifests as a <5% change in the coverage determined by the model. This doubling is not realistic but illustrates the conclusion that XRR derived densities are viable estimates for the determination of EALs. This is further validated by the similarity of surface coverages between the two substrates.

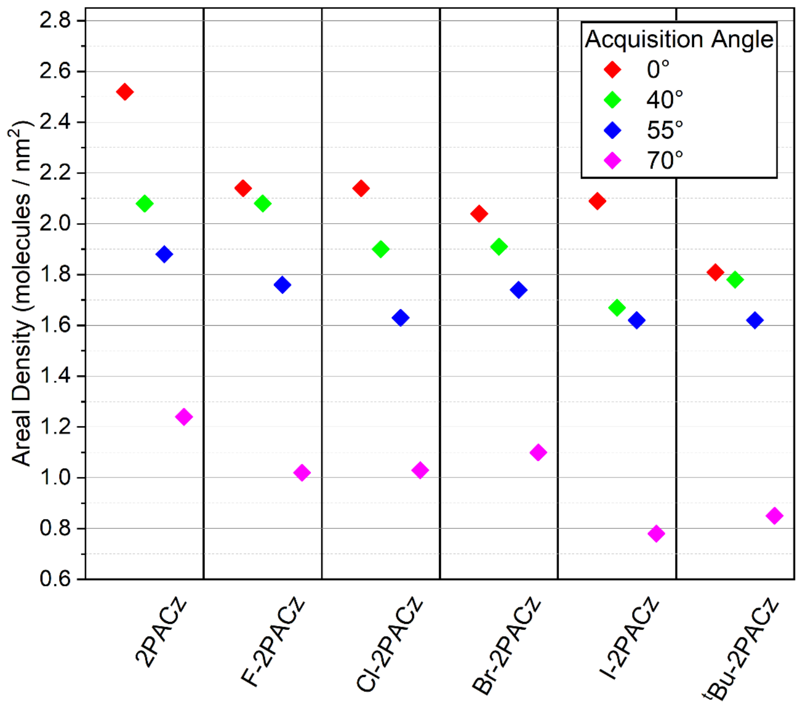

***Figure S16**: Angle resolved XPS coverage estimations*

With the XPS surface coverage model used here, differing angles should produce the same surface coverage. While this is true for the takeoff angles of 0°, 40°, and 55º, Figure S16 shows that the most extreme angle of 70° produces a lower calculated coverage, likely due to shadowing of the phosphorus signal. Shadowing on the nanoscale in angle resolved XPS can occur on rough surfaces, including substrates like ITO. As takeoff angles become larger, the topography of the surface can "shadow" phosphorus atoms under ITO features, additionally attenuating the emitted photoelectrons, which can cause significant variations in signal intensities in angle resolved XPS.[16,17] Because of this, scans at 70° have been omitted from the reported coverage values, which instead constitute the average of the three scans taken at 0°, 40°, and 55°. Previous reports have noted that at angles of 55°, roughness influences on measured XPS intensity are minimized, while shadowing dominates at sharper angles, further supporting this treatment of the coverage estimation.[18]

XPS Fitting Parameters

Fitting parameters for the O1s spectra on ITO are based on those published by Paramonov et al.[19], with peak widths allowed to vary slightly to better fit our data. FWHM values were varied while constraining peak position and shape according to literature values.[19–21] Table S14 shows fitting parameters for alumina O1s spectra. Descriptions of peak shapes can be found in the CasaXPS documentation. A(a,b,n)GL(p) is a method used to generate trailing tails on peaks to model photoelectron energy loss effects, and GL(p) represents a ratio of Gaussian and Lorentzian peak character, with GL(0) being pure Gaussian and GL(100) pure Lorentzian.

Table S14: *O1s XPS Fitting Parameters*

Species	Binding Energy	Peak Shape
Lattice ITO O	530.6	A(0.2, 0.65, 0) GL(55)
Surface ITO O	531.1	GL(0)
In-OH	532.3	GL(30)
In-O-P, P=O	532.1	GL(0)
P-OH	533.7	GL(30)
Lattice α-Al_2O_3	530.9	A(0.05, 0.3, 0) GL(5)
Al-OH	532.5	GL(30)
Al-O-P, P=O	532.4	GL(30)
P-OH	533.7	GL(30)

Indium peaks were modeled according to information in Peng et al.[22], including a 2 component fit of the In $3d_{5/2}$ peak with asymmetry to account for energy loss of the emitted photoelectrons.

***Table S15**: In and P XPS fitting parameters.*

Molecule	Binding Energy	Peak Allocation	Peak Shape
2PACz	444.4 eV	In 3d 5/2	GL(30)
	443.6 eV	In 3d 5/2	LA(2.1,3.05,0)
	133 eV	P 2p	GL(30)
F-2PACz	444.4 eV	In 3d 5/2	GL(30)
	443.6 eV	In 3d 5/2	LA(1.6,3.2,0)
	133 eV	P 2p	GL(30)
Cl-2PACz	444.4 eV	In 3d 5/2	GL(30)
	443.6 eV	In 3d 5/2	LA(1.4,3.0,0)
	133 eV	P 2p	GL(30)
Br-2PACz	444.4 eV	In 3d 5/2	GL(30)
	443.6 eV	In 3d 5/2	LA(1.4,3.0,0)
	133 eV	P 2p	GL(30)
I-2PACz	444.4 eV	In 3d 5/2	GL(30)
	443.6 eV	In 3d 5/2	LA(1.45,2.4,0)
	133 eV	P 2p	GL(30)
t-Bu-2PACz	444.4 eV	In 3d 5/2	GL(30)
	443.6 eV	In 3d 5/2	LA(1.6,3.2,0)
	133 eV	P 2p	GL(30)

S5. Molecular Models of X-2PACz Molecules

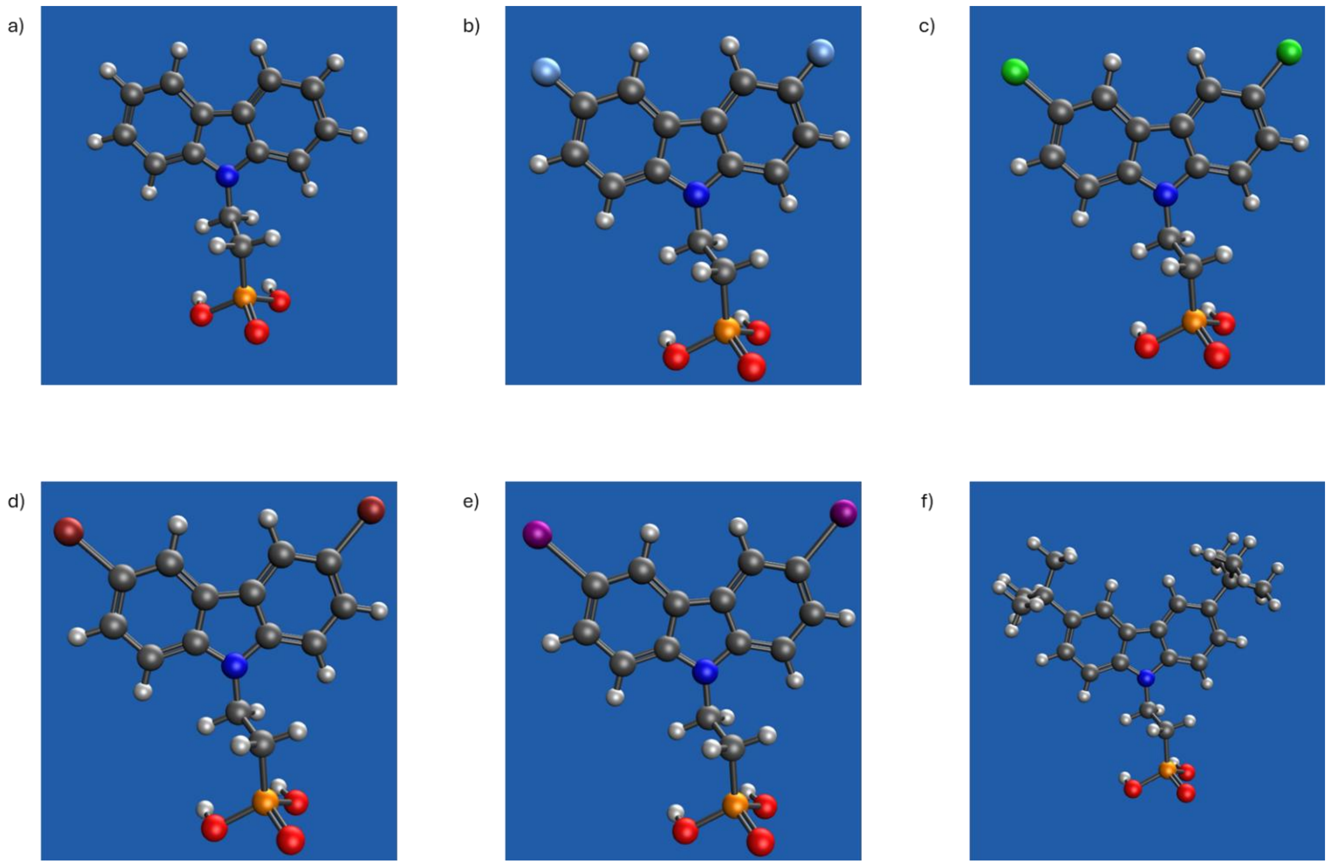

Figure S17: *IQmol Models of a) 2PACz, b) F-2PACz, c) Cl-2PACz, d) Br-2PACZ, e)I-2PACz, and f) t-Bu-2PACz.*

Table S16 lists the .xyz files of molecular models of each X-2PACz. The data represents atomic coordinates in units of angstroms.

Table S16: *XYZ Files of 2PACz Models*

2PACz

	X	Y	Z
C	-0.72606	-1.04164	-0.00069
C	-2.10008	-0.84136	-0.02014
C	-3.01756	-1.88980	-0.06089
C	-2.51834	-3.19816	-0.08279
C	-1.13064	-3.42908	-0.06391
C	-0.22129	-2.35666	-0.02282
C	-2.30798	0.51258	0.00794
C	-1.06264	1.09818	0.04377
C	-0.93047	2.49330	0.07870
C	-2.10040	3.27398	0.07606
C	-3.37062	2.66146	0.03919
C	-3.48448	1.26174	0.00455
N	-0.07112	0.16534	0.03940
C	1.35871	0.54670	0.07369
C	2.36539	-0.59789	0.06157
P	4.10162	0.07580	0.10698
O	5.14446	-1.07397	0.09531
O	4.52625	1.07090	-1.25261
O	4.48534	0.97989	1.53958
H	-3.20353	-4.03530	-0.11441
H	-4.26320	3.27344	0.03754
H	-4.08386	-1.70521	-0.07527
H	-0.75902	-4.44564	-0.08126
H	0.82102	-2.60352	-0.01045
H	0.03976	2.97091	0.10702
H	-2.02551	4.35330	0.10249
H	-4.45593	0.78570	-0.02388
H	1.53690	1.14569	0.99368
H	1.56779	1.19030	-0.80897
H	2.25965	-1.18501	-0.87420
H	2.23029	-1.23271	0.96164

F-2PACz

	X	Y	Z
C	-0.71099	-1.04532	0.00225
C	-2.08449	-0.84167	-0.01841
C	-3.00474	-1.88756	-0.06144
C	-2.50943	-3.19776	-0.08447
C	-1.12188	-3.43178	-0.06446
C	-0.20995	-2.36173	-0.02109
C	-2.28899	0.51271	0.01108
C	-1.04227	1.09525	0.04907
C	-0.90705	2.48982	0.08565
C	-2.07509	3.27337	0.08244
C	-3.34737	2.66431	0.04343
C	-3.46378	1.26452	0.00715
N	-0.05309	0.15988	0.04465
C	1.37816	0.53746	0.08084
C	2.38099	-0.61051	0.06595
P	4.12127	0.05349	0.11122
O	5.15753	-1.10192	0.08845
O	4.55155	1.04862	-1.24691
O	4.52186	0.94667	1.54647
F	-3.37040	-4.24414	-0.12625
F	-4.46368	3.43354	0.04079
H	-4.07038	-1.69874	-0.07667
H	-0.75134	-4.44880	-0.08267
H	0.83161	-2.61158	-0.00800
H	0.06430	2.96508	0.11557
H	-1.99615	4.35244	0.11008
H	-4.43574	0.78941	-0.02301
H	1.55780	1.13394	1.00212
H	1.58957	1.18223	-0.80037
H	2.27195	-1.19458	-0.87136
H	2.24388	-1.24715	0.96440

Cl-2PACz

	X	Y	Z
C	-0.67904	-1.04197	0.00574
C	-2.05167	-0.83300	-0.01438
C	-2.97632	-1.87472	-0.05925
C	-2.48671	-3.18771	-0.08463
C	-1.09924	-3.42684	-0.06459
C	-0.18352	-2.36028	-0.01933
C	-2.25084	0.52202	0.01728
C	-1.00195	1.09973	0.05570
C	-0.86157	2.49344	0.09422
C	-2.02626	3.28179	0.09253
C	-3.30179	2.67778	0.05283
C	-3.42290	1.27784	0.01457
N	-0.01652	0.16046	0.04907
C	1.41665	0.53255	0.08245
C	2.41469	-0.61950	0.06183
P	4.15890	0.03615	0.08809
O	5.18250	-1.13043	0.06223
O	4.60244	1.01387	-1.27775
O	4.59375	0.92988	1.51187
Cl	-3.60645	-4.53637	-0.14150
Cl	-4.74256	3.67771	0.05088
H	-4.04075	-1.67829	-0.07430
H	-0.72929	-4.44420	-0.08428
H	0.85706	-2.61421	-0.00604
H	0.11170	2.96481	0.12456
H	-1.93969	4.36036	0.12179
H	-4.39534	0.80340	-0.01628
H	1.60190	1.12717	1.00380
H	1.62761	1.17744	-0.79870
H	2.29607	-1.20434	-0.87375
H	2.28119	-1.25431	0.96220

Br-2PACz

	X	Y	Z
C	-0.66483	-1.04254	0.00207
C	-2.03695	-0.83111	-0.01062
C	-2.96330	-1.87157	-0.04852
C	-2.47558	-3.18516	-0.07456
C	-1.08851	-3.42644	-0.06206
C	-0.17085	-2.36117	-0.02370
C	-2.23370	0.52450	0.02026
C	-0.98379	1.09974	0.05065
C	-0.83996	2.49334	0.08683
C	-2.00293	3.28398	0.09118
C	-3.27967	2.68254	0.05968
C	-3.40409	1.28285	0.02362
N	-0.00002	0.15893	0.03992
C	1.43278	0.52904	0.06611
C	2.43101	-0.62276	0.04758
P	4.17182	0.04045	0.07432
O	5.19896	-1.12327	0.06232
O	4.62133	1.01545	-1.29118
O	4.59203	0.94055	1.49787
Br	-3.69730	-4.65173	-0.12714
Br	-4.84590	3.77460	0.06597
H	-4.02742	-1.67404	-0.05775
H	-0.72072	-4.44442	-0.08222
H	0.86964	-2.61592	-0.01596
H	0.13440	2.96272	0.11106
H	-1.91429	4.36230	0.11890
H	-4.37776	0.81097	-0.00078
H	1.62070	1.12627	0.98522
H	1.64135	1.17133	-0.81761
H	2.31381	-1.21100	-0.88606
H	2.29872	-1.25496	0.94999

I-2PACz

	X	Y	Z
C	-0.65604	-1.04329	0.00023
C	-2.02810	-0.83152	-0.01027
C	-2.95469	-1.87177	-0.04586
C	-2.46729	-3.18531	-0.07192
C	-1.08040	-3.42701	-0.06186
C	-0.16235	-2.36201	-0.02577
C	-2.22449	0.52412	0.02016
C	-0.97443	1.09908	0.04820
C	-0.83019	2.49269	0.08342
C	-1.99301	3.28350	0.08927
C	-3.26975	2.68227	0.06026
C	-3.39465	1.28273	0.02513
N	0.00911	0.15806	0.03660
C	1.44190	0.52786	0.06139
C	2.44004	-0.62403	0.04348
P	4.18068	0.03947	0.07330
O	5.20770	-1.12435	0.06166
O	4.63282	1.01700	-1.28953
O	4.59806	0.93677	1.49943
I	-3.81717	-4.80422	-0.12632
I	-4.99915	3.88826	0.06961
H	-4.01873	-1.67435	-0.05326
H	-0.71336	-4.44512	-0.08211
H	0.87810	-2.61696	-0.01991
H	0.14429	2.96190	0.10579
H	-1.90449	4.36174	0.11625
H	-4.36850	0.81142	0.00270
H	1.63014	1.12553	0.98013
H	1.65024	1.16968	-0.82274
H	2.32364	-1.21207	-0.89042
H	2.30699	-1.25646	0.94560

tBu-2PACz

	X	Y	Z
C	-0.84048	-1.06369	0.04719
C	-2.20716	-0.85168	-0.00283
C	-3.13636	-1.88878	-0.05384
C	-2.66989	-3.22792	-0.05418
C	-1.26498	-3.44392	-0.00163
C	-0.35067	-2.37853	0.04846
C	-2.40248	0.50647	0.00919
C	-1.15663	1.07787	0.06544
C	-1.01229	2.46645	0.09128
C	-2.16996	3.26072	0.05866
C	-3.47180	2.68198	-0.00003
C	-3.57275	1.26363	-0.02492
N	-0.17435	0.13634	0.08925
C	1.25804	0.50483	0.14761
C	2.25545	-0.64772	0.16462
P	3.99541	0.01523	0.22704
O	5.02178	-1.14898	0.25077
O	4.47850	0.97780	-1.13583
O	4.38112	0.92788	1.65232
H	-4.18582	-1.64026	-0.09145
H	-0.86389	-4.44822	0.00063
H	0.68915	-2.63309	0.08595
H	-0.03741	2.93347	0.13565
H	-2.04313	4.33424	0.07954
H	-4.52024	0.74903	-0.06891
H	1.42385	1.11088	1.06517
H	1.48929	1.13812	-0.73699
H	2.16050	-1.24507	-0.76581
H	2.10121	-1.27092	1.06979
C	-4.71045	3.60192	-0.03389
C	-4.64160	4.51193	-1.27932
H	-4.62489	3.89825	-2.20598
H	-5.52433	5.18628	-1.32279
H	-3.73494	5.15173	-1.27011
C	-4.73967	4.47205	1.24121
H	-5.62382	5.14594	1.23720
H	-4.79457	3.82932	2.14647
H	-3.83557	5.11035	1.32285
C	-6.04246	2.81626	-0.09822
H	-6.16169	2.16348	0.79351

H	-6.90893	3.51260	-0.12120
H	-6.09079	2.19183	-1.01655
C	-3.62433	-4.43979	-0.10877
C	-3.42419	-5.30917	1.15138
H	-3.64561	-4.71907	2.06700
H	-4.10079	-6.19105	1.13216
H	-2.38437	-5.68946	1.22636
C	-5.11584	-4.02989	-0.16574
H	-5.76937	-4.92854	-0.20450
H	-5.40215	-3.44700	0.73636
H	-5.32625	-3.42396	-1.07359
C	-3.31996	-5.27832	-1.36912
H	-3.99533	-6.15939	-1.42771
H	-3.46471	-4.66569	-2.28525
H	-2.27751	-5.65875	-1.36694

S6. UPS Work Function Measurements

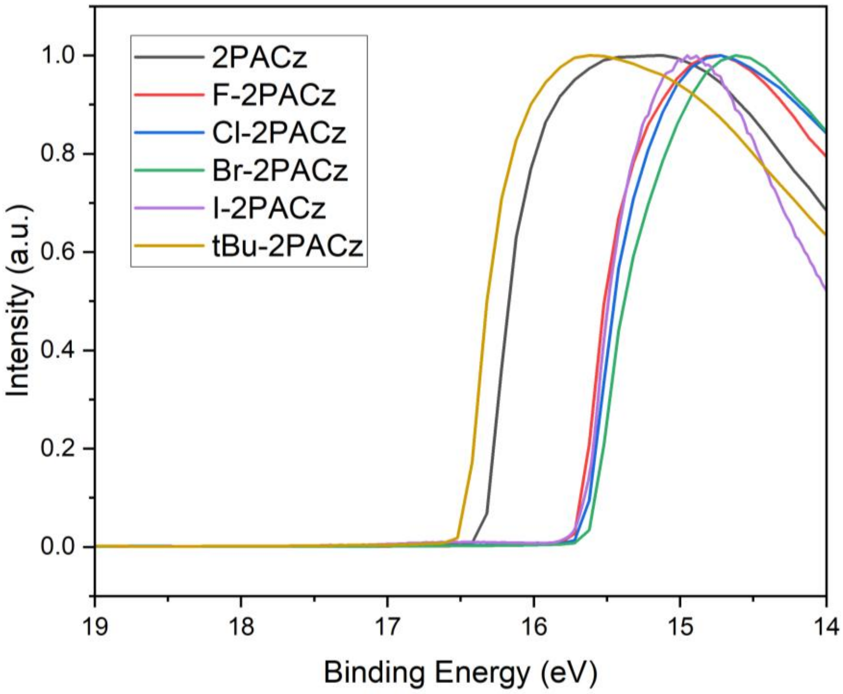

***Figure S18**: UPS Secondary Electron Cutoff Positions of 2PACz materials on ITO*

***Table S17**: Measured and reported work functions for 2PACz modified ITO*

Molecule	UPS Work Function of Modified ITO (eV)	
	this work	literature value
2PACz	5.22	5.17[23]
F-2PACz	5.71	5.70[24]
Cl-2PACz	5.85	5.78[24]
Br-2PACz	5.90	5.83[2], 5.81[14]
I-2PACz	5.65	5.74[24]
t-Bu-2PACz	4.92	5.06[23]

S7. NEXAFS Spectra and Angular Dependent Fits

Spectra and Tilt Angle Fits

All NEXAFS spectra x axes are in units of eV, with y axes intensities in arb. units. Angular dependent fits x axes are in units of rad^2, and y axes are in arb. units.

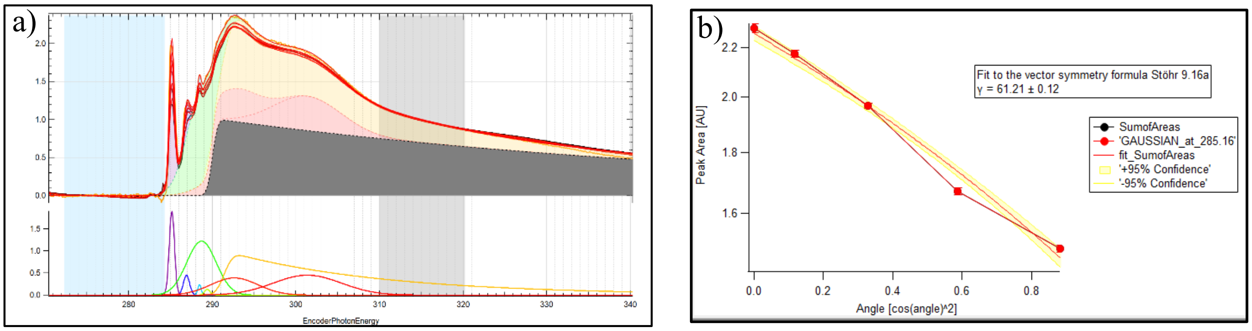

***Figure S19**: a) NEXAFS spectrum and fit and b) tilt angle fit for 2PACz on ITO, acquired at ANSTO-SXR*

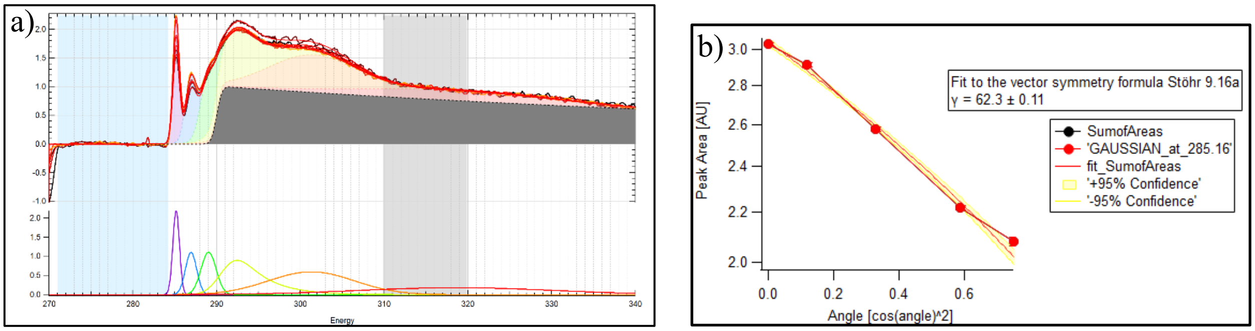

***Figure S20**: a) NEXAFS spectrum and fit and b) tilt angle fit for 2PACz on ITO, acquired at NSLS-II 7-ID-1*

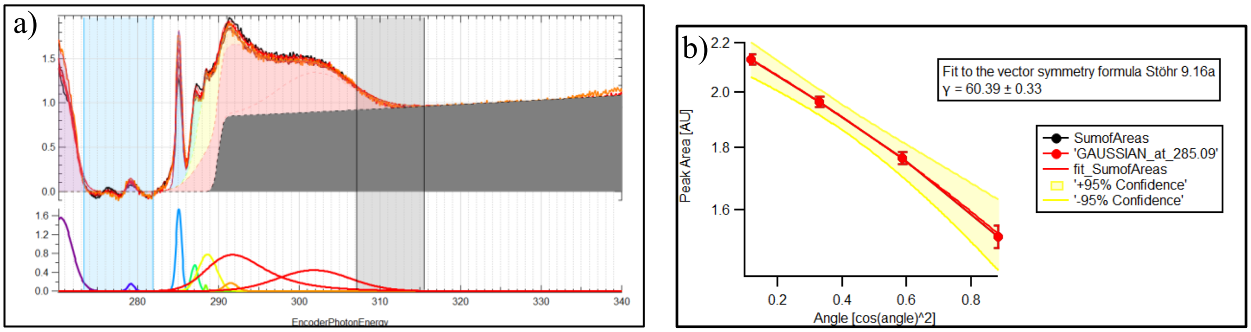

***Figure S21**: a) NEXAFS spectrum and fit and b) tilt angle fit for 2PACz on α-Al_2O_3, acquired at ANSTO-SXR*

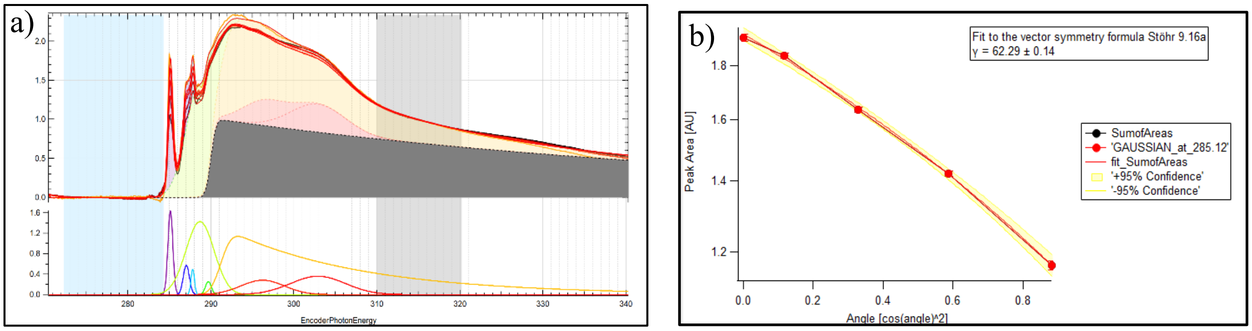

Figure S22: *a) NEXAFS spectrum and fit and b) tilt angle fit for F-2PACz on ITO, acquired at ANSTO-SXR*

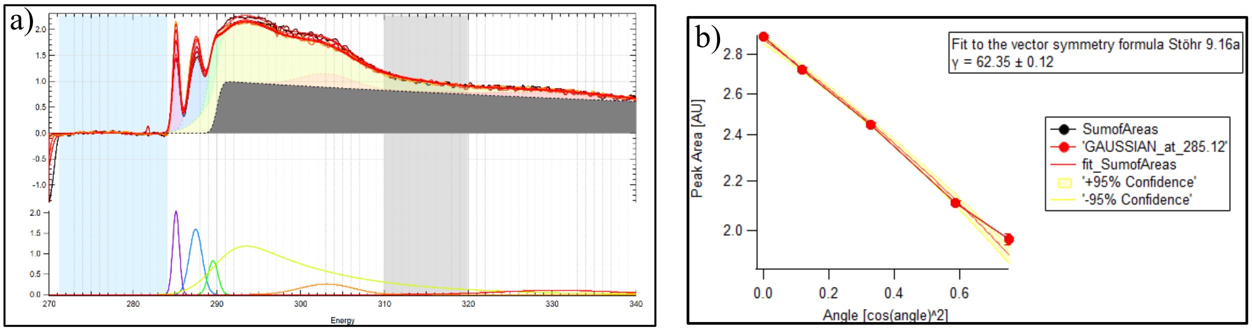

Figure S23: *a) NEXAFS spectrum and fit and b) tilt angle fit for F-2PACz on ITO, acquired at NSLS-II, 7-ID-1*

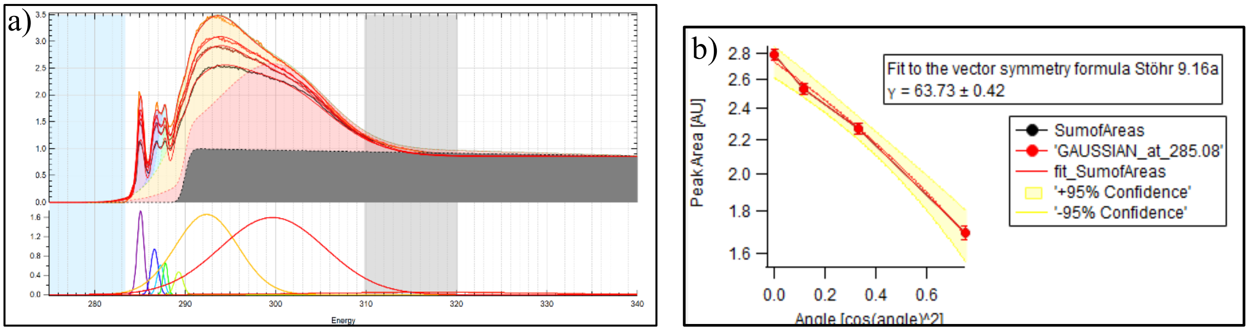

Figure S24: *a) NEXAFS spectrum and fit and b) tilt angle fit for F-2PACz on α-Al_2O_3, acquired at NSLS-II, 7-ID-1*

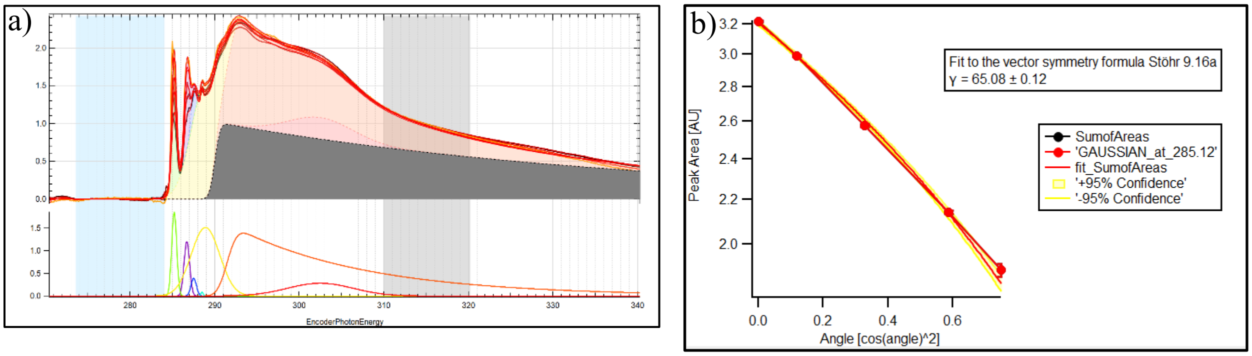

Figure S25: *a) NEXAFS spectrum and fit and b) tilt angle fit for Cl-2PACz on ITO, acquired at ANSTO-SXR*

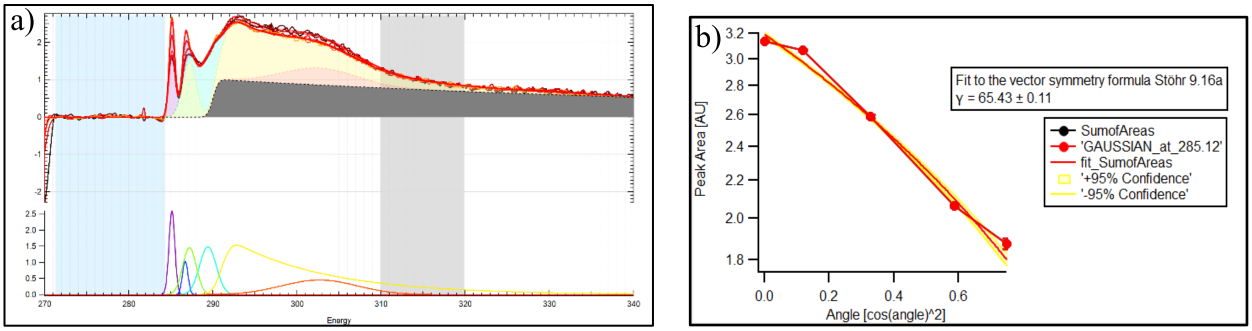

Figure S26: *a) NEXAFS spectrum and fit and b) tilt angle fit for Cl-2PCz on ITO, acquired at NSLS-II, 7-ID-1*

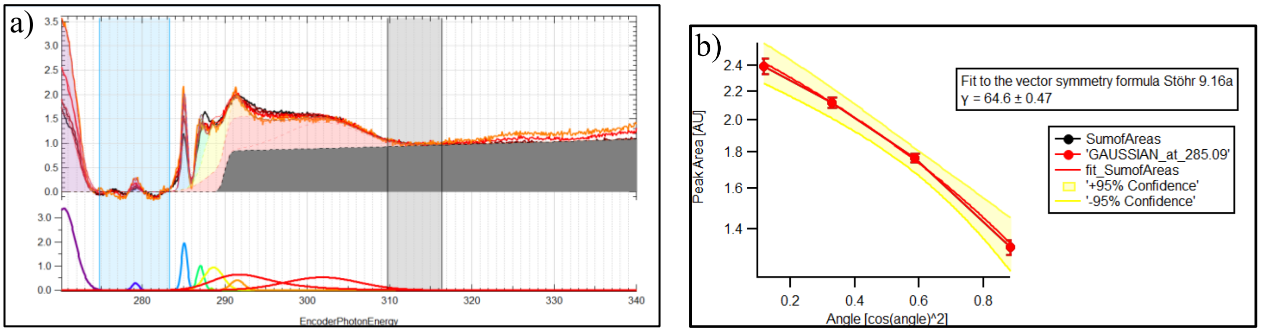

Figure S27: *a) NEXAFS spectrum and fit and b) tilt angle fit for Cl-2PACz on α-Al_2O_3, acquired at ANSTO-SXR*

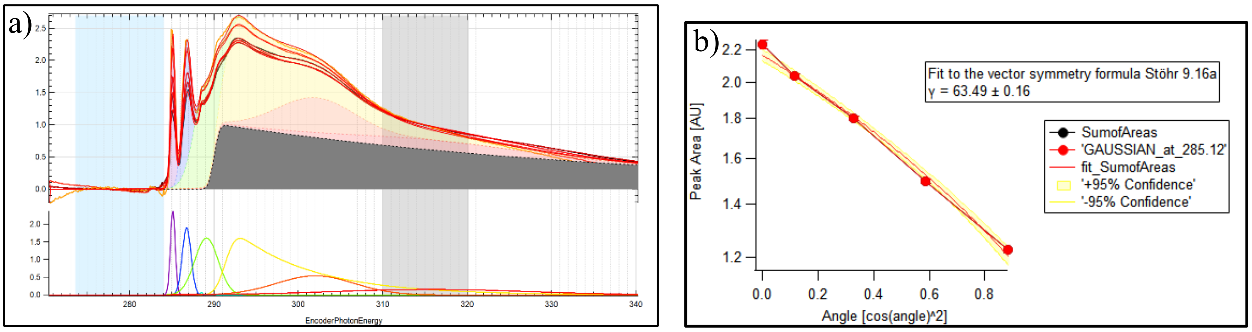

Figure S28: *a) NEXAFS spectrum and fit and b) tilt angle fit for Br-2PACz on ITO, acquired at ANSTO-SXR*

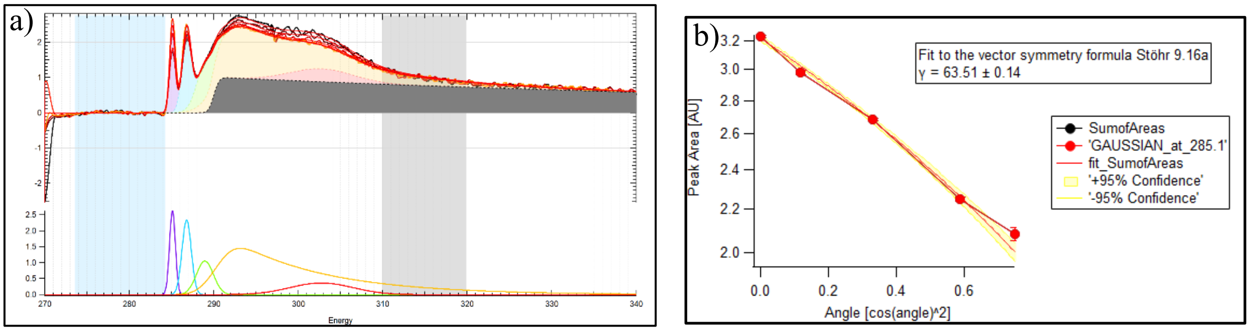

Figure S29: *a) NEXAFS spectrum and fit and b) tilt angle fit for Br-2PACz on ITO, acquired at NSLS-II, 7-ID-1*

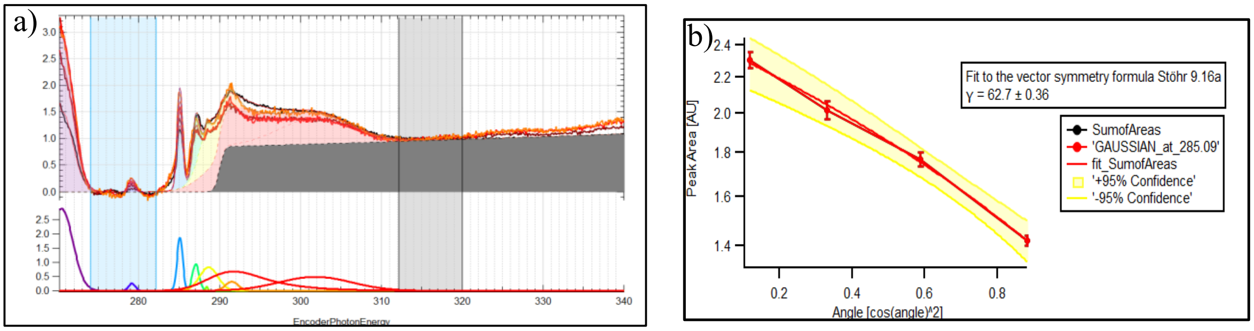

Figure S30: *a) NEXAFS spectrum and fit and b) tilt angle fit for Br-2PACz on α-Al_2O_3, acquired at ANSTO-SXR*

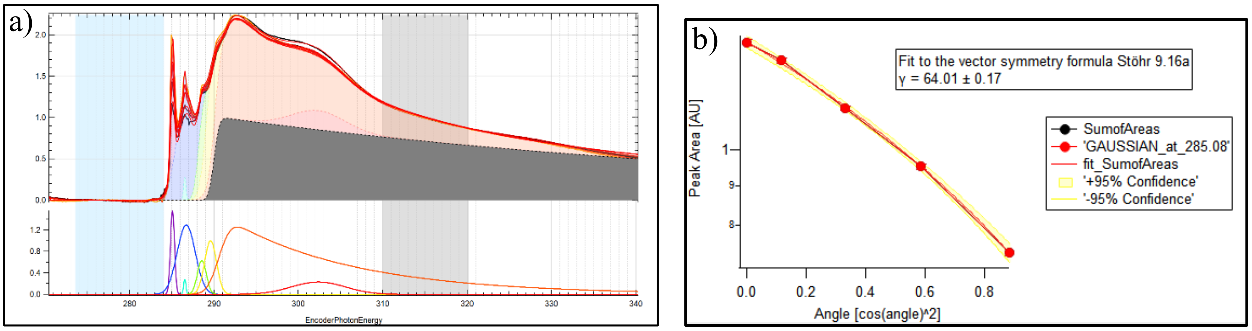

Figure S31: *a) NEXAFS spectrum and fit and b) tilt angle fit for I-2PACz on ITO, acquired at ANSTO-SXR*

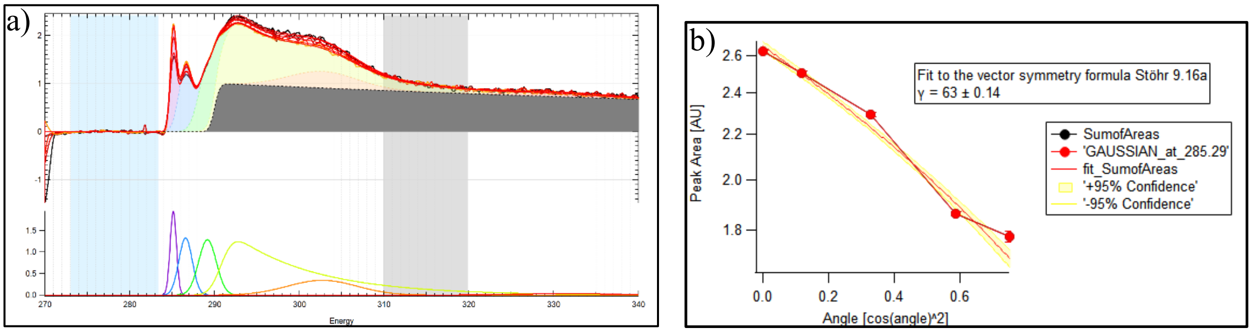

Figure S32: *a) NEXAFS spectrum and fit and b) tilt angle fit for I-2PACz on ITO, acquired at NSLS-II, 7-ID-1*

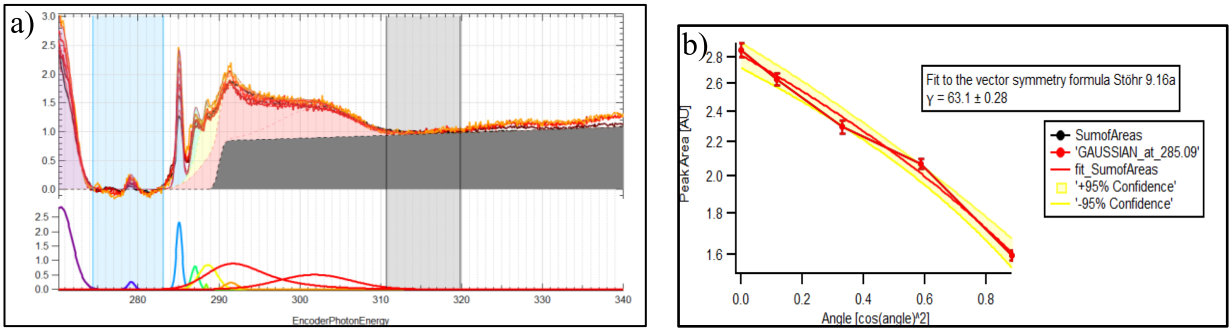

Figure S33: *a) NEXAFS spectrum and fit and b) tilt angle fit for I-2PACz on α-Al_2O_3, acquired at ANSTO-SXR*

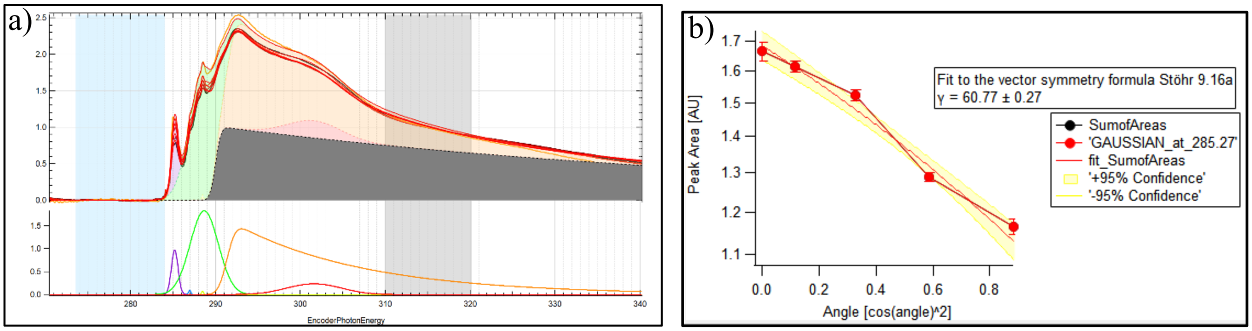

***Figure S34**: a) NEXAFS spectrum and fit and b) tilt angle fit for ᵗBu-2PACz on ITO, acquired at ANSTO-SXR*

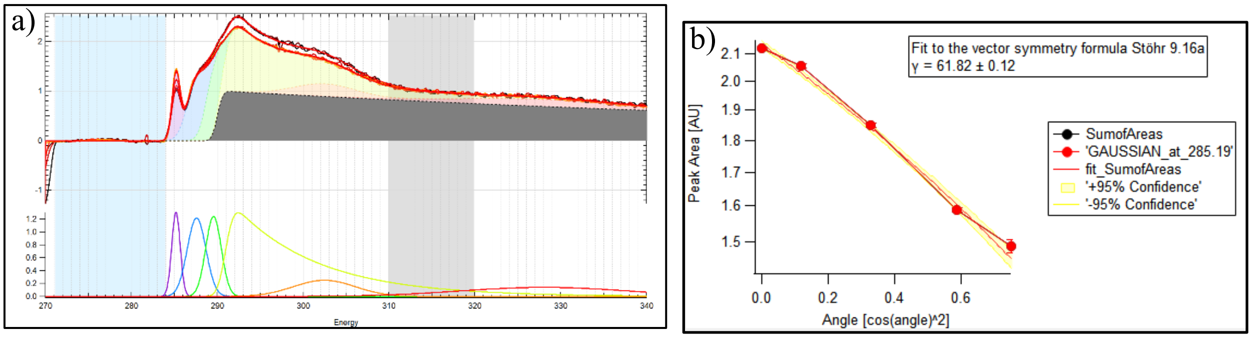

***Figure S35**: a) NEXAFS spectrum and fit and b) tilt angle fit for ᵗBu-2PACz on ITO, acquired at NSLS-II, 7-ID-1*

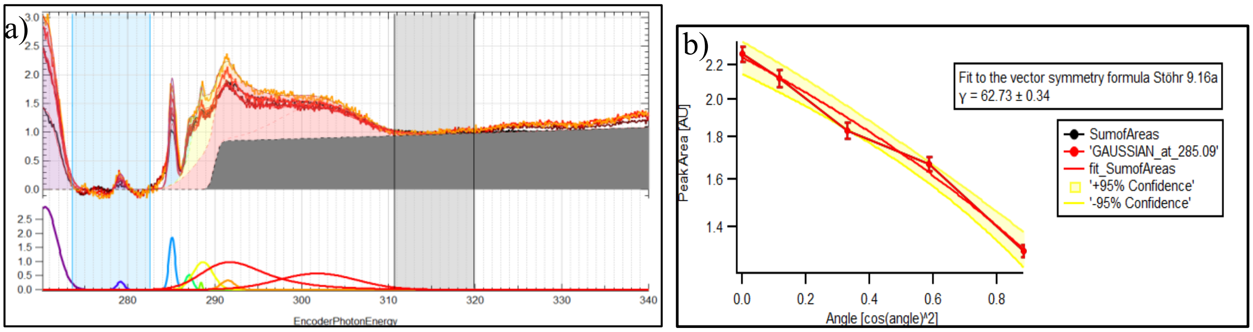

***Figure S36**: a) NEXAFS spectrum and fit and b) tilt angle fit for ᵗBu-2PACz on α-Al_2O_3, acquired at ANSTO-SXR*

Tilt Angle Fit Residuals

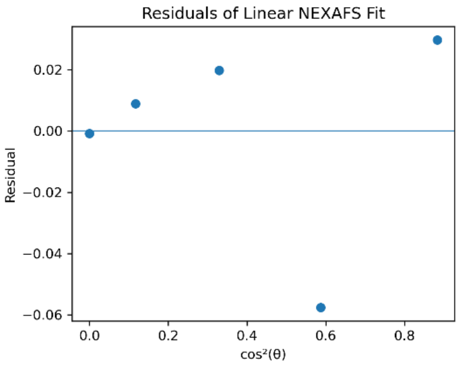

***Figure S37**: Tilt Angle Fit Residual of 2PACz on ITO Acquired at ANSTO-SXR*

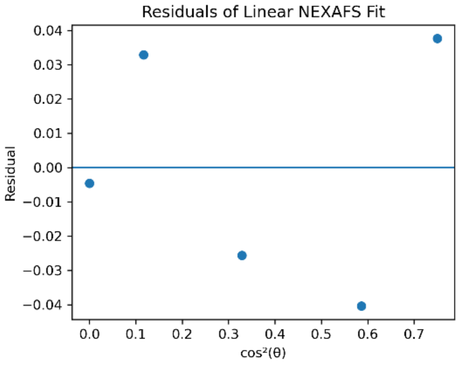

***Figure S38**: Tilt Angle Fit Residual of 2PACz on ITO Acquired at NSLS-II, 7-ID-1*

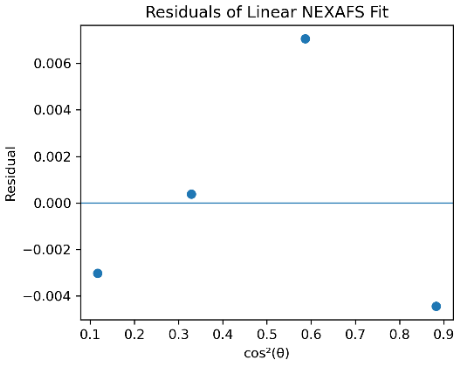

***Figure S39**: Tilt Angle Fit Residual of 2PACz on α-Al_2O_3 Acquired at ANSTO-SXR*

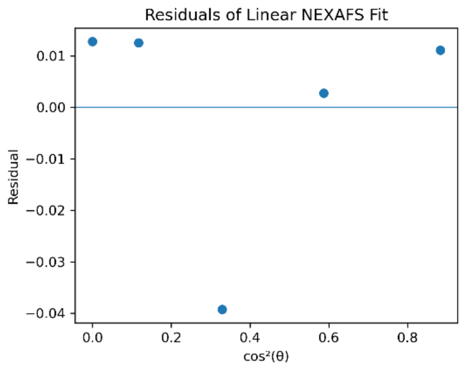

Figure S40: *Tilt Angle Fit Residual of F-2PACz on ITO, Acquired at ANSTO-SXR*

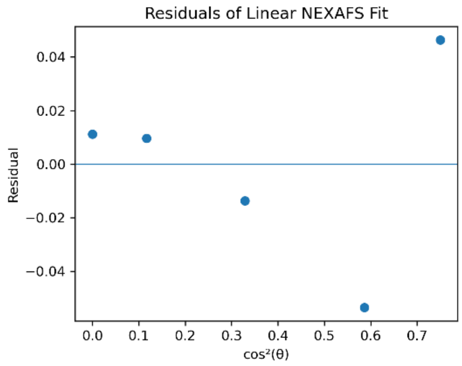

Figure S41: *Tilt Angle Fit Residual of F-2PACz on ITO, Acquired at NSLS-II, 7-ID-1*

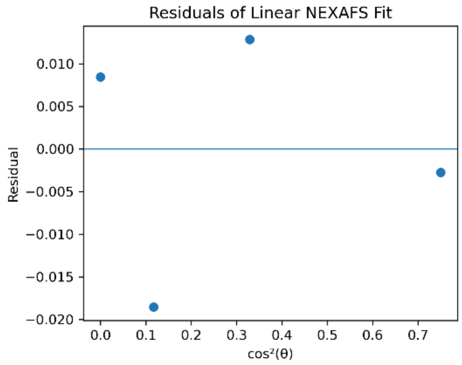

Figure S42: *Tilt Angle Fit Residual of F-2PACz on α-Al_2O_3, Acquired at NSLS-II, 7-ID-1*

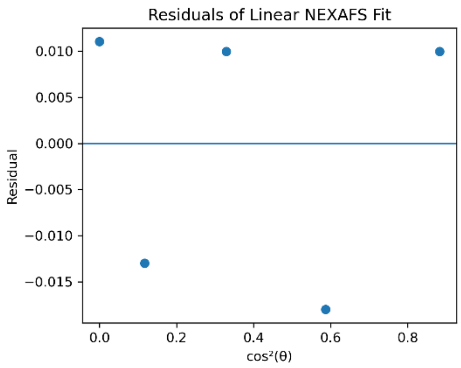

Figure S43*: Tilt Angle Fit Residual of Cl-2PACz on ITO, Acquired at ANSTO-SXR*

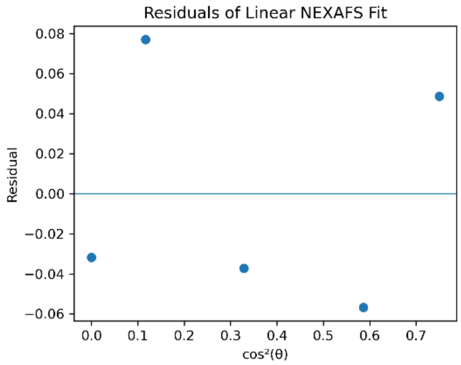

Figure S44*: Tilt Angle Fit Residual of Cl-2PACz on ITO, Acquired at NSLS-II, 7-ID-1*

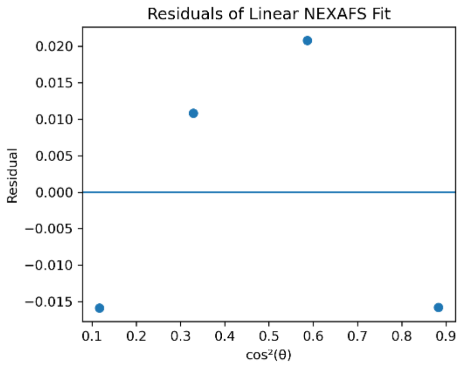

Figure S45*: Tilt Angle Fit Residual of Cl-2PACz on α-Al_2O_3, Acquired at ANSTO-SXR*

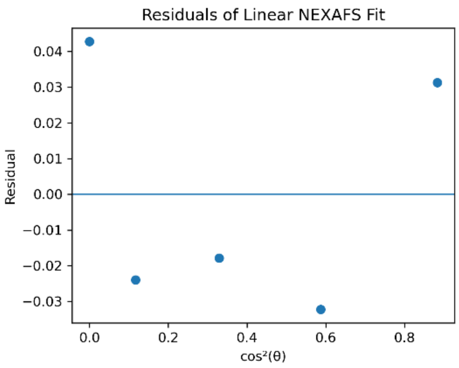

Figure S46: *Tilt Angle Fit Residual of Br-2PACz on ITO, Acquired at ANSTO-SXR*

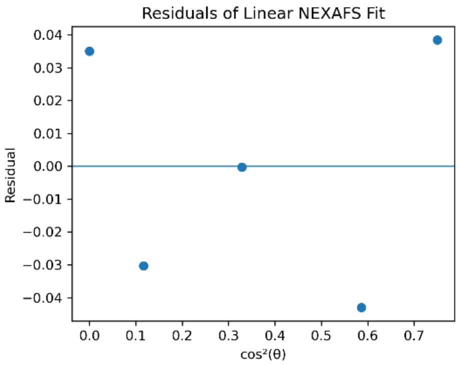

Figure S47: *Tilt Angle Fit Residual of Br-2PACz on ITO, Acquired at NSLS-II, 7-ID-1*

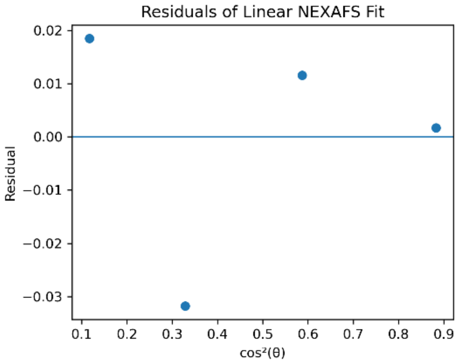

Figure S48: *Tilt Angle Fit Residual of Br-2PACz on α-Al_2O_3, Acquired at ANSTO-SXR*

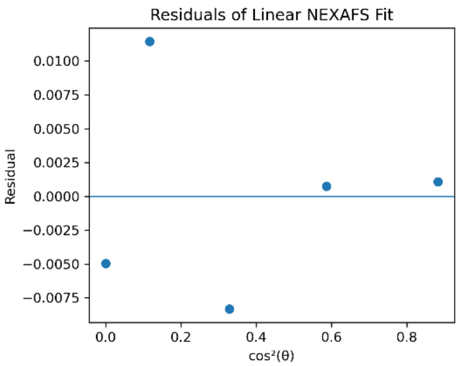

Figure S49*: Tilt Angle Fit Residual of I-2PACz on ITO, Acquired at ANSTO-SXR*

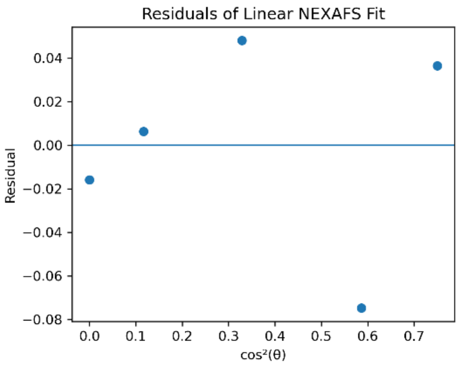

Figure S50*: Tilt Angle Fit Residual of I-2PACz on ITO, Acquired at NSLS-II, 7-ID-1*

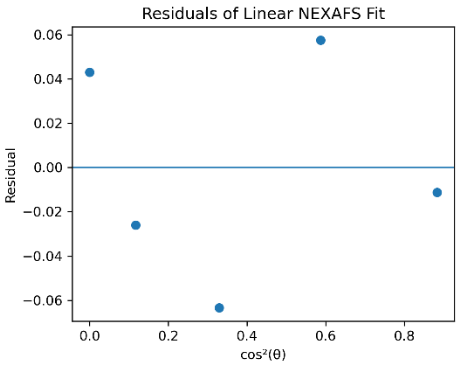

Figure S51*: Tilt Angle Fit Residual of I-2PACz on α-Al_2O_3, Acquired at ANSTO-SXR*

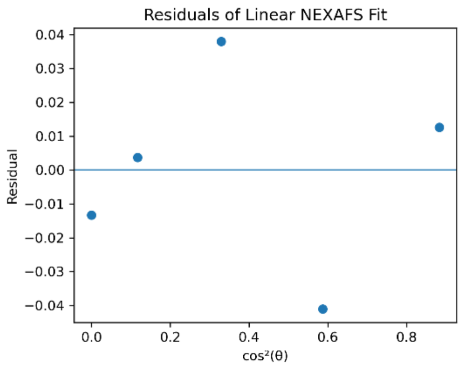

Figure S52: *Tilt Angle Fit Residual of ^{t}Bu-2PACz on ITO, Acquired at ANSTO-SXR*

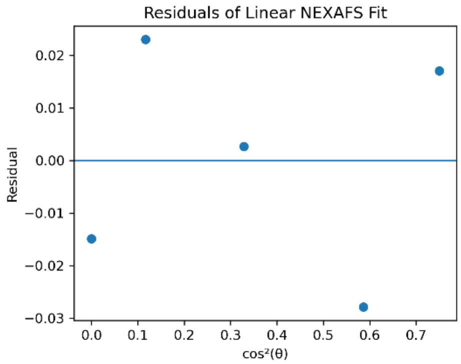

Figure S53: *Tilt Angle Fit Residual of ^{t}Bu-2PACz on ITO, Acquired at NSLS-II, 7-ID-1*

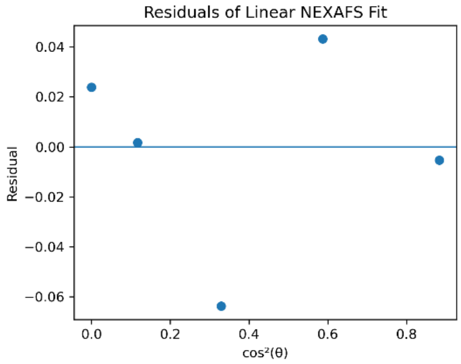

Figure S54: *Tilt Angle Fit Residual of ^{t}Bu-2PACz on α-Al_2O_3, Acquired at ANSTO-SXR*

S8. In 3d and P 2p XPS Spectra

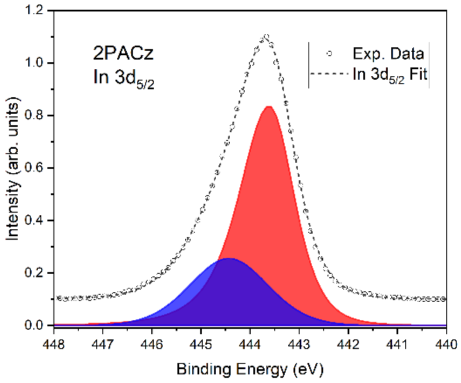

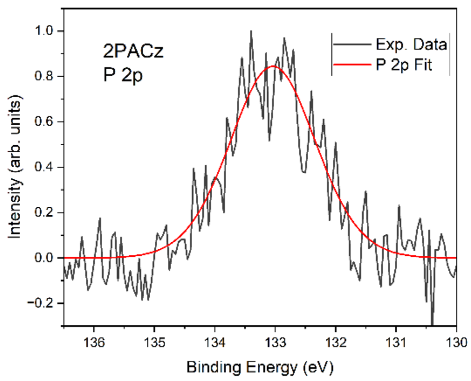

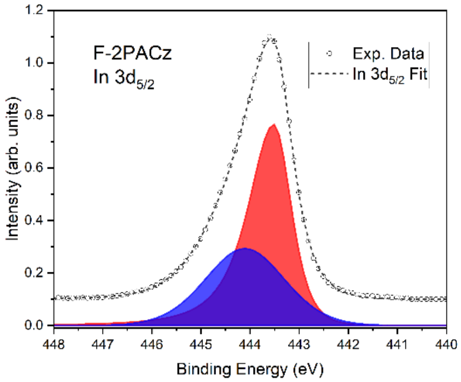

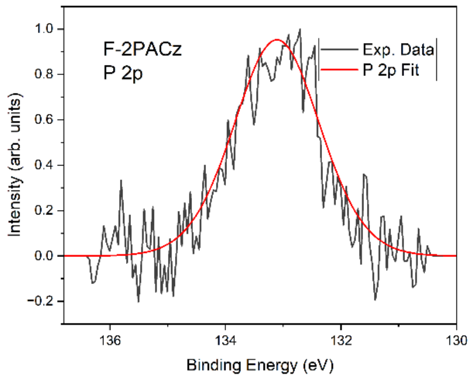

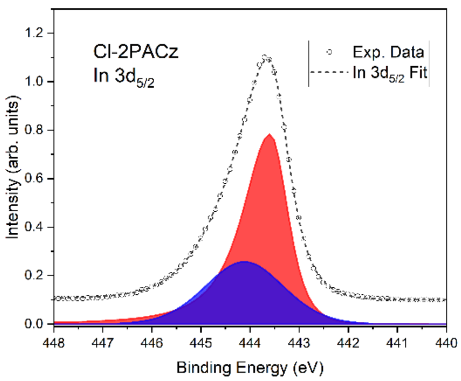

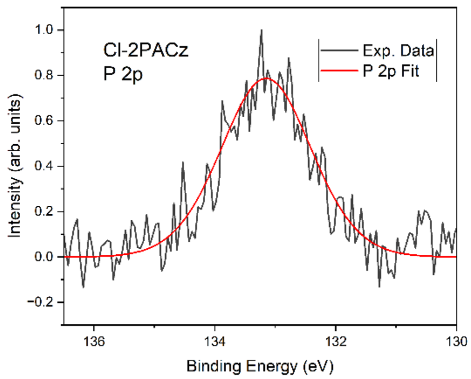

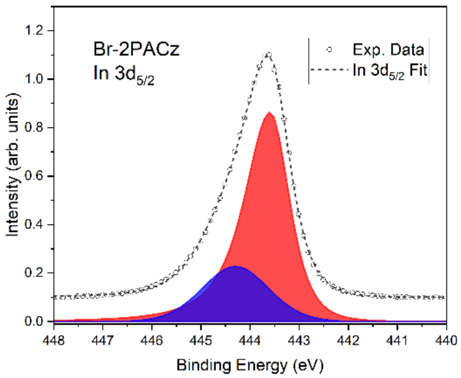
Br-2PACz
In 3d5/2
Exp. Data
In 3d5/2 Fit
Intensity (arb. units)
Binding Energy (eV)

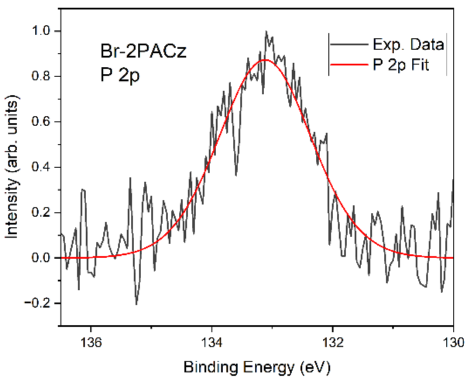
Br-2PACz
P 2p
Exp. Data
P 2p Fit
Intensity (arb. units)
Binding Energy (eV)

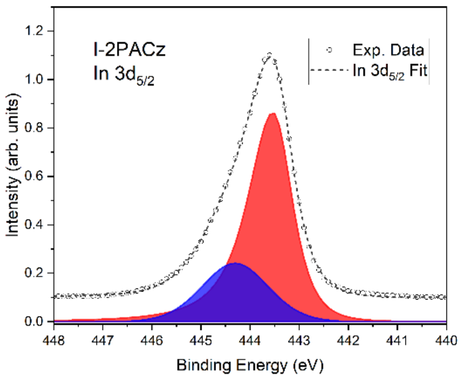
I-2PACz
In 3d5/2
Exp. Data
In 3d5/2 Fit
Intensity (arb. units)
Binding Energy (eV)

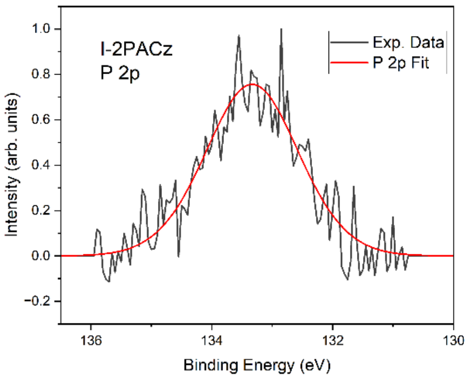
I-2PACz
P 2p
Exp. Data
P 2p Fit
Intensity (arb. units)
Binding Energy (eV)

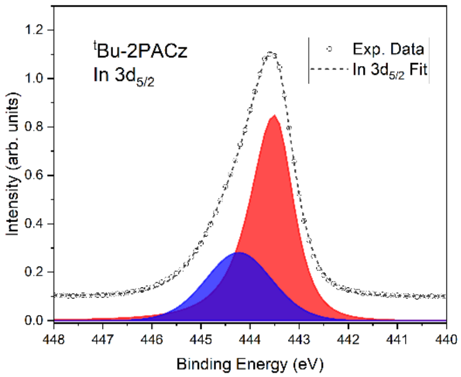
tBu-2PACz
In 3d5/2
Exp. Data
In 3d5/2 Fit
Intensity (arb. units)
Binding Energy (eV)

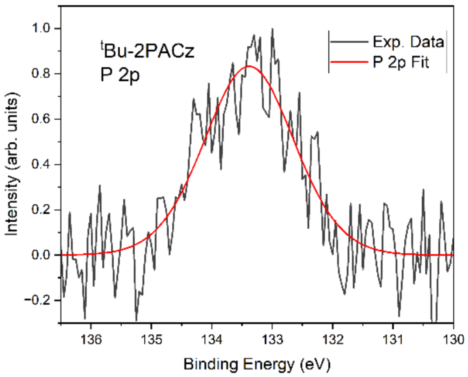
tBu-2PACz
P 2p
Exp. Data
P 2p Fit
Intensity (arb. units)
Binding Energy (eV)

References

(1) Powell, C. J.; Jablonski, A. *NIST Electron Effective-Attenuation-Length Database*, Version 1.3.; SRD 82; National Institute of Standards and Technology: Gaithersburg, MD, 2011.
(2) Lange, I.; Reiter, S.; Pätzel, M.; Zykov, A.; Nefedov, A.; Hildebrandt, J.; Hecht, S.; Kowarik, S.; Wöll, C.; Heimel, G.; Neher, D. Tuning the Work Function of Polar Zinc Oxide Surfaces Using Modified Phosphonic Acid Self-Assembled Monolayers. *Adv. Funct. Mater.* **2014**, *24* (44), 7014–7024. https://doi.org/10.1002/adfm.201401493.
(3) Khassanov, A.; Steinrück, H.-G.; Schmaltz, T.; Magerl, A.; Halik, M. Structural Investigations of Self-Assembled Monolayers for Organic Electronics: Results from X-Ray Reflectivity. *Acc. Chem. Res.* **2015**, *48* (7), 1901–1908. https://doi.org/10.1021/acs.accounts.5b00022.
(4) Dong, B.; Wei, M.; Li, Y.; Yang, Y.; Ma, W.; Zhang, Y.; Ran, Y.; Cui, M.; Su, Z.; Fan, Q.; Bi, Z.; Edvinsson, T.; Ding, Z.; Ju, H.; You, S.; Zakeeruddin, S. M.; Li, X.; Hagfeldt, A.; Grätzel, M.; Liu, Y. Self-Assembled Bilayer for Perovskite Solar Cells with Improved Tolerance against Thermal Stresses. *Nat. Energy* **2025**, *10* (3), 342–353. https://doi.org/10.1038/s41560-024-01689-2.
(5) Stöhr, J. *NEXAFS Spectroscopy*; Springer Science & Business Media, 2013.
(6) Watts, B.; Thomsen, L.; Dastoor, P. C. Methods in Carbon K-Edge NEXAFS: Experiment and Analysis. *J. Electron Spectrosc. Relat. Phenom.* **2006**, *151* (2), 105–120. https://doi.org/10.1016/j.elspec.2005.11.006.
(7) Gann, E.; McNeill, C. R.; Tadich, A.; Cowie, B. C. C.; Thomsen, L. *Quick AS NEXAFS Tool* (*QANT*): A Program for NEXAFS Loading and Analysis Developed at the Australian Synchrotron. *J. Synchrotron Radiat.* **2016**, *23* (1), 374–380. https://doi.org/10.1107/S1600577515018688.
(8) Lin, Y.; Firdaus, Y.; Isikgor, F. H.; Nugraha, M. I.; Yengel, E.; Harrison, G. T.; Hallani, R.; El-Labban, A.; Faber, H.; Ma, C.; Zheng, X.; Subbiah, A.; Howells, C. T.; Bakr, O. M.; McCulloch, I.; Wolf, S. D.; Tsetseris, L.; Anthopoulos, T. D. Self-Assembled Monolayer Enables Hole Transport Layer-Free Organic Solar Cells with 18% Efficiency and Improved Operational Stability. *ACS Energy Lett.* **2020**, *5* (9), 2935–2944. https://doi.org/10.1021/acsenergylett.0c01421.
(9) Paniagua, S. A.; Li, E. L.; Marder, S. R. Adsorption Studies of a Phosphonic Acid on ITO: Film Coverage, Purity, and Induced Electronic Structure Changes. *Phys. Chem. Chem. Phys.* **2014**, *16* (7), 2874–2881. https://doi.org/10.1039/C3CP54637C.
(10) González, G. B. Investigating the Defect Structures in Transparent Conducting Oxides Using X-Ray and Neutron Scattering Techniques. *Materials* **2012**, *5* (5), 818–850. https://doi.org/10.3390/ma5050818.
(11) Brewer, S. H.; Franzen, S. Calculation of the Electronic and Optical Properties of Indium Tin Oxide by Density Functional Theory. *Chem. Phys.* **2004**, *300* (1), 285–293. https://doi.org/10.1016/j.chemphys.2003.11.039.
(12) Powell, C. J. Practical Guide for Inelastic Mean Free Paths, Effective Attenuation Lengths, Mean Escape Depths, and Information Depths in x-Ray Photoelectron Spectroscopy. *J. Vac. Sci. Technol. A* **2020**, *38* (2), 023209. https://doi.org/10.1116/1.5141079.
(13) Powell, C. J.; Jablonski, A. *NIST Electron Inelastic-Mean-Free-Path Database*, Version 1.2.; SRD 71; National Institute of Standards and Technology: Gaithersburg, MD, 2010.

(14) Lin, Y.; Magomedov, A.; Firdaus, Y.; Kaltsas, D.; El-Labban, A.; Faber, H.; Naphade, D. R.; Yengel, E.; Zheng, X.; Yarali, E.; Chaturvedi, N.; Loganathan, K.; Gkeka, D.; AlShammari, S. H.; Bakr, O. M.; Laquai, F.; Tsetseris, L.; Getautis, V.; Anthopoulos, T. D. 18.4 % Organic Solar Cells Using a High Ionization Energy Self-Assembled Monolayer as Hole-Extraction Interlayer. *ChemSusChem* **2021**, *14* (17), 3569–3578. https://doi.org/10.1002/cssc.202100707.

(15) Sworakowski, J. How Accurate Are Energies of HOMO and LUMO Levels in Small-Molecule Organic Semiconductors Determined from Cyclic Voltammetry or Optical Spectroscopy? *Synth. Met.* **2018**, *235*, 125–130. https://doi.org/10.1016/j.synthmet.2017.11.013.

(16) Bianchi, D.; Katona, L.; Brenner, J.; Vorlaufer, G.; Vernes, A.; Werner, W. S. M. Numerical Approximation of AR-XPS Spectra for Rough Surfaces Considering the Effect of Electron Shadowing. *Surf. Interface Anal.* **2015**, *47* (1), 15–21. https://doi.org/10.1002/sia.5632.

(17) Martín-Concepción, A. I.; Yubero, F.; Espinós, J. P.; Tougaard, S. Surface Roughness and Island Formation Effects in ARXPS Quantification. *Surf. Interface Anal.* **2004**, *36* (8), 788–792. https://doi.org/10.1002/sia.1765.

(18) Kappen, P.; Reihs, K.; Seidel, C.; Voetz, M.; Fuchs, H. Overlayer Thickness Determination by Angular Dependent X-Ray Photoelectron Spectroscopy (ADXPS) of Rough Surfaces with a Spherical Topography. *Surf. Sci.* **2000**, *465* (1), 40–50. https://doi.org/10.1016/S0039-6028(00)00653-1.

(19) Paramonov, P. B.; Paniagua, S. A.; Hotchkiss, P. J.; Jones, S. C.; Armstrong, N. R.; Marder, S. R.; Brédas, J.-L. Theoretical Characterization of the Indium Tin Oxide Surface and of Its Binding Sites for Adsorption of Phosphonic Acid Monolayers. *Chem. Mater.* **2008**, *20* (16), 5131–5133. https://doi.org/10.1021/cm8014622.

(20) Tsud, N.; Yoshitake, M. Vacuum Vapour Deposition of Phenylphosphonic Acid on Amorphous Alumina. *Surf. Sci.* **2007**, *601* (14), 3060–3066. https://doi.org/10.1016/j.susc.2007.05.007.

(21) Timpel, M.; Li, H.; Nardi, M. V.; Wegner, B.; Frisch, J.; Hotchkiss, P. J.; Marder, S. R.; Barlow, S.; Brédas, J.-L.; Koch, N. Electrode Work Function Engineering with Phosphonic Acid Monolayers and Molecular Acceptors: Charge Redistribution Mechanisms. *Adv. Funct. Mater.* **2018**, *28* (8), 1704438. https://doi.org/10.1002/adfm.201704438.

(22) Peng, S.; Cao, X.; Pan, J.; Wang, X.; Tan, X.; Delahoy, A. E.; Chin, K. K. X-Ray Photoelectron Spectroscopy Study of Indium Tin Oxide Films Deposited at Various Oxygen Partial Pressures. *J. Electron. Mater.* **2017**, *46* (2), 1405–1412. https://doi.org/10.1007/s11664-016-5136-7.

(23) Shen, G.; Zhang, Y.; Juarez, J.; Contreras, H.; Sindt, C.; Xu, Y.; Kline, J.; Barlow, S.; Reichmanis, E.; Marder, S. R.; Ginger, D. S. Increased Brightness and Reduced Efficiency Droop in Perovskite Quantum Dot Light-Emitting Diodes Using Carbazole-Based Phosphonic Acid Interface Modifiers. *ACS Nano* **2025**, *19* (1), 1116–1127. https://doi.org/10.1021/acsnano.4c13036.

(24) Lin, Y.; Zhang, Y.; Zhang, J.; Marcinskas, M.; Malinauskas, T.; Magomedov, A.; Nugraha, M. I.; Kaltsas, D.; Naphade, D. R.; Harrison, G. T.; El-Labban, A.; Barlow, S.; De Wolf, S.; Wang, E.; McCulloch, I.; Tsetseris, L.; Getautis, V.; Marder, S. R.; Anthopoulos, T. D. 18.9% Efficient Organic Solar Cells Based on n-Doped Bulk-Heterojunction and Halogen-Substituted Self-Assembled Monolayers as Hole Extracting Interlayers. *Adv. Energy Mater.* **2022**, *12* (45), 2202503. https://doi.org/10.1002/aenm.202202503.
